# Assessing the potential of genetic resource introduction into elite germplasm: a collaborative multiparental population for flint maize

**DOI:** 10.1007/s00122-023-04509-5

**Published:** 2024-01-12

**Authors:** Dimitri Sanchez, Antoine Allier, Sarah Ben Sadoun, Tristan Mary-Huard, Cyril Bauland, Carine Palaffre, Bernard Lagardère, Delphine Madur, Valérie Combes, Stéphane Melkior, Laurent Bettinger, Alain Murigneux, Laurence Moreau, Alain Charcosset

**Affiliations:** 1 Université Paris-Saclay, INRAE, CNRS, AgroParisTech, Génétique Quantitative et Evolution–Le Moulon, 91190 Gif-Sur-Yvette, France; 2 Present Address: Syngenta, 12 Chemin de L’Hobit, 31790 Saint-Sauveur, France; 3 Université Paris-Saclay, AgroParisTech, INRAE, UMR MIA-Paris Saclay, 91120 Palaiseau, France; 4 UE 0394 SMH, INRAE, 2297 Route de l’INRA, 40390 Saint-Martin-de-Hinx, France; 5 RAGT2n, 12510 Druelle, France; 6 Lidea France, Avenue Gaston Phoebus, 64230 Lescar, France; 7 Limagrain Europe, 28 Route d’Ennezat, 63720 Chappes, France

## Abstract

**Key message:**

Implementing a collaborative pre-breeding multi-parental population efficiently identifies promising donor x elite pairs to enrich the flint maize elite germplasm.

**Abstract:**

Genetic diversity is crucial for maintaining genetic gains and ensuring breeding programs’ long-term success. In a closed breeding program, selection inevitably leads to a loss of genetic diversity. While managing diversity can delay this loss, introducing external sources of diversity is necessary to bring back favorable genetic variation. Genetic resources exhibit greater diversity than elite materials, but their lower performance levels hinder their use. This is the case for European flint maize, for which elite germplasm has incorporated only a limited portion of the diversity available in landraces. To enrich the diversity of this elite genetic pool, we established an original cooperative maize bridging population that involves crosses between private elite materials and diversity donors to create improved genotypes that will facilitate the incorporation of original favorable variations. Twenty donor × elite BC1S2 families were created and phenotyped for hybrid value for yield related traits. Crosses showed contrasted means and variances and therefore contrasted potential in terms of selection as measured by their usefulness criterion (UC). Average expected mean performance gain over the initial elite material was 5%. The most promising donor for each elite line was identified. Results also suggest that one more generation, i.e., 3 in total, of crossing to the elite is required to fully exploit the potential of a donor. Altogether, our results support the usefulness of incorporating genetic resources into elite flint maize. They call for further effort to create fixed diversity donors and identify those most suitable for each elite program.

**Supplementary Information:**

The online version contains supplementary material available at 10.1007/s00122-023-04509-5.

## Introduction

The release of new varieties with constantly improved genetic values has efficiently contributed to the augmentation of the yield production that was needed to meet the increasing demand for agricultural products during the last decades (Lobell et al. 2011; Welcker et al. 2022). This demand will continue to increase with the growth of the human population and the diversification of agricultural product uses (Goddard 2009; Tester and Langridge 2010). This necessitates that plant breeders maintain their programs’ genetic gain, while also selecting for adaption to more environment friendly practices such as agroecology (Wezel et al. 2014). Genetic diversity of a breeding population is one of the key drivers of genetic gain as it is a determinant of trait genetic variance, which controls expected response to selection per generation (Lush 1937). Elite germplasm in major cultivated species generally has a narrow genetic basis because modern breeding has exploited only part of the genetic variability that was available in traditional varieties (Maccaferri et al. 2003; Palmgren et al. 2015). During breeding cycles, new lines and varieties are derived mostly from a limited number of crosses between selected elite lines, which further decreases elite genetic diversity (Reif et al. 2005; Mikel and Dudley 2006). This was illustrated, for example, by Allier et al. (2019) who reported a genetic diversity drop over time in a maize breeding program. Recent selection methods such as genomic selection may worsen diversity depletion in the absence of specific constraints (Jannink 2010; Rutkoski et al. 2015; Lin et al. 2016). These trends toward a decreasing diversity level are expected to restrain future genetic gain and may hamper the ability of breeding programs to address new selection objectives related to climate change and evolutions in agronomical practices (McCouch et al. 2013; Mickelbart et al. 2015). Managing diversity in breeding programs can delay its loss and preserve, to some extent, long-term genetic gain (Allier et al. 2020a). Nevertheless, introduction of external diversity sources is required to bring back genetic variation in breeding programs and counterbalance the negative impact of breeding on genetic diversity in elite germplasm (Wray and Goddard 1994; Meuwissen 1997; Woolliams et al. 2015; Allier et al. 2020a).

Since the beginning of modern selection, an intense effort has been made to collect and store diversity sources such as wild relatives, exotic germplasms, landraces, and first-cycle inbred lines (developed from landraces). Gene banks keep these accessions available to breeders thanks to ex situ and in situ conservation (Wang et al. 2017). Compared with elite materials, these resources present a higher genetic diversity and carry original potentially favorable alleles (Maccaferri et al. 2003; Palmgren et al. 2015). They provide a source of alleles to deal with yield stability and abiotic stress but they suffer from lower performances than elite lines (performance gap) due to unfavorable alleles that have been eliminated in elite materials by recent selection and may not be adapted to local conditions (Strigens et al. 2013; Dwivedi et al. 2016). For monogenic and oligogenic traits, using targeted marker-assisted backcross has been a way to introgress favorable alleles from genetic resources into an elite background genome without being hampered by the performance gap (Visscher et al. 1996; Hospital and Charcosset 1997; Frisch and Melchinger 2005). Experimental studies confirm the efficiency of this approach to recover the elite germplasm after single or multiple introgression events (Peng et al. 2014; Han et al. 2017). This backcross procedure has also helped to improve polygenic traits controlled by a few major genomic regions, such as flowering time and yield components under drought conditions in maize (Ribaut and Ragot 2006). However, the success of the introgression may be compromised by the presence of unfavorable alleles in the residual donor genome or negative interactions with the recipient background (Hospital 2005).

The backcross procedure is based on the incorporation of favorable variation at some genomic regions to improve the value of the targeted trait. Its implementation is difficult for quantitative traits which are determined by many regions with minor effects (e.g., grain yield in maize). In this case, the favorable donor alleles are not easily identifiable and they may be eliminated during the backcrossing process (Cowling 2013). Simmonds (1993) described an alternative genetic resource utilization strategy adapted to the improvement of quantitative traits, called “incorporation” (in opposition to “introgression”). In this case, the objective is not to introgress a few targeted genomic regions but to broaden the genetic basis of the elite programs thanks to the incorporation of extrinsic polygenic favorable variations. Simmonds (1993) proposed to first improve genetic resources by recurrent selection to reduce the performance gap with the elite material, i.e., conduct pre-breeding. A recent pre-breeding program aiming at improving flint landraces illustrated the potential but also the difficulties of this process (Ordás et al. 2023). If the pre-breeding progenies still underperform elite, they can be crossed with elite lines to produce a specific buffer population that complements the elite program, i.e., implement a bridging population. The best bridging individuals become potential parents for introduction in the elite breeding program. This strategy aims to close the gap in performance and limit diversity donor introduction’s negative impact on short-term genetic gain. In maize, after a number of preliminary unsuccessful efforts reported by Simmonds et al. (1993), this strategy was implemented to incorporate tropical diversity in temperate North-American germplasm by MM Goodman (Lewis and Goodman 2003) and then in the Germplasm Enhancement of Maize (GEM) project (see below). This illustrates that the efforts and methods dedicated to the pre-breeding and bridging populations must be tuned according to the considered genetic resources (Allier et al. 2020a; Sanchez et al. 2023).

The breeding potential of crosses between donor and elite lines in a bridging population is determined by their abilities to generate transgressive individuals. This question can be addressed considering the usefulness criterion concept (UC), which represents the expected performance of the cross progeny after selection (Schnell and Utz 1976). This expected performance is determined by the progeny genetic mean (*μ*), the selection intensity (*i*), the selection accuracy (*h*) and the progeny genetic standard deviation (*σ*$$)$$ as: UC_1_=*μ*+*ihσ*. Wolfe et al. (2021) proposed to take only the additive part of the genetic variance in the progenies (UC_2_=*μ*+*ihσ*_a_ here *σ*_a_ is the progeny additive genetic standard deviation). UC_2_ is meaningful to evaluate the interest of the progeny as parents of a new breeding cycle. Adding back-cross generations with the elite parent is expected to diminish the gap in performance at the cost of a reduction in variance, with an optimum in terms of UC values that needs to be determined.

For a breeding company, allocating time and budget to screen potential donors and deploy pre-breeding and bridging approaches causes a reduction of the efforts devoted to the elite breeding programs, which can reduce short-term competitiveness (Pollak 2003). This has prompted the establishment of pre-competitive collaborations to share costs between public institutes and private companies. For instance, the Latin American Maize Project (LAMP, Pollak 1990; Salhuana et al. 1997; Salhuana and Pollak 2006) was crucial for characterizing and evaluating the maize Latin American tropical germplasm accessions. This international project, involving 12 countries, provided breeders with useful yield evaluation and agronomical characterization of these accessions (Salhuana et al. 1997). Later, the Germplasm Enhancement of Maize project (GEM) was conducted to integrate the LAMP accessions in the US elite germplasm. In this project, bridging individuals were derived from three-way crosses involving each a LAMP accession and two elite lines from different private companies (Pollak 2003). For the first time, maize proprietary inbred materials were used in an extensive collaborative program. This project led to the creation of original lines adapted to the Southern and Midwest USA photoperiod conditions (Wang et al. 2017). Other public–private initiatives inspired by the GEM project have emerged, such as the Soybean Asian Variety Evaluation (SAVE) project to characterize Asian soybean varieties (Manjarrez-Sandoval et al. 1998). Sharma and Carena (2012) recorded the use of GEM materials to create dent inbred lines well adapted to the North Dakota conditions.

Other maize germplasms may benefit from similar initiative, among which the European flint group. This group is characterized by early vigor and cold adaptation. It is commonly crossed with the dent pool to create performing hybrid varieties adapted to the Northern European environmental conditions (Messmer et al. 1993; Cartea et al. 1999; Böhm et al. 2017). Results based on haplotyping illustrate that a significant fraction of the diversity of landraces has not been exploited to form the flint breeding pool (Mayer et al. 2020). This bottleneck effect has been amplified by the extensive use of a few first-cycle lines (F2, F7, EP1, DK105, etc.) to create the elite flint lines used in modern breeding (Messmer et al. 1992; Dubreuil and Charcosset 1999; Stich et al. 2005; Van Inghelandt et al. 2010). The preservation of lines derived from European maize landraces and historical lines has made possible to create several diversity panels (Camus-Kulandaivelu et al. 2006; Rincent et al. 2014) later enriched to form a collection of 1191 inbred flint lines (Gouesnard et al. 2017). This collection appears as a reservoir of potential diversity donors, which can be harnessed to incorporate new favorable variations in the elite flint lines and preserve the efficiency of the dent × flint heterotic pattern.

The objective of this study was to document experimentally the potential of genetic resources to bring suitable variation into the flint elite programs. To do so, we established a cooperative pre-breeding multi-parental population. This population consists of a total of 20 BC1 connected families issued from crosses between (i) diversity donor lines chosen for their originality and initial performance and (ii) elite recipient lines from different private partners. For each family, we compared models to estimate the within-family mean performances and the genetic variances to determine their UCs. Results showed that most crosses have the potential to generate positive transgressive materials. It also revealed contrasted variances and UCs among crosses and made it possible to identify the most promising donor for each partner. Finally, our results suggest that one more generation of crossing to elite should in general be beneficial to fully exploit the potential of the genetic resources which were used.

## Material and methods

### Plant material

The experimental material consisted of flint maize BC1S2:3 individuals derived from donors and seven recipient lines. Recipient lines were flint elite material genotypes, each provided by a different partner (Limagrain, RAGT2n, KWS, Masseed, Euralis, Caussade and INRAE). These lines were named A1, A2, A3, A4, A5, A6 and A7 (each number was randomly assigned to one of the partners). Each partner also supplied a dent line complementary to its flint line (referred to as the tester line in the following). Each pair was previously selected by its owner to provide a performing hybrid adapted to the cultivation in the B-C1 French grain precocity group area (early-mid early).

We pre-selected, as potential donors, 74 lines of the same precocity group from a collection of 1191 lines representative of the flint diversity (Gouesnard et al. 2017). The test-cross yield performances of potential donors were evaluated by each private partner using their own tester line and field network (for further information, see File S1). The estimation of donor General Combining Ability (GCA) and other criteria (no agronomic default, no lodging, etc.) led to the selection of seven donor lines (described in Table S1) after discussion among the different partners. Donor lines were named D1, D2, D3, D4, D5, D6 and D7.

**Fig. 1:**
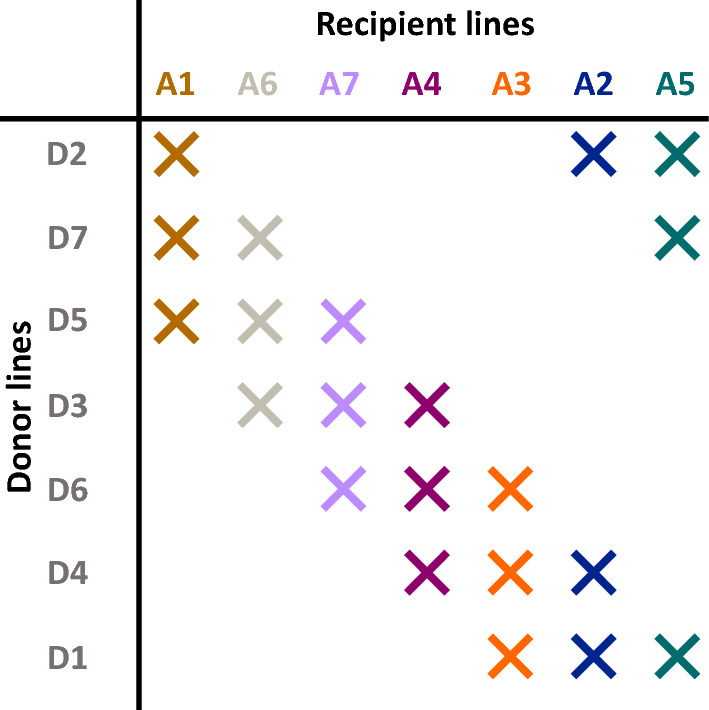
Incomplete crossing design between donor lines and recipient lines. Twenty-one F1 single-cross individuals were created by donor × recipient (D×R) crosses (visualized by a cross). Each donor line was crossed with three recipient lines and each recipient line was crossed with three donor lines. Each F1 individual was backcrossed with its recipient parent to produce a BC1 population. For each D × R cross, 60 BC1 plants were self-pollinated during two generations (Single Seed Descent process) to obtain BC1S2 individuals

Recipient and donor lines were crossed according to an incomplete factorial design discussed in a concerted way among partners (Fig. 1). This design was chosen to maximize the connectivity between crosses (i.e., maximize the number of cross failures necessary to disconnect the crossing design into two independent ones). Each F1 single-cross hybrid created by crossing a donor (D) and a recipient line (R) was backcrossed with the recipient line to produce BC1 populations. For each D×R cross, 60 BC1 plants were self-pollinated during two generations (single seed descent process, SSD) to obtain BC1S2 individuals. A total of 1174 BC1S2 plants was obtained due to some failures during the SSD process. The numbers of D×R crosses (21) and of BC1S2 individuals by cross were guided by statistical considerations (e.g., the minimal required number of BC1S2 individuals to estimate the genetic variance of a D×R cross) and total experimental means available. In order to perform testcross phenotypic evaluation (see section *Plant Phenotyping*), each BC1S2 plant was further self-pollinated to generate a BC1S2:3 progeny.

### Plant genotyping

The BC1S2 individuals and the parental lines, with the exception of D3 and D7, were genotyped using a customized Maize Illumina Infinium 25K SNP XT array. D3 and D7 were genotyped with the Maize Illumina Infinium 50K SNP array (Ganal et al. 2011). Thanks to the overlap between both arrays (16587 markers) and the 25K genotyping of their progenies, we imputed the D3 and D7 25K genotyping. The percentage of incoherent markers between parental and progeny genotyping, called illegitimate rate, was computed. Sixteen BC1S2 individuals were discarded from the dataset because of a high illegitimate rate (> 5%). For A1×D2, A1×D7 and A2×D1 crosses, the average illegitimate rate was initially 8.78%, 7.65% and 8.87%, respectively, which was higher than in other families (0.52% on average). Multiple correspondence analysis (MCA, Fig. S1) showed that D2 and D7 were not the actual donor parents of crosses called A1×D2 and A1×D7, suggesting a labelling error at the beginning of the process. For both crosses, 25K genotyping of a virtual donor line was constructed thanks to the genotyping of A1 and of the BC1S2 individuals. This virtual donor line was compared by identity by state (IBS) with the 74 candidate donor lines. Thus, the QSF3_inra line was identified as the actual A1 × D2 donor line; it is named D8 in the following. The A1 × D7 donor line was not identified among the 74 lines; the virtual donor line was considered as an additional donor line, named D9 in the following. So, the two corresponding crosses were named as A1 × D8 and A1 × D9. Lastly, genotypic frequencies of BC1S2 individuals coming from the A2 × D1 cross suggested a complex pedigree involving two recipients instead of one (i.e., a three-way cross). This cross was removed from the dataset for variance decomposition analysis (see below). Considering the updated pedigree, genotypic missing data were imputed using AlphaPlantImpute software v1.1 (Gonen et al. 2018). Parameters used for imputation are provided in File S2.

### Plant phenotyping

BC1S2:3 individuals were crossed to the dent tester line associated to the recipient line to produce hybrid progenies. In the following, hybrids derived from a same RxD cross are considered as a family. Hybrids were evaluated in 3 different locations in France (Blois, Loir-et-Cher; Saint-Martin de Hinx, Landes; Villers, Pas-de-Calais) in 2019 (further referred to as Blo19, Smh19 and Vil19). Each trial was composed of 1512 elementary two-row plots with an area of 9.6 m^2^, 9.3 m^2^ or 10.9 m^2^, for Blo19, Smh19 and Vil19, respectively. Plant density was 9.4, 8.6 and 9.2 seed.m^−2^, respectively. Hybrids derived from the same recipient line were gathered in a same sub-trial to minimize experimental errors in the evaluation of hybrids derived from the same recipient line. To balance the number of plots allocated to each recipient line, each sub-trial had the same dimension (216 plots). Each sub-trial was divided into 12 blocks. In each block, a commercial hybrid (ADEVEY) and the reference hybrid, corresponding to the single-cross hybrid between the recipient line and the tester line, were sown and considered as checks. In each sub-trial, the average number of experimental hybrid repetitions varied from 1.07 to 1.56 depending of the family. Hybrid randomization in each sub-trial was performed using the nursery function of the Digger R package to ensure a homogeneous repartition of checks and repeated hybrids in each sub-trial (Coombes 2009).

Hybrids were phenotyped for male and female flowering times (FLOM and FLOF in days after planting), anthesis-silking interval (ASI in days), grain yield at 15% moisture (GY in Mg/ha), grain humidity at harvest (H_2_O in %) and plant height (PH in cm). FLOM and FLOF are the day at which 50% of the plants exhibited anthers or silks, respectively. ASI is the difference in days between FLOM and FLOF. PH was not measured in Vil19. In Blo19 and Smh19, plots with a number of plants lower than the median number minus 15 plants were eliminated. In Vil19, final plant density was not recorded and fresh grain weight was used as a proxy to filter aberrant plots (plots with a fresh grain weight bellow 7 kg were eliminated). A lower germination rate was observed in the A7 sub-trial which led to the elimination of 33%, 28% and 14% of plots in this sub-trial in Blo19, Smh19 and Vil19, respectively. On average, 1.3% of the data were eliminated in the other sub-trials.

The raw phenotypic data were corrected for spatial effects predicted using AR1 × AR1 models (see File S3for details). These models were fitted using ASREML-R v4 (Butler et al. 2017). A commercial grain yield index (YI) was calculated afterwards according to the following index:

$${\text{YI}}_{m} = {\text{GY}}_{m} + 0.25 * \left( {{\text{H}}_{2} {\text{O}}_{{{\text{ADEVEY}}}} - {\text{H}}_{2} {\text{O}}_{m} } \right)$$

where GY_*m*_ and H_2_O_*m*_ are the corrected grain yield and humidity values of plot *m* and H_2_O_ADEVEY_ is the trial mean grain humidity value of the check hybrid ADEVEY. This index corresponds to the one usually considered for variety registration in France. It penalizes the experimental hybrids that mature later than the reference (i.e., ADEVEY).

### Variance decomposition

Different variance decomposition models were used for multi-trial analysis (Table 1). They allowed us to estimate the total genetic variance and the additive genetic variance, in the whole population and within each family. The variance–covariance matrix of parameter estimates was computed and used to test the effects of recipients and donors on these parameters. Parameters of all models were estimated with the package MM4LMM in R (Laporte et al. 2022).

### Estimation of global genetic variance

The following model was used to estimate global genetic variance:

$${\text{Y}}_{ilhm} = { }\mu + { }\alpha_{i} + \tau_{l} + { }\left( {\alpha \tau } \right)_{il} + {\text{ G}}_{h} + {\text{GE}}_{ih} + e_{ilhm} { }\left( {{\text{M}}\_{\text{G}}} \right)$$

$$\begin{gathered} {\text{G}}\sim {\text{N}}\left( {0,\;I\sigma _{G}^{2} } \right),{\text{ GE}}_{i} \sim {\text{N}}\left( {0,\;I\sigma _{{GE_{i} }}^{2} } \right)\;{\text{ind}}, \hfill \\ e_{i} \sim {\text{N}}\left( {0,\;I\sigma _{{e_{i} }}^{2} } \right)\;{\text{ind,}}\quad {\text{G}}\ \bot \ {\text{GE}}_i\ \bot\ e_i \hfill \\ \end{gathered}$$

where ${\mathrm{Y}}_{ilhm}$ is the phenotypic value of the repetition $m$ of check $l$ or experimental hybrid $h$ in trial $i$. ${\alpha }_{i}$ is the fixed effect of the trial$,$ ${\tau }_{l}$ is the fixed genetic effect accounting for the difference between checks (7 reference hybrids and ADEVEY) and experimental hybrids (a factor with 9 levels: one level for each check and a supplemental level for the experimental hybrids),${\left(\alpha \tau \right)}_{il}$ is an interaction term between the trials and the checks, ${\mathrm{G}}_{h}$ is the random genetic effect of experimental hybrids, ${\mathrm{GE}}_{ih}$ is an interaction term between the trial $i$ and the experimental hybrid $h$ and ${e}_{ilhm}$ is the error term (the errors are considered independent between trials). Symbol ($\perp$) indicates random effects which are considered independent of each other. In the following, this model is referred to as M_G (Table 1).

**Table 1:** Summary of fixed and random effects declared to model genetic effects in the presented linear mixed models of variance decomposition

Model	Pedigree structure in fixed part	Random genetic effects	
		Genetic (or permanent) effect	Additive effect
M_G		Global	–
M_FG_S_	✓	Family specific	–
M_FG	✓	Global	–
M_FA_S_P_S_	✓	Family specific	Family specific
M_FA_S_	✓	–	Family specific
M_FAP	✓	Global	Global
M_FA	✓	–	Global

The second column indicates the presence of a fixed recipient effect and a fixed family effect in the model. The third column sums up how the random genetic effects are declared in each model

### Estimation of within-family genetic variance

Each hybrid family was characterized by a recipient line (with its associated tester) and a donor line. In the following models, the pedigree structure was modeled by two fixed effects: a recipient effect and a family (recipient × donor combination) effect. As each tester was associated to a specific recipient line, the recipient effect captured the effect of a recipient-tester combination.

The following model was used to estimate the within-family genetic variances:

$$\begin{gathered} {\text{Y}}_{{ijkhm}} = \mu + \alpha _{i} + \rho _{j} + \theta _{k} + \left( {\alpha \rho } \right)_{{ij}} + \left( {\alpha \theta } \right)_{{ik}} + {\text{ G}}_{{kh}} \hfill \\ \quad \quad \quad\quad + {\text{GE}}_{{ikh}} + e_{{ijkhm}} \;\left( {{\text{M}}\_{\text{FG}}_{{\text{S}}} } \right) \hfill \\ \end{gathered}$$

$$\begin{gathered} {\text{G}}_{k} \sim {\text{N}}\left( {0,\;I\sigma _{{G_{k} }}^{2} } \right){\text{ ind}},\;{\text{GE}}_{{ik}} \sim {\text{N}}\left( {0,\;I\sigma _{{GE_{{ik}} }}^{2} } \right){\text{ ind}}, \hfill \\ e_{i} \sim {\text{N}}\left( {0,\;I\sigma _{{e_{i} }}^{2} } \right){\text{ ind}},\;{\text{G}}_{k}\ \bot\ {\text{GE}}_{{ik}}\ \bot\ e_{i} \hfill \\ \end{gathered}$$

where ${\mathrm{Y}}_{ijkhm}$ is the phenotype of the repetition $m$ of hybrid $h$ in family $k$ derived from the recipient line $j$ (or the recipient line if the hybrid is a reference hybrid) in trial $i$. ${\alpha }_{i}$ is the fixed effect of the trial, ${\rho }_{j}$ is the fixed effect of the recipient line (a factor with 8 levels: one level for each recipient line and an additional level for ADEVEY) and ${\theta }_{k}$ is the fixed effect of the family (a factor with 21 levels: one level for each hybrid family and an additional level for ADEVEY and the reference hybrids). ${\left(\alpha \rho \right)}_{ij}$ is a fixed interaction term between the trial $i$ and the recipient $j$ and ${\left(\alpha \theta \right)}_{ik}$ is a fixed interaction term between the trial $i$ and the family $k$. ${\mathrm{G}}_{kh}$ is a random genetic effect (with a specific variance per family) and ${\mathrm{GE}}_{ikh}$ is a random interaction term between the trial $i$ and the genotype $h$. ${e}_{ijkhm}$ is the error term. In the following, this model is referred to as M_FG_S_ (Table 1).

The same model was also fitted considering that the genetic effect and the associated interaction term had variances common to all families. It is referred to as M_FG in the following (Table 1). Multi-trial adjusted means were estimated for each hybrid using an alternative M_FG model considering the hybrid genetic effect as fixed. For each trait, the difference between the multi-trial mean values of each family and the value of its reference hybrid has been tested following the procedure described in the File S4.

### Estimation of within-family additive genetic variance

The within-family additive variances were estimated thanks to the following model:

$$\begin{gathered} {\text{Y}}_{{ijkhm}} = \mu + \alpha _{i} + \rho _{j} + \theta _{k} + \left( {\alpha \rho } \right)_{{ij}} + \left( {\alpha \theta } \right)_{{ik}} + {\text{A}}_{{kh}} + {\text{P}}_{{kh}} \hfill \\ \quad \quad \quad\quad + {\text{AE}}_{{ikh}} + {\text{PE}}_{{ikh}} + e_{{ijkhm}} \;\left( {{\text{M}}\_{\text{FA}}_{{\text{S}}} {\text{P}}_{{\text{S}}} } \right) \hfill \\ \end{gathered}$$.

$$\begin{gathered} {\text{A}}_{k} \sim {\text{N}}\left( {0,\;K_{k} \sigma _{{{\text{A}}_{k} }}^{2} } \right)\;{\text{ind}},\;{\text{P}}_{k} \sim {\text{N}}\left( {0,\;I\sigma _{{{\text{P}}_{k} }}^{2} } \right)\;{\text{ind}},\;{\text{AE}}_{{ik}} \sim {\text{N}}\left( {0,\;K_{k} \sigma _{{{\text{AE}}_{{ik}} }}^{2} } \right), \hfill \\ {\text{PE}}_{{ik}} \sim {\text{N}}\left( {0,\;I\sigma _{{{\text{PE}}_{{ik}} }}^{2} } \right)\;{\text{ind}},\;e_{i} \sim {\text{N}}\left( {0,\;I\sigma _{{e_{i} }}^{2} } \right)\;{\text{ind}},\;{\text{and}}\;{\text{A}}_{k}\ \bot\ {\text{P}}_{k}\ \bot\ {\text{AE}}_{{ik}}\ \bot\ {\text{ PE}}_{{ik}}\ \bot\ e_{i} \hfill \\ \end{gathered}$$

The fixed terms of this model were similar to the fixed terms of the model (M_FG_S_). The random part of the model was composed of an additive genetic effect ${\mathrm{A}}_{kh}$, a genetic permanent effect ${\mathrm{P}}_{kh}$, an interaction term between the trials and the additive genetic effect ${\mathrm{AE}}_{ikh}$ and an interaction term between the trials and the genetic permanent effect ${\mathrm{PE}}_{ikh}$. ${e}_{ijkhm}$ is the error term. The permanent effect modeled non-additive genetic effects within each family. It can be estimated because the experimental hybrids have been partially repeated. A specific variance was estimated per family for each random term. This model is referred to as M_FA_S_P_S_ (Table 1).

In this model, the variance–covariance matrix of additive terms depends on marker-based kinship matrices (${K}_{\mathrm{k}}$). Each ${K}_{\mathrm{k}}$ matrix was specific to a family. Its coefficients were computed using the genotyping of the BC1S2 individuals derived from the cross, according to the Natural and Orthogonal Interaction Approach (NOIA,Álvarez-Castro and Carlborg 2007), as recommended by Vitezica et al. (2017). For a given individual $i$ (of the family $k$), at a given marker $j$, coefficients were calculated using genotypic frequencies as follows:

$$h_{{{\text{A}}_{ijk} }} = \left\{ {\begin{array}{*{20}l} { - \left( { - p_{{{\text{Bb}}_{jk} }} - 2p_{{{\text{bb}}_{jk} }} } \right)} \hfill \\ { - \left( {1 - p_{{{\text{Bb}}_{jk} }} - 2p_{{{\text{bb}}_{jk} }} } \right)} \hfill \\ { - \left( {2 - p_{{{\text{Bb}}_{jk} }} - 2p_{{{\text{bb}}_{jk} }} } \right)} \hfill \\ \end{array} } \right.{\text{ for genotypes}}\left\{ {\begin{array}{*{20}l} {{\text{BB}}} \hfill \\ {{\text{Bb}}} \hfill \\ {{\text{bb}}} \hfill \\ \end{array} } \right.$$

where ${{p}_{\mathrm{Bb}}}_{jk}$ and ${p}_{{\mathrm{bb}}_{jk}}$ are the frequencies of genotypes Bb and bb in the family $k$, respectively.

Additive kinship matrices were obtained with the formula:

$$K_{{\text{k}}} = \frac{{H_{{{\text{A}}_{k} }} H_{{{\text{A}}_{k} }}^{\prime } }}{{tr\left( {H_{{{\text{A}}_{k} }} H_{{{\text{A}}_{k} }}^{\prime } } \right)/n_{k} }}$$

where *n*_*k*_ is the number of individuals in family *k* and

$$H_{{{\text{A}}_{k} }} = \left( {\begin{array}{*{20}c} {h_{{{\text{A}}_{11k} }} \cdots h_{{{\text{A}}_{{1m_{k} k}} }} } \\ { \vdots { } \cdots { } \vdots } \\ {h_{{{\text{A}}_{n1k} }} \cdots h_{{{\text{A}}_{{nm_{k} }} }} } \\ \end{array} } \right)$$

where ${m}_{k}$ is the number of polymorphic markers in the family $k$.

To test the pertinence of the permanent effect in this context, we also fitted a sub model M_FA_S_ including only the additive genetic effects (${\mathrm{A}}_{k}$ and ${\mathrm{AE}}_{ik}$). We considered also two other models (M_FA and M_FAP) where the additive and permanent effect variances were declared homogeneous between families (Table 1).

### Heritability estimation

A multi-trial heritability was computed for each trait using the variance parameter estimates of the M_G model:

$$H^{2} = \frac{{\sigma_{{\text{G}}}^{2} }}{{\sigma_{{\text{G}}}^{2} + \frac{{\overline{{\sigma_{{{\text{GE}}}}^{2} }} }}{{n_{{{\text{trial}}}} }} + \frac{{\mathop \sum \nolimits_{i = 1}^{{n_{{{\text{trial}}}} }} \sigma_{{e_{i} }}^{2} /n_{{{\text{rep}}_{i} }} }}{{n_{{{\text{trial}}}} }}}}$$

where ${\sigma }_{\mathrm{G}}^{2}$ is the genetic variance, $\overline{{\sigma }_{\mathrm{GE}}^{2}}$ is the average of the genotype × trial variances. ${n}_{\mathrm{trial}}$ is the number of trials (${n}_{\mathrm{trial}}=3$), ${\sigma }_{{e}_{i}}^{2}$ is the error variance in the trial $i$ and ${n}_{{\mathrm{rep}}_{i}}$ is the mean number of repetition in the trial $i$ (Piepho and Möhring 2007).

#### Usefulness criterion calculation

For performance traits (GY and YI), we computed two usefulness criteria (UC_1_ and UC_2_) to provide information about the expected response to selection in each family *k*:

$${\text{UC}}_{1k} = \hat{\mu }_{k} + ih\hat{\sigma }_{{{\text{G}}_{k} }}$$

$${\text{UC}}_{2k} = { }\hat{\mu }_{k} + ih\hat{\sigma }_{{{\text{A}}_{k} }}$$

where $\hat{\mu }_{k}$ is the adjusted mean for the family effect in the model M_FA_S_P_S_, $\hat{\sigma }_{{{\text{A}}_{k} }}^{2}$ is the additive variance and $\hat{\sigma }_{{{\text{G}}_{k} }}^{2} = \hat{\sigma }_{{{\text{A}}_{k} }}^{2} + \hat{\sigma }_{{{\text{P}}_{k} }}^{2}$ is the total genetic variance where $\hat{\sigma }_{{{\text{P}}_{k} }}^{2}$ is the permanent effect variance. $\hat{\sigma }_{{{\text{A}}_{k} }}^{2}$ and $\hat{\sigma }_{{{\text{P}}_{k} }}^{2}$ were estimated with the model M_FA_S_P_S_. $h$ is the selection accuracy and $i$ is the selection intensity. The selection accuracy was assumed to be one, as would be the case when selecting directly on genetic effects (Zhong and Jannink 2007). Usefulness criteria were calculated with $i=2.07$ (selection rate of 5%).

UC_1*k*_ is an estimation of the expected performance of individuals selected in the family *k*. UC_2*k*_ is an estimation of the expected additive value transmitted by selected individuals to the next generation.

## Results

### Genetic variance and multi-trial heritability

Global variance components were estimated thanks to the model M_G (Table 2). The broad-sense heritability was high for all traits. Flowering traits (FLOM and FLOF) had higher heritabilities (0.82 and 0.86) than GY (0.68). FLOM had a lower genetic variance than FLOF. For flowering traits, we noticed a lower G × E variance and a stronger error term variance for trial Vil19. For PH, variance decomposition revealed a clear difference between the two trials where this trait was measured, error variance in Blo19 being more than four times larger than in Smh19. We observed a similar trend for G × E terms. YI and GY had similar heritabilities and genetic variances. However, YI had stronger G × E variances than GY. For both YI and GY, the Smh19 trial showed higher error variances than the two other trials.

**Table 2:** Variance decomposition with the models M_G (genetic effects modelled as a global genetic random effect) and M_FG (addition of recipient and family fixed effects) for each trait

Trait	Model	AIC	BIC	H^2^	Variance components						
					${\sigma }_{\mathrm{G}}^{2}$	${\sigma }_{\mathrm{GE}}^{2}$			${\sigma }_{e}^{2}$		
						Blo19	Smh19	Vil19	Blo19	Smh19	Vil19
FLOM	M_G	5684	5738	0.82	**1.40** (0.07)	**0.62** (0.09)	**0.63** (0.06)	**0.02** (0.08)	**0.67** (0.06)	**0.26** (0.02)	**0.91** (0.07)
	M_FG	4963	5109	–	**0.94** (0.05)	**0.20** (0.07)	**0.37** (0.04)	**0** (−)	**0.66** (0.05)	**0.27** (0.02)	**0.89** (0.04)
FLOF	M_G	6468	6523	0.86	**2.30** (0.11)	**1.15** (0.1)	**0.40** (0.07)	**0.22** (0.08)	**0.57** (0.05)	**0.39** (0.03)	**0.85** (0.07)
	M_FG	5640	5785	–	**1.28** (0.07)	**0.45** (0.07)	**0.33** (0.06)	**0.15** (0.08)	**0.57** (0.05)	**0.39** (0.03)	**0.87** (0.07)
ASI	M_G	2934	2989	0.63	**0.51** (0.02)	**0.55** (0.07)	**0** (−)	**0.72** (0.1)	**0.62** (0.05)	**0.01** (0)	**1.05** (0.08)
	M_FG	640	786	–	**0.04** (0.01)	**0.29** (0.06)	**0.02** (0.01)	**0.31** (0.09)	**0.63** (0.05)	**0.01** (0)	**1.09** (0.08)
PH	M_G	16273	16306	0.69	**112.97** (7.64)	**70.52** (14.02)	**18.75** (6.5)	–	**128.80** (10.66)	**27.98** (2.29)	–
	M_FG	15481	15568	–	**47.07** (4.53)	**18.27** (12.47)	**24.51** (5.04)	–	**134.76** (11.13)	**28.07** (2.29)	–
GY	M_G	22552	22606	0.68	**0.48** (0.03)	**0.26** (0.05)	**0.17** (0.06)	**0.17** (0.05)	**0.50** (0.04)	**0.61** (0.05)	**0.47** (0.04)
	M_FG	21498	21644	–	**0.20** (0.02)	**0.11** (0.04)	**0.11** (0.05)	**0.06** (0.04)	**0.49** (0.04)	**0.61** (0.05)	**0.46** (0.03)
H_2_O	M_G	5870	5925	0.75	**0.96** (0.06)	**0.86** (0.09)	**0.20** (0.06)	**1.96** (0.12)	**0.56** (0.05)	**0.43** (0.03)	**0.36** (0.03)
	M_FG	4200	4346	–	**0.45** (0.03)	**0.53** (0.07)	**0** (−)	**0.64** (0.06)	**0.57** (0.05)	**0.39** (0.02)	**0.36** (0.03)
YI	M_G	22782	22836	0.68	**0.48** (0.03)	**0.26** (0.06)	**0.18** (0.06)	**0.22** (0.05)	**0.53** (0.04)	**0.66** (0.05)	**0.47** (0.04)
	M_FG	21765	21910	–	**0.19** (0.02)	**0.10** (0.05)	**0.11** (0.06)	**0.10** (0.04)	**0.53** (0.04)	**0.67** (0.05)	**0.47** (0.04)

AIC and BIC criterion are indicated for each model. H^2^ is the broad-sense multi-trial heritability estimated thanks to the model M_G. The estimations of variance components are indicated in bold and their standard errors are given in parenthesis

For all traits, AIC and BIC values (the smaller the better) showed that the inclusion of a recipient and family fixed effects were beneficial (model M_FG in Table 2). The error variances were similar between M_G and M_FG. The estimated genetic variances were lower with model M_FG than with model M_G, highlighting that including pedigree structure as fixed effects in model M_FG absorbed part of the genetic variation. The remaining within-family genetic variance was particularly low for ASI. For other traits, it represented between 40 and 67% of the global genetic variance. We also noticed a diminution of the G × E interaction variances when including family pedigree and corresponding interaction terms as fixed effects.

### Mean performance of hybrid families

Adjusted means of the reference hybrids and hybrid families were computed by environment and over environments with the model M_FG (Tables S2 and S3). We observed a strong variability of the phenotypic values of the reference hybrids across environments. The Smh19 trial, located in a warmer and drier climate than the two other trials, showed a faster flowering (55 DAP on average against 92 DAP in other trials) and an earlier maturity at harvest (mean H_2_O value of 23% in Smh19 against 28% in other trials). Hybrids were also taller in Smh19 (306 cm on average against 270 cm in other trials). For GY, the hybrids were less productive in Vil19, with a yield reduction of 2.20 Mg/ha on average compared to Smh19 and Blo19. We noticed systematic lower productivity of the reference hybrid corresponding to the recipient line A7 and its associated hybrid families (A7D3, A7D5, and A7D6).

**Fig. 2:**
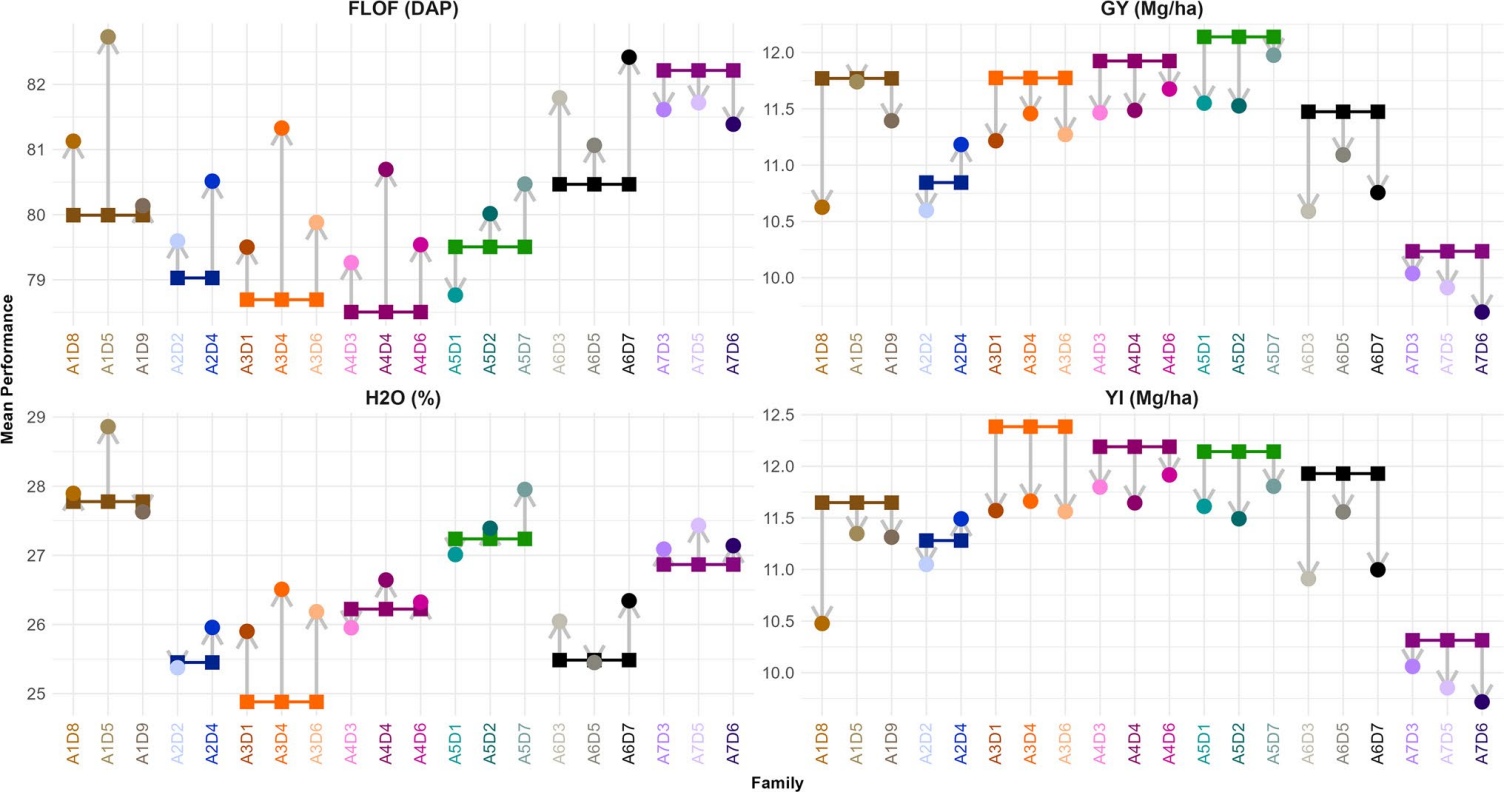
Mean performances of families and reference hybrids for FLOF, GY, H_2_O and YI**.** The dots indicate the family mean performances (colored by family) and the squares indicate the reference hybrid performances (family derived from the same recipient line are compared to the same reference). Mean performance of a family or a reference hybrid corresponds to its adjusted mean across the trials computed thanks to the estimated parameters of the model M_FG. Grey arrows highlight the difference between each family and its reference (color figure online)

For the flowering time (FLOF and FLOM), the average values of hybrid families were close to the reference hybrid value with a slight trend toward later flowering (one or two days, Fig. 2 and Table S4). This difference was significant for 12 families for FLOM and 18 families for FLOF. For all families, except A4D3 and A5D2, more than half of individuals had later male and female flowering time than the corresponding reference hybrid (Table S5). Half of the hybrid families did not show significant different H2O mean values compared to their reference hybrid (Table S4). For the other half, the difference ranged from 0.4 to 1.6%.

The donor introgressions came with a reduction of GY and YI for the different families (− 0.42 and − 0.53 Mg/ha on average, respectively). Only the A2D4 family had significantly higher average GY than its corresponding reference hybrid. Others displayed a large variation in performance loss: between 0.03 and 1.14 Mg/ha for GY and 0.3 and 1.17 Mg/ha for YI. We noticed that the loss of GY and YI compared to the reference hybrid was significant for the majority of families (13 families for GY and 16 families for YI). We distinguished different patterns of loss according to the recipient line. For example, the cross of A3 with three distinct donor lines led to hybrid families with similar average GY (A3D1, A3D4, and A3D6). In contrast, the hybrid families derived from A1 had contrasted mean productivities, with a difference of 1.10 Mg/ha between A1D8 and A1D9 for GY. Only one family (A6D7) had no individual with better YI performance than the corresponding reference hybrid. Other families displayed between 5 and 63% of individuals with YI adjusted mean values superior to that of the corresponding reference hybrid (Table S5).

### Within-family genetic variance comparison

**Fig. 3:**
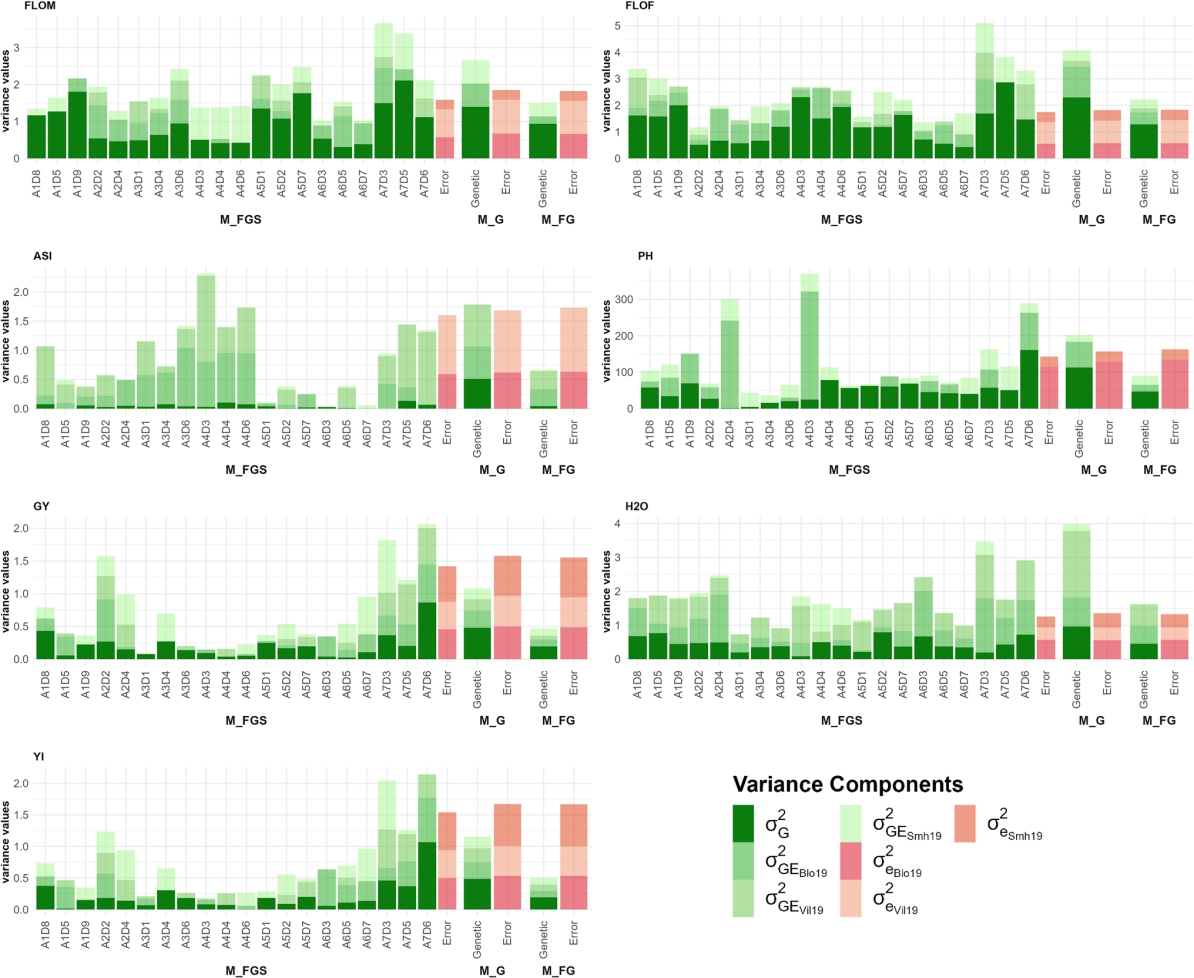
Variance decomposition with the model M_FG_S_ and within-family genetic variance estimation for each trait. For each family, genetic (in dark green) and G × E (in shades of light green) variances are showed. Error term variances are also displayed (in shades of pink). For comparison, variances estimated with the models M_G and M_FG are presented on the right part of each graph with the same color code (color figure online)

The model M_FG_S_ gave us access to the genetic and G×E variances associated with each family (Fig. 3 and Table S6). Likelihood ratio tests between M_FG and M_FG_S_ showed an advantage of considering specific genetic variances between families for all traits (Table S7). For all traits, the mean of within-family genetic variances estimated with M_FG_S_ was similar to the common genetic variance estimated with the model M_FG. The error variances were alike between both models (Tables 2 and S6). AIC values were lower with the model M_FG_S_. However, we noticed the BIC values were higher for this model.

For FLOM, the within-family genetic variances varied from 0.31 to 2.11. These values depended to a large extent on the recipient line used to generate the families (see contrast test procedure explained in File S4 and associated results of pairwise tests in Fig. S2). The families derived from the recipient lines A1, A5, and A7 had significantly higher genetic variances than other families. We also observed the influence of some donor lines on FLOM genetic variances. For example, the families derived from D4 presented significantly lower genetic variances than those derived from D1, D5, D7, D8 or D9. Compared to FLOM, genetic variance values were slightly higher for FLOF (from 0.43 to 2.86). The families derived from A1, A4, A5, A7 presented a higher FLOF variance than others. For ASI, all families had low genetic variances and high G×E variances, in agreement with the global variance decomposition provided by the model M_FG. We did not detect any systematic effect of parental lines on the genetic variance for this trait (Fig. S2).

The within family variances estimated with M_FG_S_ model were rather similar for GY and YI, with a Pearson coefficient correlation of 0.95. The ranges of genetic variances were 0.02–0.87 and 0.03–1.07 for GY and YI, respectively. For GY, we noticed significantly higher genetic variances for families derived from A1 and A7 than from A4 and A6 (Fig. S2). For YI, families whose recipient parent was A7 had significantly larger genetic variance values than all other families. We observed no systematic effect of donor lines on the within-family genetic variances. However, we observed variation between families sharing the same recipient line. For instance, for GY, A1D9 had a genetic variance four times higher than A1D5.

The range of the genetic variances was reduced for H_2_O (0.09–0.79). PH genetic variances were also relatively homogenous between families. We identified three families with extreme variance values: low for A2D4 (1.37) and A3D1 (4.85), and high A7D6 (160.99). A2D4 and A4D3 stood out from other families with a high G × E interaction. We observed no systematic effect of donor or recipient lines on variances for these traits.

### Within-family additive variance comparison

We obtained estimations of the within-family additive variances using the models M_FA_S_ and M_FA_S_P_S_, which included a family-specific permanent effect (Tables S8 and S9). For all traits, M_FA_S_ yielded lower AIC and BIC values than M_FA_S_P_S_. Both values were close for FLOM and FLOF. M_FA_S_P_S_, presented AIC values lower than M_FG_S_ for all traits but YI. Similarly to the genetic variances (see above), likelihood ratio tests between M_FA and M_FA_S,_ and between M_FAP and M_FA_S_P_S,_ showed that declaring family specific variances led to a better fit than considering a homogenous variance for the additive and permanent effect terms.

**Fig. 4:**
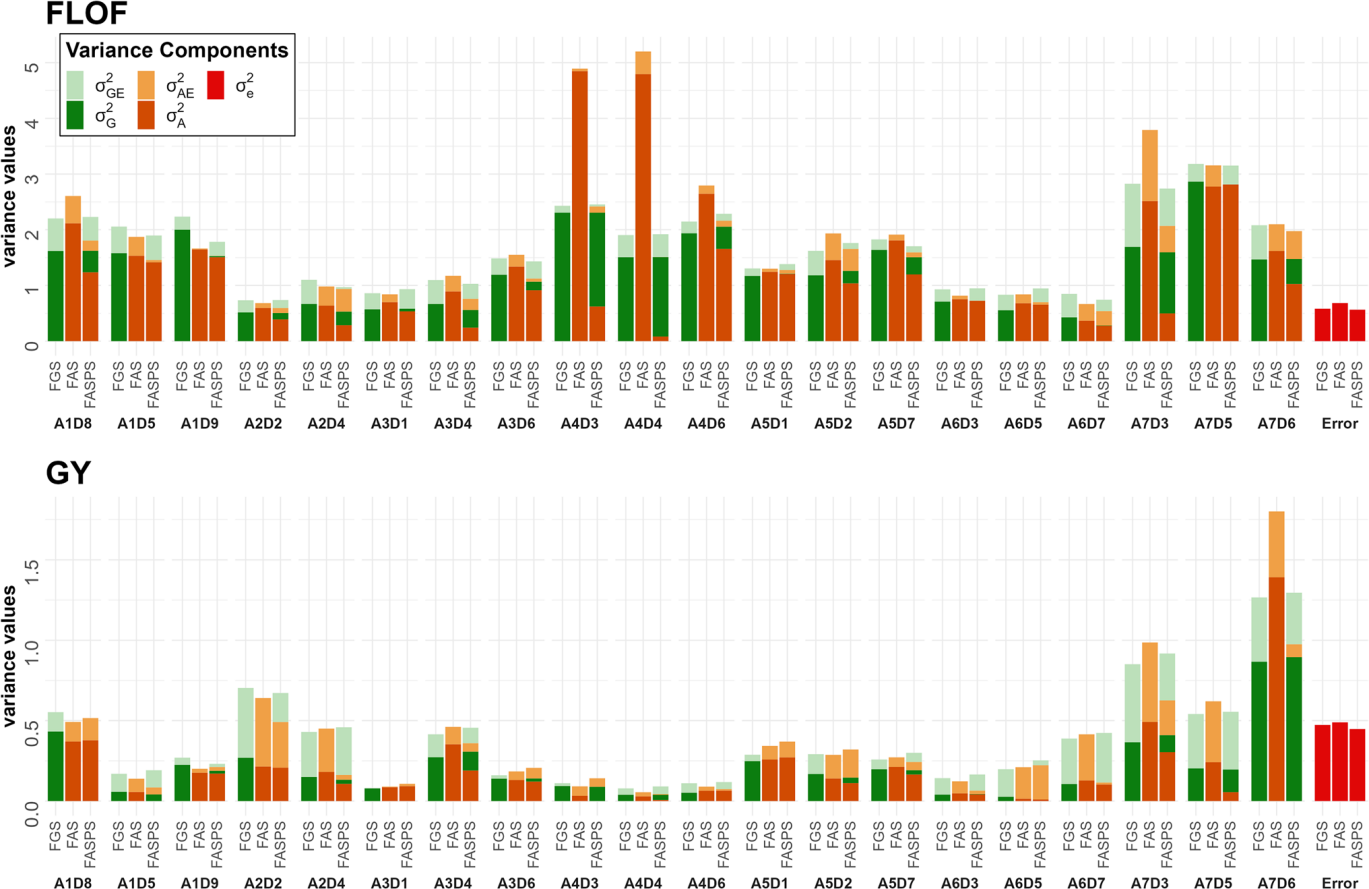
Within-family genetic and additive variance estimation with the models M_FG_S_, M_FA_S_ and M_FA_S_P_S_ for FLOF and GY. For M_FA_S_ and M_FA_S_P_S_, within-family additive variances are indicated in orange. The within-family genetic (model M_FG_S_) and permanent effect (model M_FA_S_P_S_) variances are in dark green. The interaction (A × E: light orange and G × E: light green) and error (red) terms are trial specific and represented by their respective average values (color figure online)

We noticed large variations in the ratio between the additive variances estimated with M_FA_S_ and the corresponding genetic variances estimated with M_FG_S_ across traits and families (Figs. 4, S3 and Table S10)_._ On average over families, this ratio ranged from 0.67 (ASI) to 1.28 (FLOF) excluding A7D3 for ASI which had close-to-zero genetic and additive variances. For FLOF and FLOM, some families such as A4D4 had additive variances which were up to three times their genetic variance values. Adding a family-specific permanent effect (model M_FA_S_P_S_) reduced the mean ratio between the additive variances and the M_FG_S_ genetic variances. For example, it dropped from 1.23 and 1.28 to 0.71 for FLOF and FLOM. This diminution appeared stronger for GY and YI, notably because of an estimation close to zero of the additive variance in some families.

The sum per family of the additive and permanent effect variances (model M_FA_S_P_S_) was close to the M_FG_S_ genetic variances for all traits except ASI (Table S10). For this trait, the permanent effect variances were estimated close to zero, and the average ratio of additive variances over genetic variances was 0.58 (excluding A7D3). The additive part of the genetic variances was higher for FLOM, FLOF, and H_2_O (from 0.73 to 0.83) than for PH, GY, and YI (from 0.6 to 0.7). For these six traits, this ratio varied strongly across families. For example, A1D8 and A7D3 had similar GY genetic variance values, but the additive part was higher for the first one (Fig. 4, Table S10). This variation was also observed between families derived from the same recipient.

### Family ranking based on the usefulness criterion

**Fig. 5:**
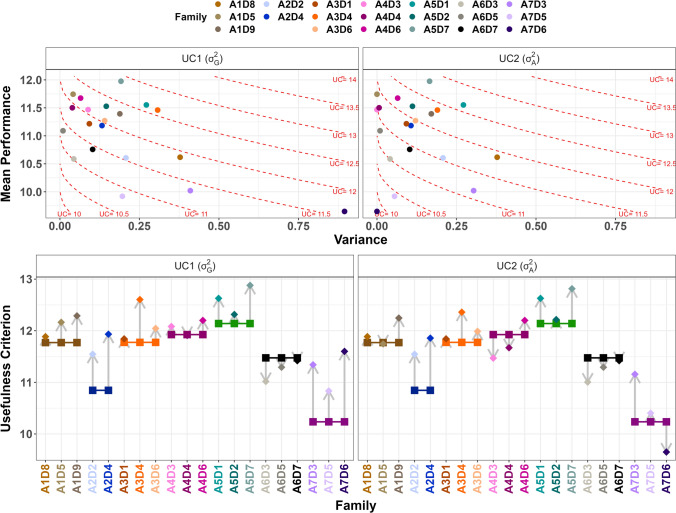
Usefulness Criterion (UC) prediction for each family and comparison with the reference hybrid performance for GY. The top charts present the relation between family mean performance and genetic variance components (left: within-family genetic variances, right: within-family additive variances). Dots are colored by family and dotted red lines are isoclines of UC. The bottom charts display the UC values for the different families (diamonds). For comparison, the performances of reference hybrids were added on the graphics (squares). Grey arrows visualize the potential gain for each family (color figure online)

The GY UC_1_, which estimates the expected mean of the top 5% selected individuals, was superior to the adjusted mean of the reference line for all families except for the families derived from A6 and A4D4 (Fig. 5). For YI, eleven families had UC_1_ values higher than the reference value. The average expected gain was 0.41 Mg/ha for GY and 0.29 Mg/ha for YI. These values rose to 0.54 and 0.55 Mg/ha if we considered only the families with higher UC_1_ values than the reference value. They corresponded to an expected mean gain of 5% for both traits. The maximal potential gain was observed for A7D6, which reached 13% for GY and 14% for YI. The family expected gains decreased as the reference line’s initial performance increased, with a correlation coefficient of − 0.72 for GY and − 0.89 for YI. We also noticed that the UC_1_ values across families were inferior to the corresponding best individual adjusted means, with a mean difference of 0.6 Mg/ha for GY and YI. Nevertheless, both quantities correlated well (0.78 for GY and 0.80 for YI).

For GY, the family rankings based on UC_1_ were identical to those based on mean performance for recipients A2, A3, and A5, whereas they differed for other recipients (Figs. 2 and 5, Table S11). For example, for A1, the family A1D9 had a lower mean performance than A1D5 but a higher genetic variance, leading to a superior UC_1_ value. We also noticed that A1D3 had a poor mean performance compared to A1D5 and A1D9 (10.6 Mg/ha against 11.7 and 11.4 Mg/ha) but a rather similar UC_1_ value. Using UC_1_allowed us to highlight the difference between A5D1 and A5D2 too, which had similar mean performances (1.15 Mg/ha) but contrasted genetic variance values (A5D1: 0.27 and A5D2: 0.15). For YI, the rankings established with UC_1_ and mean performance were similar for families derived from A2, A3, A5, and A6. Similarly to GY, using UC_1_ allowed us to underline families with high genetic variances, such as A1D9, which appeared superior to A1D8 and A1D5 (Table S11). Although the A2 reference hybrid had lower performance than the A1, A3, A4 and A5 reference hybrids (Fig. 1), A2D4 had similar GY and YI UC_1_ values than families issued from other recipients.

Considering only the additive part of the genetic variance to compute the usefulness criterion (UC_2_) led, by definition, to lower values. It also affected the within-recipient family ranking, although UC_1_ and UC_2_ correlated well (0.80 for GY and 0.73 for YI, Fig. 5; Table S11). The ranking differed for four recipients for GY and three for YI. For example, the families from A4 had similar GY UC_1_ values, but only A4D6 had a substantial additive variance, leading to a higher UC_2_. The higher the genetic variance and the lower the additive part was, the more the family was penalized with UC_2_ compared to UC_1_. The most extreme loss was observed on YI for A7D6 (UC_1_ = 11.8 Mg/ha and UC_2_ = 9.6 Mg/ha).

## Discussion

### Impact of the introduction of non-elite material on flowering time and yield performance

The selection of the donor lines was driven by a collective choice made by the seven partners of the project, which led to consider (i) originality relative to main founders of the flint genetic groups, (ii) hybrid performance, (iii) limited agronomic defaults, in particular lodging and (iv) phenology compatible with the targeted environment (File S1). Regarding this fourth factor, choice was restrained among those with the same precocity as the recipient line (B-C1 maturity groups according to French nomenclature) with the aim to produce progeny with phenology adapted to targeted environmental conditions. This pre-selection of the donor lines for the precocity was made possible by the assembly and evaluation of an European flint panel encompassing more than 1000 lines (Gouesnard et al. 2017). The incorporation of donors had a limited impact on flowering time average in progenies (a mean delay of one or two days) which will facilitate their use in the breeding programs of the private partners. This illustrates that characterization of genetic resources for adaptive traits such as flowering time to preselect donor lines is one of the keys to their efficient use in breeding programs.

Even though they passed a first selection step, diversity donors used in our study showed an average YI performance gap with elite lines of 2 Mg/ha (File S1). It was expected that their incorporation into elite material would result in a loss of performance (Pollak 2003; Allier et al. 2020a) but we also expected to identify transgressive individuals in the different families. Experimental evaluation of progenies in our study showed an average loss 0.57 Mg/ha excluding A2D4. This is consistent with the initial performance gap between donor and recipient lines and the performed cross type (BC1), which leads to anticipate 25% of the initial gap. For the A2D4 family, donor introduction led to a gain of 0.21 Mg/ha. This average gain was mainly due to a lower performance of the A2 reference hybrid in the Vil19 environment (Table S2). For the other families, the average performance loss was variable, ranging from 0.21 to 1.2 Mg/ha. Beyond the mean value, the progeny performances depend on the genetic variance generated by the cross. We noticed substantial YI within-family genetic variances. This is in accordance with several simulation studies which showed that crosses between parents with large performance differences may lead to high progeny variance (Mohammadi et al. 2015; Lado et al. 2017; Neyhart and Smith 2019). However, a large performance difference is not sufficient as illustrated by the presence of families with genetic variances close to zero. Wide differences in genetic variance were observed both between families derived from different recipients or the same one. Similar to our study, the experimental evaluation of a nested association mapping population of European flint maize identified a large range of genetic variances between half-sibling families sharing all a common parent (Bauer et al. 2013; Lehermeier et al. 2014). For most of the families, the level of variance in progenies was high enough to counterbalance the average performance loss and led to transgressive progenies with better performances than their recipient parent. The presence of transgressive individuals confirmed the interest of incorporating diversity donors in the flint maize elite material. Consistently with the observation of such transgressive individuals, the computation of the usefulness criterion showed an expected mean gain of 5% over the recipient parents after selecting the top 5% individuals within each family.

### Causes of genetic variance variations between families

We noticed a disparity in genetic variance values across families and traits. On average, the additive part of the genetic variance was higher for FLOF, FLOM and H_2_O than for other traits. The level of genetic variance in the progeny appeared to be more impacted by the recipient parent than by the donor parent. This trend was clear for FLOF and FLOM, for which the families from A1, A5 and A7 had a higher genetic variance. For yield-related traits, crossing donor lines with A1 and A7 led to higher genetic variances than other recipient lines. This preponderant influence of the recipient parent may first be linked to the backcrossing procedure. For some recipient lines, an involuntary selection in BC1 individuals during material creation might have led to the loss of some donor alleles (Ødegård et al. 2009; Neyhart and Smith 2019), causing a reduction of variance in progenies. However, the observed within-family marker-based diversity values are only slightly inferior to the theoretical ones which suggests a limited selection has been made during the material creation (Table S12).

The genetic variance differences between families may be linked to a difference in parental genetic distances: simulation work by Beckett et al. (2019) showed that the range of possible genetic variances increases as the genetic parent distance between parents becomes larger. The genetic distance between the pairs of donor and recipient used as parents in our design was slightly correlated to the FLOM within-family genetic variance (Pearson coefficient: 0.40, Fig. S4). For other traits, the genetic distance was poorly correlated to the genetic variance, which confirmed the results of other studies (Mohammadi et al. 2015; Beckett et al. 2019). This lack of correlation may be due to the genetic distance which is computed as a whole-genome relatedness based on neutral markers and not on the QTLs that contribute to the variance (Hung et al. 2012). One can expect that this relationship would be improved by accounting for QTL effects, as supported by results regarding heterosis prediction in wheat (Boeven et al. 2020).

The difference between within-family genetic variances may also be attributable to a characteristic of our experimental design: a specific tester line was used to evaluate the progenies of a given recipient line. This specificity allowed each private company to compare its new materials to its reference hybrid. In presence of dominance, using a tester that accumulates a high number of dominant favorable alleles leads to a reduction of the genetic variance in the hybrid population (Rawlings and Thompson 1962; Hallauer et al. 1988). This may explain why some families sharing the same recipient parent have comparable variances (e.g., low for A4 for GY). Differences in genetic variances may also be caused by donor specific epistatic interactions with the recipient alleles which may hide part of the new variations due to the incorporation of donor alleles. Complementary test crosses involving several testers may be necessary to test these hypotheses.

### Interest of the usefulness criterion to rank donor × recipient crosses

Zhong and Jannink (2007) highlighted that the interest of UC to compare bi-parental cross performances is restrained because the variation of mean performances of crosses is much higher than the variance of crosses genetic variances. In addition, the simulation work carried out by Beckett et al (2019) highlighted a strong correlation between the mean of parental performances and that of best progenies in a bi-parental cross. Our experimental results showed that crossing elite lines with diversity donors leads to families with variable genetic variances. In this context, using UC is necessary to consider this variation. In our study, the ranking of the crosses involving the same recipient line was largely reshaped when it was based on the usefulness criterion instead of the GY or YI mean performances. Using the UC_1_ allowed us to identify the most interesting crosses to generate transgressive progenies for each private company (e.g., A2D4 for A2, A3D4 for A3, A5D7 for A5). Note that UC has also been recommended for the selection of crosses within a selection program and was proved useful to increase the genetic gain (Lehermeier et al. 2017; Yao et al. 2018; Allier et al. 2020a).

We also proposed a ranking based on UC_2_. This indicator, which considers the additive variance rather than the genetic variance, enables one to project the future response to selection that could be achieved in the progeny of the best individuals of each family. The computation of UC_2_required to estimate accurately the GY and YI within-family additive variances. We estimated these variances without (M_FA_S_) or with (M_FA_S_P_S_) a permanent effect in the model, which represented the non-additive genetic effects such as epistasis effect (Kruuk 2004; Vitezica et al. 2018). Adding this effect to the model increased the AIC and BIC. This could be due to the low proportion of repeated hybrids in our experiment, preventing accurate estimations of variances linked to the permanent effect. Nevertheless, the comparison of both models revealed the importance to consider such permanent genetic effect, to avoid the overestimation of the additive variances (as also observed by González-Diéguez et al. 2021). The UC_2_ appeared as a good tool to distinguish between families having similar UC_1_ values and privilege those with higher additive variances (e.g., A1D9 rather than A1D5 for the A1 recipient).

More individuals derived from the most promising crosses could be created to maximize the opportunity to find progenies with high performance. The choice of the number of new individuals may be specific to each company and may be guided using the expected maximum breeding value (EMBV, Müller et al. 2018). This indicator gives the expected performance of the best individual for a DH population of a given size estimating their breeding values.

### Implementation in breeding programs and future work

**Fig. 6:**
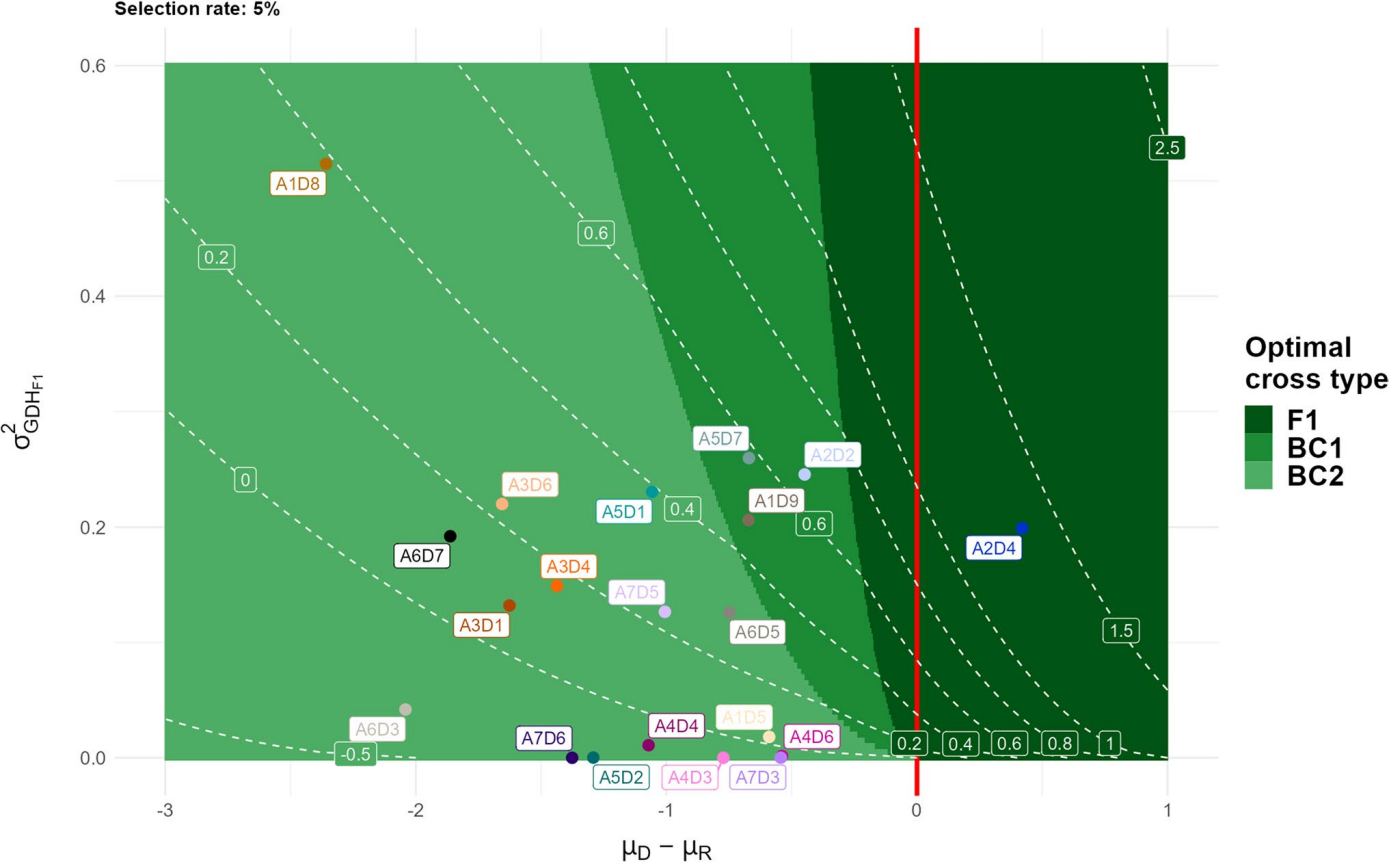
D × R cross type choice to maximizing the expected gain after selection. ${\mu }_{\mathrm{D}}$ and ${\mu }_{\mathrm{R}}$ are the performance values of the donor and recipient parents. ${{\sigma }_{\mathrm{G}}^{2}}_{{\mathrm{DH}}_{\mathrm{F}1}}$ is the theoritical genetic variance in a DH population derived from F1 cross between both parents. For pairs of ${{\sigma }_{\mathrm{G}}^{2}}_{{\mathrm{DH}}_{\mathrm{F}1}}$ and ${\mu }_{\mathrm{D}}-{\mu }_{\mathrm{R}}$ values, we compared three cross types (F1, BC1 and BC2) computing the usefulness criteria in DH populations derived from each. For the backrosses, the recurrent parent was the recipient line. The usefulness criteria were computed for a selection rate of 5%. For each parameter pair, the cross type which maximized the performance gain in comparison with the recipient line was considered as optimal. This is visualised through the green areas. The white dashed lines give the expected gain after selection in the DH population derived from the optimal cross. The red line indicates the case were ${\mu }_{\mathrm{D}}$ and ${\mu }_{\mathrm{R}}$ are equal. The experimental evaluated crosses were positionned in the graph using their mean performance and genetic variance values (color figure online)

The creation and evaluation of a large multi-parental population confirmed experimentally the interest of introducing genetic resources into elite material, therefore supporting recent simulation results (Allier et al. 2020a; Vanavermaete et al. 2021; Sanchez et al. 2023). Our mating design can be viewed as a cooperative bridging population fulfilling a dual task: identifying promising D×R crosses and delivering performing new lines directly usable as flint parents in breeding programs. To this aim it was required to deal with two constraints: (i) incorporating a large enough proportion of donor genome in progeny to explore new variations and (ii) minimizing the loss in the global performance due to the lower donor performance. Backcrossing the D×R crosses with the recipient parents turned out to be a good compromise as it generated, for most populations, transgressive individuals superior to the recipient elite line. One may however wonder whether a lower or higher proportion of donor genome would have been preferable. We addressed this question under a simple genetic model aiming at extrapolating our results to other possible pedigrees for the same donor x recipient combinations, as described in appendix File S5. This comparison of F1, BC1 and BC2 cross types indicates that the maximal UC values for the observed crosses in this study should be reached in general with two backcrosses with the recipient parent (BC2), as the decrease of the variance in the progeny is counterbalanced by the mean performance gain (Fig. 6, File S5). Note however, that if such populations are considered as a bridging step before introduction into an elite pool, the next breeding generations should also be considered. Simulation work showed that progenies of D × R crosses selected for introduction into the elite program can be preserved and improved in the elite breeding program provided selection is performed under a diversity constraint (Allier et al. 2020a; Sanchez et al. 2023). Such genetic resources generally contribute to varietal release after three crosses with the elite material in total (Sanchez et al. 2023). F1 and BC1 populations that may be sub-optimal compared to BC2 in terms of UC may nevertheless be a good option for the bridging step as individuals will carry more introduced segments.

Finally, our results highlight large differences in UC across donor recipient pairs. This supports experimentally the conclusion of Sanchez et al. (2023) that bridging is a key step to select donors prone to improving a given elite pool. Despite its large size, our design permitted to characterize only a restrained number of D × R crosses and its deployment to a larger scale could be expensive. Pre-selection of promising D × R crosses therefore would be of key interest before producing and evaluating them. Genomic selection approaches have been considered to predict usefulness criterion for such a pre-selection (Civan et al. 2021). The prediction of genetic variance for a given cross remains challenging as methods developed so far demonstrated inconsistent accuracies (Tiede et al. 2015; Adeyemo and Bernardo 2019; Neyhart and Smith 2019). Nevertheless, the use of our interconnected multi-parental populations to calibrate such prediction models would deserve evaluation.

## Conclusion

Altogether, our results support the usefulness of incorporating genetic resources into elite flint maize. Given the genetic gap between genetic resources and elite material observed at the beginning of this project (approx. 2 Mg/ha), we estimated that donors should be crossed three times to elite materials to maximize the expected value of selected progenies. We observed contrasted genetic variances and components (additive vs. permanent) across crosses, which can lead to large differences in both short-term and longer-term selection potential. This confirms the role of bridging programs as ours to detect the most suitable donor × elite pairs. In our experiment, only 20 such crosses could be evaluated despite a large size design of 1174 hybrids. An interesting prospect would be to evaluate the potential of genomic prediction based methods (Allier et al. 2020b) to predict the variance of additional crosses and identify the most promising ones. Finally, our results support further effort to create fixed diversity donors in European flint maize (Böhm et al. 2017; Mayer et al. 2020) and evaluate them to conduct an efficient preselection step, which appears key in simulation work (Sanchez et al. 2023).

## Author contributions statement

CB, LM, AM and AC initiated this project. CB and AC coordinated it with the help of SM, LB and AM. CB, CP and BL contributed to the development of the plant material. DM and VC provided the genotyping data. AA analyzed the results to determine the crossing plan. DS analyzed the results and prepared the manuscript. AC, LM, TMH and SBS supervised this work. All authors revised and approved the manuscript.

### Supplementary Information

Below is the link to the electronic supplementary material.

Supplementary file1 (DOCX 1723 KB)

## Data Availability

The phenotypic datasets generated and analyzed during the current study are available from the corresponding author on reasonable request.
