# Supplementary material for: Assessing the potential of genetic resource introduction into elite germplasm: a collaborative multiparental population for flint maize
Source: Theor Appl Genet. 2024 Jan 12;137(1):19. doi: 10.1007/s00122-023-04509-5 (PMC10786986; doi:10.1007/s00122-023-04509-5)
Supplement: Supplementary file 1 — Supplementary file1 (DOCX 1723 KB) [file 122_2023_4509_MOESM1_ESM.docx]

**Supplementary materials for:**

**Assessing the potential of genetic resource introduction into elite germplasm: a collaborative multiparental population for flint maize**

Dimitri Sanchez^1^, Antoine Allier^1,2^, Sarah Ben Sadoun^1^, Tristan Mary-Huard^1,3^, Cyril Bauland^1^, Carine Palaffre^4^, Bernard Lagardère^4^, Delphine Madur^1^, Valérie Combes^1^, Stéphane Melkior^5^, Laurent Bettinger^6^, Alain Murigneux^7^, Laurence Moreau^1^, Alain Charcosset^1,*^

^1^ Université Paris-Saclay, INRAE, CNRS, AgroParisTech, Génétique Quantitative et Evolution – Le Moulon, Gif-sur-Yvette, 91190, France

^2^ Syngenta, 12 Chemin de l’Hobit, Saint-Sauveur, 31790, France (current adress)

^3^ MIA, INRAE, AgroParisTech, 22 place de l’Agronomie, Palaiseau, 91120, France

^4^ UE 0394 SMH, INRAE, 2297 Route de l’INRA, Saint-Martin-de-Hinx, 40390, France

^5^ RAGT2n, Druelle, 12510, France

^6^ LIDEA FRANCE, Avenue Gaston Phoebus, Lescar, 64230, France

^7^ Limagrain Europe, 28 route d’Ennezat, Chappes, 63720, France

^*^Corresponding author: alain.charcosset@inrae.fr

**File S1. Donor line selections**

**Genetic material**

Each partner provided the parents of an elite hybrid. For each elite hybrid, the flint parent was considered as the recipient line (noted A) and the dent panel as the tester line (noted B). We pre-selected 74 candidate donor lines (noted D) from a collection of 1,191 lines representative of flint diversity (Gouesnard et al. 2017). Constraint at this step were to avoid strong similarity with key founders of the Flint group (i.e. F2, F7, Ep1, D105) and to belong to the same precocity group as the elite recipient lines (B-C1 maturity groups according to French nomenclature). These lines consisted of (i) flint lines from the INRAE genetic resources collection, managed by the station of Saint Martin de Hinx, (ii) DH-SSD lines derived from flint landraces in the ProMaïs DIVZEA project, (iii) SSD lines derived from flint landraces evaluated as drought tolerant in the RESGEN European program and (iv) flint lines from the panel assembled for the CornFed project and evaluated for forage(Rincent et al. 2012).

**Molecular markers**

The hybrid parents and the candidate donor lines were genotyped using the Maize Illumina Infinium 50K array (Ganal et al. 2011). In the following, we only considered the 32,788 Panzea SNP markers that have been shown to prevent ascertainment bias. We obtained a set of 26,657 SNP markers after removing the monomorphic markers and filtering those with a call rate inferior to 0.9.

**Phenotypic data**

Each partner, except partner 7 (INRAE), produced and evaluated the hybrids between the candidate donor lines and its tester (D x B) as well as between its recipient and tester (A x B). Hybrids from one partner were evaluated in its European field network for grain moisture (H2O, %) and grain yield (GY, Mg/ha) in the summer of 2016. In total across partners, 26 locations were involved. In addition to GY and H2O, plant stalk lodging was recorded by 5 partners in a total of 12 locations. This phenotyping strategy was chosen to facilitate the trial implementation by the different private partners in their standard conditions, although it induces confusion between the tester effect and other partner effects (e.g. seed quality,harvest protocol , …). Note that the D x B hybrids were not repeated in a given location (except for partner 2) and the number of actually evaluated D x B hybrids was variable according to partners and locations (from 28 to 68). In absence of randomized check lines, no spatial heterogeneity correction was performed. Across locations, 43 outlier plots were identified and removed for GY and H2O.

**Flint line value estimations and yield index computation**

For a given partner, the H2O flint line LS-means were computed using the following model:

$$Y_{il}=\mu+{\alpha_{i}+ L_{l}+eE}_{il} (1)$$

$$L\sim N\left( 0,{I\sigma}_{L}^{2} \right), E\sim N\left( 0,{I\sigma}_{eE}^{2} \right)$$

$$L\perp eE$$

where,$Y_{il}$ is the phenotypic value of the hybrid between the tester of the partner and the flint parent $i$evaluated in location $l$. $\mu$ is the intercept term, $\alpha_{i}$ is the fixed effect of the flint parent (recipient line or candidate donor lines). $L_{l}$ is the random effect of location. ${eE}_{il}$ is the error term.

For each partner $k$, the Yield Index (YI) was computed for D x B hybrids in each location $l$ using the H20 elite flint line LS-means from $(1)$ :

$$YI_{\left( D_{j}*B_{k} \right)_{l}}=GY_{\left( D_{j}*B_{k} \right)_{l}}-2.5*\left( {H2O}_{\left( D_{j}*B_{k} \right)_{l}}-{H2O}_{A_{k}*B_{k}} \right) (2)$$

where $D_{j}$ is the $j^{th}$candidate donor line, $A_{k}$ and $B_{k}$ are the recipient and tester lines of the partner $k$.

**Decomposition of hybrid values and donor value estimations**

The YI DxB hybrid values were decomposed as follows:

$$Y_{jkl}=\mu+{\beta_{j}+\gamma_{k}+ L_{kl}+e}_{jkl} (3)$$

$$L_{k}\sim N\left( 0,{I\sigma}_{L_{k}}^{2} \right), e\sim N\left( 0,{I\sigma}_{e}^{2} \right)$$

$$L_{k}\perp e$$

where $Y_{jkl}$ is the phenotype of the hybrid between the donor $j$ and the tester $k$ evaluated of the location $l$. $\mu$ is the intercept term. $\beta_{j}$ is the fixed effect of the donor, $\gamma_{k}$ is the fixed effect of the tester (confounded with a meta-environment effect). $L_{kl}$ is the random effect of the location nested in the tester effect. $e_{jkl}$ is the error term.

The donor effect LS-means were used to estimate the General Combining Abilities (GCA) of candidate donor lines. LS-means allowed to consider differences in the phenotyping effort made by partners: more weight was given to partners with more locations.

**Plant lodging evaluation**

Stalk lodging has been observed during the trials in some locations mostly in the west of France. To avoid selection pressure due to stalk lodging sensitivity during the next steps of the project, we decided to eliminate candidate lines that had high lodging levels. Since all testers were assumed to bring a roughly similar level of tolerance for plant lodging, we decided to realize a meta-analysis of all recorded scores independently of the tester. Lodging scores from 1 (high sensitivity) to 9 (low sensitivity) were transformed into proportions of lodged plants.

The candidate donor lines LS-means were computed using the following model:

$$Y_{jkl}=\mu+{\beta_{j}+ L_{kl}+e}_{jkl} (4)$$

$$L_{k}\sim N\left( 0,{I\sigma}_{L_{k}}^{2} \right), e\sim N\left( 0,{I\sigma}_{e}^{2} \right)$$

$$L_{k}\perp e$$

where $Y_{jkl}$ is the phenotype of the hybrid between the donor $j$ and the tester $k$ evaluated of the location $l$. $\mu$ is the intercept term. $\beta_{j}$ is the fixed effect of the donor. $L_{kl}$ is the random effect of the location nested in the tester effect. $e_{jkl}$ is the error term.

Parameter estimations of all models were estimated thanks to ASREML-R v4 (Butler et al. 2009).

**Selection of candidate donors**

18 candidate lines, issued from five selfing generations, were discarded because of their high residual heterozygote rate (> 10%).


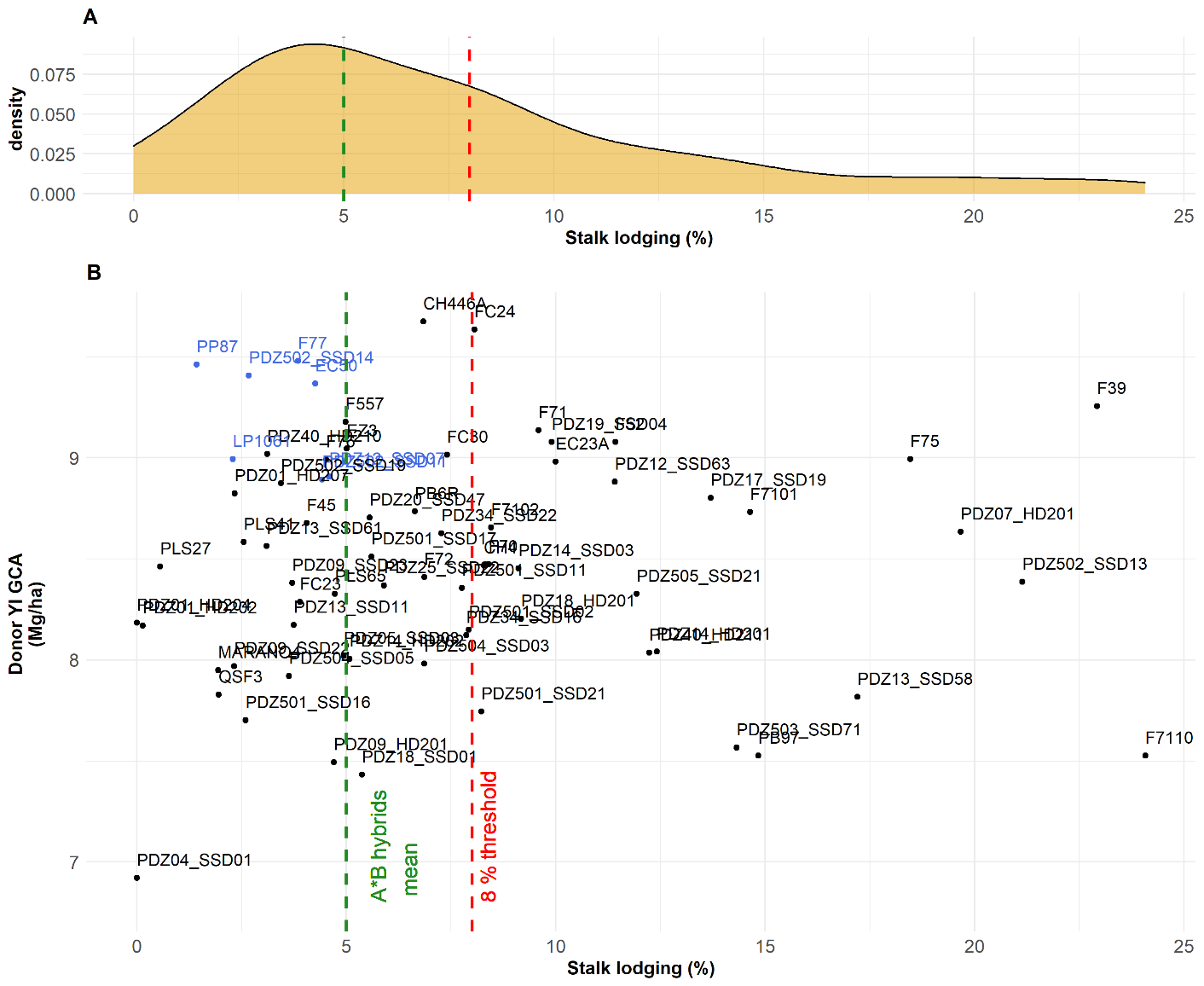
From the remaining candidates, those presenting more than 8% of lodged plants were eliminated. This threshold was determined using the mean lodging value of the elite A x B hybrids and the global distribution (**Fig. 1**). The candidates with higher YI GCAs were considered to be donors. Additionally, the availability in nursery of candidate D x R BC1 crosses and partners’ wishes were also taken into account. Partners’ wishes were considered as expert information based on the knowledge of their material (ear insertion height, sensibility to disease …) and their observations of the D x B hybrids (discriminant lodging score in their trials …). The selection procedure led to a set of 7 donor lines with diverse origins which presented an average gap performance of -1.98 Mg/ha with the elite hybrids (**Table 1**).

**Fig. 1 Density plot of stalk lodging LS-means (A) and scatter plot between YI GCA and stalk lodging LS-means of candidate lines (B)** The selected donors are indicated in blue.

**Table 1 Set of selected donor lines**

| **Name** | **Pedigree** | **YI GCA (Mg/ha)** | **Stalk lodging (%)** |
| --- | --- | --- | --- |
| F77 | Pop. Liesse-3.1.2 (PPS 19) | 9.48 (-1.72) | 4 |
| PP87 | Pop. Isola Basso | 9.46 (-1.74) | 1 |
| PDZ502_SSD14 | Aranga 1_14-01m-4m-1m-m | 9.41 (-1.79) | 3 |
| EC50 | Composite Aranga x EC18 | 9.37 (-1.83) | 4 |
| LP1061 | Compuesto Colorado Precoz | 8.99 (-2.21) | 2 |
| PDZ12_SSD07 | VA5 Nostrano dell Isola_07-01m-5m-1m-1 | 8.91 (-2.29) | 5 |
| PDZ502_SSD11 | Aranga 1_11-01m-2m-1m-m | 8.89 (-2.31) | 4 |

For YI GCA, the average performance gap between the donor line and the elite recipient lines is indicated in parenthesis.

**File S2** Parameters for the imputation of missing genotypic data

The imputation of missing genotypic data was performed using AlphaPlantImpute v 1.1(Gonen et al. 2018) with the following parameter:

AllSameDensity: 1

PhaseUsingMap: 0

GenotypedSnpThreshold: 0.95

SnpDistanceFillThreshold: 0.01

CalculateAccuracy: 0

FillMissingParentAverage: 1

ImputeParentsAndStop: 0

PedigreeErrorGenoThreshProp: 0.05

ReportPedigreeErrorAndStop: 0

CheckPedErrorParentHaplo: 0

**File S3. Spatial correction of raw phenotypic data**

**Modelling of spatial heterogeneity**

In each trial, different models were fitted account for spatial heterogeneity in raw phenotypic data. A general model can be described as:

$$Y_{ircbjkhm}^{R}= \mu+ \eta_{i}+ \rho_{j}+\theta_{k}+ G_{h}+S_{rcb}+ e_{ircbjkhm}$$

where $Y_{ircpjkhm}^{R}$ is the raw phenotype value of the repetition $m$of the hybrid $h$ derived from the recipient line $j$ belonging to the family $k$ located in the row $r$ and the column$c$ in the trial and belonging to the bloc $b$ in the sub-trial $i$. $\eta_{i}$ is the fixed effect of the sub-trial $i$, $\rho_{j}$ is the fixed effect of the recipient line (a factor with 8 levels: one level for each recipient line and an additional level for ADEVEY) and $\theta_{k}$ is the fixed effect of the family (a factor with 21 levels: one level for each hybrid family and an additional level for ADEVEY and the referent hybrids). $G\sim N\left( 0,{I\sigma}_{G}^{2} \right) iid$ is a random genotypic effect and $e\sim N\left( 0,{I\sigma}_{e}^{2} \right) iid$ is an error term. The independence is assumed between random terms. $S$ is a random spatial effect, which differs between spatial models, as described in the table 1 below.

The random spatial effect was decomposed into row and column ($R_{r}+ C_{c}$) effects or a bloc effect ($B_{b}$). These effects are considered over the whole trial or per sub-trial. If they were declared per sub-trial, the associated variances were defined specific to each sub-trial or common between sub-trials. An AR1xAR1 model considering the rows and the columns was also fitted. (**Table 1**)

**Table 1**. Description of random spatial terms for the different spatial correction models

| **Spatial**  **Correction Type** | **Approach** | **Spatial Effect Definition** |
| --- | --- | --- |
| Row x Colum | Whole trial | $\boldsymbol{R}_{\boldsymbol{r}}+ \boldsymbol{C}_{\boldsymbol{c}}$  $R\sim N\left( 0,{I\sigma}_{R}^{2} \right), C\sim N\left( 0,{I\sigma}_{C}^{2} \right)$ |
|  | Per sub-trial with common variances | $\boldsymbol{R}_{\boldsymbol{ri}}+ \boldsymbol{C}_{\boldsymbol{ci}}$  $R_{i}\sim N\left( 0,{I\sigma}_{R}^{2} \right), C_{i}\sim N\left( 0,{I\sigma}_{C}^{2} \right)$ |
|  | Per sub-trial with specific variances | $R_{ri}+ C_{ci}$  $R_{i}\sim N\left( 0,{I\sigma}_{R_{i}}^{2} \right), C_{i}\sim N\left( 0,{I\sigma}_{C_{i}}^{2} \right)$ |
| Bloc | Whole trial | $\boldsymbol{B}_{\boldsymbol{b}}$  $B\sim N\left( 0,{I\sigma}_{B}^{2} \right)$ |
|  | Per sub-trial with common variances | $\boldsymbol{B}_{\boldsymbol{bi}}$  $B_{i}\sim N\left( 0,{I\sigma}_{B}^{2} \right)$ |
|  | Per sub-trial with specific variances | $\boldsymbol{B}_{\boldsymbol{bi}}$  $B_{i}\sim N\left( 0,{I\sigma}_{Bi}^{2} \right)$ |
| AR1 x AR1 | Whole trial | $\boldsymbol{S}_{\boldsymbol{hijkm}}$  $S\sim N\left( 0,{\Sigma_{c}\left( \omega_{c} \right)\otimes\Sigma_{r}\left( \omega_{r} \right)\sigma}_{S}^{2} \right) ind$ |

$R$ is a row effect, $C$ is a column effect and $B$ is a bloc effect. $\sigma_{R}^{2}$ is the variance associated to the rows, $\sigma_{C}^{2}$ is the variance associated to the columns, $\sigma_{B}^{2}$ is the variance associated to the blocs. $\sigma_{R_{i}}^{2}$and $\sigma_{C_{i}}^{2}$ are specific to the sub-trial $i$. Used indices have the same meaning than in the general model. $\Sigma_{c}$ and $\Sigma_{r}$ are the AR1 covariance matrices associated to rows and columns, respectively, with $\omega_{c}$ and $\omega_{r}$their associated auto-correlation parameters (Gilmour et al 1997; Cullis et al 1998). $\otimes$ refers to Kronecker product.

For all traits, AIC had the lowest values with the AR1xAR1 model. This spatial correction type was retained.

**Modelling of genotypic effects and computation of corrected data**

To improve the estimation precision of spatial effects, genotypic information was included in the data correction model by the use of a kinship matrix $K$ computing following the recommendation given by (Vitezica et al. 2017). To this end, the following model was fitted:

$$Y_{ijkhm}^{R}= \mu+ \eta_{i}+ \rho_{j}+\theta_{k}+ A_{h} +G_{h}+S_{hijkm}+ e_{ijkhm}$$

$$A\sim N\left( 0,{K_{tot}\sigma}_{A}^{2} \right) , G\sim N\left( 0,{I\sigma}_{G}^{2} \right) iid, S\sim N\left( 0,{\Sigma_{c}\left( \omega_{c} \right)\otimes\Sigma_{r}\left( \omega_{r} \right)\sigma}_{S}^{2} \right), e\sim N\left( 0,{I\sigma}_{e}^{2} \right) iid$$

$$A\perp G\perp S\perp e$$

where $Y_{ijkhm}^{R}$ is the raw performance of the repetition $m$of the hybrid $h$ derived from the recipient line $j$ belonging to the family $k$ located in the sub-trial $i$. The fixed terms are the same than the terms of the general model. $A_{h}$ is a random additive genetic effect, $G_{h}$ is a random permanent genetic effect, $S_{hijkm}$ is a random spatial field effect and $e_{hijkm}$ is the error.

This model was called *AR1xAR1_A_G*. It was compared to two sub-models: *AR1xAR1_A* (the permanent genetic effect was removed) and *AR1xAR1_G* (the genotypic information was not used to model the genetic effect). For each trial, the best linear unbiased predictors (BLUPs) of the genetic effects were calculated. These BLUPs were added to the estimated mean family performance to obtain genetic values of the individuals. These genetic values were correlated to the average raw values in other trials. These correlations were used as a precision indicator to select the most pertinent correction model for each trial (**Table 2**). The corrected field plot values were obtained by subtracting spatial BLUPs of the selected model from the raw values.

**Table 2.** Correlation between genetic values and raw phenotypic values between trials

| **Trait** | **Model** | Blo19 | | Vil19 | | Smh19 | |
| --- | --- | --- | --- | --- | --- | --- | --- |
|  |  | Vil19 | Smh19 | Blo19 | Smh19 | Blo19 | Vil19 |
| FLOM | AR1xAR1_G | **0.597** | **0.643** | 0.670 | 0.574 | 0.591 | 0.551 |
|  | AR1xAR1_A | 0.597 | 0.642 | **0.656** | **0.593** | **0.600** | **0.558** |
|  | AR1xAR1_A_G | 0.567 | 0.616 | 0.656 | 0.593 | 0.537 | 0.493 |
| FLOF | AR1xAR1_G | **0.651** | **0.682** | **0.621** | **0.734** | 0.611 | 0.697 |
|  | AR1xAR1_A | 0.639 | 0.676 | 0.617 | 0.737 | **0.609** | **0.707** |
|  | AR1xAR1_A_G | 0.619 | 0.653 | 0.600 | 0.725 | 0.575 | 0.663 |
| ASI | AR1xAR1_G | 0.555 | 0.546 | 0.554 | 0.554 | 0.561 | 0.556 |
|  | AR1xAR1_A | **0.561** | **0.547** | **0.559** | **0.561** | **0.570** | **0.570** |
|  | AR1xAR1_A_G | 0.542 | 0.539 | 0.555 | 0.557 | 0.558 | 0.550 |
| PH | AR1xAR1_G | - | 0.611 | - | - | 0.455 | - |
|  | AR1xAR1_A | - | **0.615** | - | - | **0.479** | - |
|  | AR1xAR1_A_G | - | 0.600 | - | - | 0.440 | - |
| GY | AR1xAR1_G | **0.500** | **0.336** | 0.400 | 0.303 | 0.282 | 0.377 |
|  | AR1xAR1_A | 0.478 | 0.321 | **0.402** | **0.308** | **0.285** | **0.389** |
|  | AR1xAR1_A_G | 0.487 | 0.312 | 0.392 | 0.301 | 0.277 | 0.389 |
| H2O | AR1xAR1_G | 0.573 | 0.368 | 0.439 | 0.365 | 0.488 | 0.558 |
|  | AR1xAR1_A | **0.599** | **0.413** | **0.436** | **0.371** | **0.510** | **0.577** |
|  | AR1xAR1_A_G | 0.597 | 0.401 | 0.415 | 0.361 | 0.485 | 0.565 |

For each model (AR1xAR1_G, AR1xAR1_A and AR1xAR1_A_G), the estimated genetic values in a trial (first row) was correlated with the raw phenotypic data in other trials (second raw). Bold values indicate which model was selected for each trial-trait pair.

**File S4** Definition of the test of contrasts

**Comparison of the mean value of a family and the value of its recipient parent**

We named $\hat{\theta}$ the vector containing the estimations of the fixed parameters of the model M_FG_S_. To test the difference between the multi-trial mean value of a family F and the value of its recipient parent, we defined the contrast $C\hat{\theta}$ where $C$ is a vector containing the following coefficients:

$$\left\{ {{1 for the parameter \theta_{TEM} \atop\frac{1}{3} for each parameter \left( \alpha\theta\right)_{iTEM} \mathrm{with} i \in\left\{ Blo19, Smh19, Vil19 \right\}} \atop{-1 for the parameter \theta_{F} \atop-\frac{1}{3} for each parameter \left( \alpha\theta\right)_{iF} \mathrm{with} i \in\left\{ Blo19, Smh19, Vil19 \right\}}} \right.$$

As a reminder, TEM is an additional level of the family effect sharing by the commercial check (ADEVEY) and each recipient line.

For each family, the nullity of the contrast was tested thanks to a chi-squared test. A Benjamini-Hochberg multiple testing correction to control the FDR level at a nominal level of 0.05. The significance of these tests has been reported in the **Table S3.**

**Pairwise comparison of recipient or donor lines for the genetic variance in progeny**

We named $\hat{\theta'}$ the vector containing the estimations of the within family genetic variances given by the model M_FG_S_. We considered two recipient lines ($i$ and $j$). We named $n_{i}$ (resp. $n_{j}$) the number of families derived from the recipient line $i$ (resp. $j$). We defined the contrast $C'\hat{\theta}'$where $C'$ is a vector containing the following coefficients:

$$\left\{ \begin{aligned} \frac{1}{n_{i}} for variance parameters associated to families derived from i \\ -\frac{1}{n_{j}} for variance parameters associated to families derived from j \\ 0 for other families \end{aligned} \right.$$

The nullity of the contrast was tested thanks to a chi-squared test. A Benjamini-Hochberg multiple testing correction to control the FDR level at a nominal level of 0.05. A similar approach was conducted to test the influence of the donor line used on the within family genetic variances. The significance of these tests are presented in the **Fig S2**.

**File S5** Comparison of the expected gain after selection for F1, BC1 and BC2 cross types

In the following document, we consider a cross between a donor line (D) and a recipient line (R) evaluated for an additive quantitative trait. We detail the expression of the expected gain after selection, considering R performance as a reference, for different populations derived from the cross. This gain can be express as:

$$Gain_{p}=UC_{p}- \mu_{R} (1)$$

where $\mu_{R}$ is the performance of the recipient line and $UC_{p}$ is the usefulness criterion of the population $p$. For a given population $p$, the UC is computed using its mean performance ($\mu_{p}$) and its genetic standard deviation ($\sigma_{G_{p}}$):

$$UC_{p}=\mu_{p}+ih\sigma_{G_{p}} (2)$$

where $i$ is the selection intensity, $h$ is the selection accuracy. For sake of simplicity, we consider that $h=1$. The $\mathrm{UC}$ values will be calculated with $i=2.07$ (selection rate of 5%).

(1) can be also be written as:

$$Gain_{p}=Gap_{p}+i\sigma_{G_{p}} (3)$$

where $Gap_{p}= \mu_{p}- \mu_{R}$

In a first part, we will explicit the expression of $\mu_{p}$ and $\sigma_{G_{p}}$ for F1, BC1 or BC2 populations derived from the same DxR cross. In a second part, we will determine which population structure leads to maximize the UC for a range of parental performance gaps and genetic variances.

1. **Expression of mean performance and genetic variance**

## Mean performance

We consider a population of individuals derived from a DxR cross. We note $\mu_{D}$ and $\mu_{R}$ the respective performance of the donor and recipient line. We consider that the mean performance of a population derived from this cross will be only influenced by the mean proportions of donor and recipient genomes in the individuals of this population. These proportions differ according to the cross type:

$$\mu_{F1}= \frac{\mu_{D}+\mu_{R}}{2}, \mu_{BC1}= \frac{1}{4}\mu_{D}+\frac{3}{4}\mu_{R} and \mu_{BC2}= \frac{1}{8}\mu_{D}+\frac{7}{8}\mu_{R} (4)$$

where $\mu_{X}$ is the expected mean performance value in the $X$ population and in the populations derived from it ($X \in\{F1, BC1, BC2\}$).

The performance gaps between the different populations and the recipient line can be expressed as:

$${Gap}_{F1}=\mu_{F1}-\mu_{R}= \frac{{Gap}_{D}}{2}, {Gap}_{BC1}=\mu_{BC1}-\mu_{R}= \frac{{Gap}_{D}}{4}$$

$$and {Gap}_{BC2}=\mu_{BC2}-\mu_{R}= \frac{{Gap}_{D}}{8} (5)$$

where ${Gap}_{D}= \mu_{D}- \mu_{R}$

## Genetic variance

### Expression of the genetic variance for an additive trait with QTLs in linkage equilibrium

We consider a purely additive trait determined by Q biallelic QTLs. For each QTL, D and R lines are homozygous for a different allele. We note $x_{iq}$ the number of R alleles carried by the individual $i$ at the QTL ${(x}_{iq} \in\left\{ 0;1;2 \right\})$. We define $\beta_{q}$ the allelic substitution effect for the QTL $q$. $\sum_{q=1}^{Q} x_{iq}\beta_{q}$ is the genetic value of the individual $i$

Assuming that the QTLs are in linkage equilibrium,

we have:

$$\sigma_{G}^{2}=var\left( \sum_{q=1}^{Q} x_{iq}\beta_{q} \right)=\sum_{q=1}^{Q} {var(x}_{iq}\beta_{q})= \sum_{q=1}^{Q} {var(x}_{iq}) \beta_{q}^{2} (6)$$

For a given QTL $q$:

$${var(x}_{iq})=f\left( DD \right)_{q}*\left( 0-x_{.q} \right)^{2}+ f\left( DR \right)_{q}*\left( 1-x_{.q} \right)^{2}+ f\left( RR \right)_{q}*\left( 2-x_{.q} \right)^{2} (7)$$

where $f\left( DD \right)$, $f\left( DR \right)$ and $f\left( RR \right)$ are the genotypic frequency at the QTL in the population $q$ and $x_{.q}$ the mean value of allelic doses ($x_{.q}= f\left( DR \right)_{q}+2*f\left( RR \right)_{q}$).

### Application to DH populations

We note $DH_{X}$ a population of DH lines derived from a cross type $X with X \in\{F1, BC1, BC2\}$.

**Table 1** Genotypic variances for different population structures

| Population | $f\left( DD \right)$ | $f\left( DR \right)$ | $f\left( RR \right)$ |
| --- | --- | --- | --- |
| F1 | 0 | 1 | 0 |
| $DH_{F1}$ | $\frac{1}{2}$ | $0$ | $\frac{1}{2}$ |
| BC1 | 0 | $\frac{1}{2}$ | $\frac{1}{2}$ |
| $DH_{BC1}$ | $\frac{1}{4}$ | 0 | $\frac{3}{4}$ |
| BC2 | 0 | $\frac{1}{4}$ | $\frac{3}{4}$ |
| $DH_{BC2}$ | $\frac{1}{8}$ | 0 | $\frac{7}{8}$ |

At a given QTL, the genotypic frequencies differ according to the population structure (**Table 1**). Using these frequencies and $\left( 2 \right)$, we can give an expression of the value of $\sigma_{G}^{2}$ for the different DH populations:

$${\sigma_{G}^{2}}_{DH_{F1}}=\sum_{q=1}^{Q} \beta_{q}^{2}, {\sigma_{G}^{2}}_{DH_{BC1}}=\frac{3}{4}\sum_{q=1}^{Q} \beta_{q}^{2} and {\sigma_{G}^{2}}_{DH_{BC2}}=\frac{7}{16}\sum_{q=1}^{Q} \beta_{q}^{2} (8)$$

### Application to SSD populations

We note $XSn$ a population of lines derived from a cross type $X$ with $n$ generations of selfing. We will consider only the expression of the genotypic frequencies for populations derived from $BC1$ which corresponds to the populations evaluated in our paper.

In the $BC1,$ at a given QTL, we have $f\left( DR \right)=\frac{1}{2} and f\left( RR \right)=\frac{1}{2}$. The genotypic frequencies after n selfing generations in progeny derived from the BC1 individuals carrying DR genotype are:

$$f\left( RD \right)=\frac{1}{2^{n}} and f\left( RR \right)=f\left( DD \right)=\frac{1}{4} \left( 1+\frac{1}{2}+\frac{1}{4}+\ldots+\frac{1}{2^{n-1}} \right)= \frac{1}{4} \left( \frac{{\frac{1}{2}}^{n}-1}{\frac{1}{2}-1} \right)=\frac{1}{2}-{\frac{1}{2}}^{n+1}$$

For those derived from the BC1 individuals carrying RR genotype, we have $f\left( RR \right)=1$.

Thus, we can give the expression of the genotypic frequency in a population derive from a BC1 cross after n selfing generations:

$$f\left( DD \right)=\frac{1}{2}*\left( \frac{1}{2}-{\frac{1}{2}}^{n+1} \right)=\frac{1}{4}-{\frac{1}{2}}^{n+2}, f\left( DR \right)= \frac{1}{2}*\left( {\frac{1}{2}}^{n} \right)={\frac{1}{2}}^{n+1}$$

$$and f\left( RR \right)=\frac{1}{2}+ \frac{1}{2}*\left( \frac{1}{2}-{\frac{1}{2}}^{n+1} \right)=\frac{3}{4}-{\frac{1}{2}}^{n+2}$$

In this case,

$${var(x}_{iq})=\left( \frac{1}{4}-{\frac{1}{2}}^{n+2} \right)*\left( 0-\frac{3}{2} \right)^{2}+ {\frac{1}{2}}^{n+1}*\left( 1-\frac{3}{2} \right)^{2}+\left( \frac{3}{4}-{\frac{1}{2}}^{n+2} \right)*\left( 2-\frac{3}{2} \right)^{2}=\frac{3}{4}-\left( \frac{1}{2} \right)^{n+1}$$

and

$${\sigma_{G}^{2}}_{BC1Sn}=\left( \frac{3}{4}-\left( \frac{1}{2} \right)^{n+1} \right)\sum_{q=1}^{Q} \beta_{q}^{2}$$

For $n=2,$ we get ${\sigma_{G}^{2}}_{BC1S2}=\frac{5}{8}\sum_{q=1}^{Q} \beta_{q}^{2} (9)$.

1. **Optimal cross type to maximize the UC**

We wanted to determine which cross type maximizes the performance gain for virtual DxR crosses. Each DxR cross is defined thanks to two parameters: the performance gap between the donor and recipient lines ($Gap_{D}$) and the genetic variance in a DH population derived from a F1 cross (${\sigma_{G}^{2}}_{DH_{F1}}$).

In our study, we evaluated the mean performances ($\mu_{BC1}$) and the genetic variances (${\sigma_{G}^{2}}_{DH_{F1}}$) of 20 BC1S2 families. We also obtained the performance of their recipient lines ($\mu_{R}$). We used these values to determine realistic ranges of both parameters.

We had access to $Gap_{D}$ as following:

$$Gap_{D}=4*Gap_{BC1}= 4*\left( \mu_{BC1}-\mu_{R} \right) (10)$$

We also computed the expected genetic variances in DH populations derived from F1 using those estimated in BC1S2 populations, (8) and (9):

$${\sigma_{G}^{2}}_{DH_{F1}}=\frac{8}{5} {\sigma_{G}^{2}}_{BC1S2} (11)$$

We applied $(8)$ and $(9)$ to each cross to obtain the values given in **Table 2**.

**Table 2** Mean performances ($\mu_{BC1}$) and genetic variances (${\sigma_{G}^{2}}_{DH_{F1}}$) of the 20 evaluated BC1S2 families and corresponding computed values of performance gaps between the donor and recipient lines ($Gap_{D}$) and the genetic variance in a DH population derived from F1 (${\sigma_{G}^{2}}_{DH_{F1}}$).

| Family | $Gap_{BC1}$ | ${\sigma_{G}^{2}}_{BC1S2}$ | $Gap_{D}$ | ${\sigma_{G}^{2}}_{DH_{F1}}$ |
| --- | --- | --- | --- | --- |
| A1D8 | -1.18 | 0.32 | -2.36 | 0.51 |
| A1D5 | -0.30 | 0.01 | -0.59 | 0.02 |
| A1D9 | -0.34 | 0.13 | -0.67 | 0.21 |
| A2D2 | -0.22 | 0.15 | -0.45 | 0.25 |
| A2D4 | 0.21 | 0.12 | 0.42 | 0.20 |
| A3D1 | -0.81 | 0.08 | -1.63 | 0.13 |
| A3D4 | -0.72 | 0.09 | -1.44 | 0.15 |
| A3D6 | -0.83 | 0.14 | -1.66 | 0.22 |
| A4D3 | -0.39 | 0 | -0.77 | 0 |
| A4D4 | -0.54 | 0.01 | -1.07 | 0.01 |
| A4D6 | -0.27 | 0 | -0.54 | 0 |
| A5D1 | -0.53 | 0.14 | -1.06 | 0.23 |
| A5D2 | -0.65 | 0 | -1.29 | 0 |
| A5D7 | -0.34 | 0.16 | -0.67 | 0.26 |
| A6D3 | -1.02 | 0.03 | -2.04 | 0.04 |
| A6D5 | -0.37 | 0.08 | -0.75 | 0.13 |
| A6D7 | -0.93 | 0.12 | -1.86 | 0.19 |
| A7D3 | -0.27 | 0 | -0.54 | 0 |
| A7D5 | -0.50 | 0.08 | -1.01 | 0.13 |
| A7D6 | -0.69 | 0 | -1.38 | 0 |

We defined the following range for both parameters:

$$Gap_{D} \in\left[ -3,1 \right], {\sigma_{G}^{2}}_{DH_{F1}} \in\left[ 0,0.6 \right]$$

We considered a step of 0.005 for both parameters. For each parameter pair, we computed the gains obtained for DH populations derived from F1, BC1 or BC2 crosses using (5) and (8). The cross type which maximized it was considered as optimal.

**Table S1** Names and pedigrees of donor lines involved in the creation of the plant material

| **Donor line** | **Name** | **Pedigree** | **Collection** | **Country** |
| --- | --- | --- | --- | --- |
| D1 | F77 | Pop. Liesse-3.1.2 (PPS 19) | CornFed^(1)^ | France |
| D2 | PP87 | Pop. Isola Basso | CornFed^(1)^ | Italy |
| D3 | F7131 (PDZ502_SSD14) | Aranga 1_14-01m-4m-1m-m | Diversity Zea Promaïs | Spain |
| D4 | EC50 | Composite Aranga x EC18 | CornFed^(1)^ | Spain |
| D5 | LP1061 | Compuesto Colorado Precoz | INRAE SMH  Collection | Argentina, France, Canada, Roumania |
| D6 | F7129  (PDZ12_SSD07) | VA5 Nostrano dell Isola_07-01m-5m-1m-1 | Diversity Zea Promaïs | Italy |
| D7 | F7130 (PDZ502_SSD11) | Aranga 1_11-01m-2m-1m-m | Diversity Zea Promaïs | Spain |
| D8 | QSF3 | Pop. Quarantin de la Sainte Famille | INRAE SMH  Collection | Italy |
| D9 | Virtual line | - | - | - |

D1-D7 are initial selected donor lines, D8 and D9 are putative donor lines for A1D2 and A1D7 individuals respectively identified after genotyping analysis. (1) European project « Cornfed » Rincent, Renaud, et al. "Dent and Flint maize diversity panels reveal important genetic potential for increasing biomass production." Theoretical and Applied Genetics 127.11 (2014): 2313-2331

**Table S2** Adjusted means of the reference hybrids from the model M_FG

| **Trait** | **Trial** | **A1** | **A2** | **A3** | **A4** | **A5** | **A6** | **A7** |
| --- | --- | --- | --- | --- | --- | --- | --- | --- |
| **FLOM** | Blo19 | 90.41 | 91.3 | 91.42 | 91.85 | 90.96 | 91.98 | 91.73 |
|  | Smh19 | 55.13 | 54.84 | 55.73 | 55.18 | 56.51 | 55.99 | 57.54 |
|  | Vil19 | 92.49 | 93.58 | 92.49 | 94.61 | 94.1 | 94.11 | 93.84 |
|  | **Multi** | **79.34** | **79.9** | **79.88** | **80.55** | **80.52** | **80.69** | **81.04** |
| **FLOF** | Blo19 | 90.76 | 89.95 | 89.12 | 89.31 | 88.81 | 91.8 | 91.69 |
|  | Smh19 | 55.78 | 54.33 | 55.04 | 53.83 | 55.51 | 55.64 | 57.86 |
|  | Vil19 | 93.44 | 92.8 | 91.92 | 92.38 | 94.2 | 93.96 | 97.1 |
|  | **Multi** | **79.99** | **79.03** | **78.69** | **78.5** | **79.51** | **80.47** | **82.22** |
| **ASI** | Blo19 | 0.15 | 1.77 | 2.48 | 3.22 | 2.55 | -0.08 | 0.41 |
|  | Smh19 | -0.58 | 0.5 | 0.67 | 1.35 | 0.96 | 0.33 | -0.25 |
|  | Vil19 | -0.89 | 0.62 | 0.51 | 2.44 | -0.18 | -0.09 | -3.1 |
|  | **Multi** | **-0.44** | **0.96** | **1.22** | **2.33** | **1.11** | **0.05** | **-0.98** |
| **PH** | Blo19 | 267.96 | 253.88 | 267.47 | 292.92 | 253.85 | 268.16 | 282.23 |
|  | Smh19 | 303.8 | 288.2 | 306.38 | 333.28 | 302.53 | 296.66 | 313.96 |
|  | **Multi** | **285.88** | **271.04** | **286.93** | **313.1** | **278.19** | **282.41** | **298.09** |
| **GY** | Blo19 | 12.98 | 12.31 | 11.65 | 13.00 | 12.58 | 11.70 | 11.08 |
|  | Smh19 | 11.77 | 11.53 | 12.80 | 12.37 | 12.91 | 12.57 | 11.35 |
|  | Vil19 | 10.56 | 8.70 | 10.88 | 10.40 | 10.93 | 10.15 | 8.28 |
|  | **Multi** | **11.77** | **10.85** | **11.77** | **11.93** | **12.14** | **11.47** | **10.24** |
| **H2O** | Blo19 | 28.49 | 27.02 | 25.52 | 27.43 | 28.12 | 25.8 | 29.35 |
|  | Smh19 | 24.54 | 21.79 | 22.48 | 25.17 | 23.26 | 23.59 | 22.94 |
|  | Vil19 | 30.3 | 27.54 | 26.64 | 26.07 | 30.33 | 27.06 | 28.32 |
|  | **Multi** | **27.78** | **25.45** | **24.88** | **26.22** | **27.24** | **25.49** | **26.87** |
| **YI** | Blo19 | 13.19 | 12.81 | 12.62 | 13.47 | 12.85 | 12.60 | 11.00 |
|  | Smh19 | 11.66 | 12.10 | 13.20 | 12.11 | 13.12 | 12.70 | 11.64 |
|  | Vil19 | 10.10 | 8.93 | 11.33 | 10.99 | 10.46 | 10.49 | 8.31 |
|  | **Multi** | **11.65** | **11.28** | **12.38** | **12.19** | **12.14** | **11.93** | **10.31** |

The adjusted means were computed with fixed parameters of M_FG for the referent hybrid in each trial (Blo19, Smh19 and Vil19) and in multi-trial (Multi).

**Table S3** Adjusted means of the hybrid families from the model M_FG

| **Trait** | **Trial** | **A1D8** | **A1D5** | **A1D9** | **A2D2** | **A2D4** | **A3D1** | **A3D4** | **A3D6** | **A4D3** | **A4D4** | **A4D6** | **A5D1** | **A5D2** | **A5D7** | **A6D3** | **A6D5** | **A6D7** | **A7D3** | **A7D5** | **A7D6** |
| --- | --- | --- | --- | --- | --- | --- | --- | --- | --- | --- | --- | --- | --- | --- | --- | --- | --- | --- | --- | --- | --- |
| **FLOM** | Blo19 | 90.65 | 92.78 | 90.09 | 90.49 | 92.51 | 91.56 | 93.15 | 92.15 | 91.58 | 92.45 | 92.33 | 89.46 | 90.88 | 91.11 | 93.1 | 92.21 | 93.41 | 91.35 | 91.38 | 91.46 |
|  | Smh19 | 55.91 | 57.78 | 55.52 | 55.52 | 55.87 | 56.23 | 57.69 | 56.6 | 55.99 | 57.35 | 56.32 | 55.77 | 57.62 | 57.27 | 57.16 | 56.45 | 57.53 | 56.57 | 56.72 | 56.79 |
|  | Vil19 | 93.54 | 95.13 | 93.29 | 94.15 | 94.93 | 93.96 | 95.67 | 94.06 | 94.48 | 95.07 | 94.69 | 92.81 | 93.83 | 94.77 | 94.84 | 93.99 | 95.16 | 93.6 | 93.83 | 93.45 |
|  | **Multi** | **80.04** | **81.9** | **79.63** | **80.05** | **81.1** | **80.58** | **82.17** | **80.94** | **80.68** | **81.62** | **81.11** | **79.34** | **80.78** | **81.05** | **81.7** | **80.88** | **82.03** | **80.51** | **80.64** | **80.56** |
| **FLOF** | Blo19 | 91.55 | 93.37 | 90.36 | 89.72 | 91.13 | 90.01 | 92.02 | 90.62 | 89.8 | 91.36 | 90.29 | 88.16 | 89.24 | 89.9 | 93.00 | 92.11 | 93.21 | 91.5 | 91.31 | 90.9 |
|  | Smh19 | 56.82 | 58.28 | 55.87 | 55.39 | 55.69 | 55.13 | 56.96 | 55.54 | 54.86 | 56.24 | 54.95 | 54.98 | 56.84 | 56.7 | 56.96 | 56.35 | 57.99 | 57.22 | 57.45 | 57.38 |
|  | Vil19 | 95.01 | 96.55 | 94.18 | 93.67 | 94.73 | 93.37 | 95.01 | 93.48 | 93.13 | 94.48 | 93.37 | 93.15 | 93.96 | 94.82 | 95.43 | 94.74 | 96.05 | 96.12 | 96.39 | 95.88 |
|  | **Multi** | **81.13** | **82.73** | **80.14** | **79.6** | **80.51** | **79.5** | **81.33** | **79.88** | **79.26** | **80.7** | **79.54** | **78.76** | **80.02** | **80.47** | **81.79** | **81.07** | **82.42** | **81.61** | **81.72** | **81.39** |
| **ASI** | Blo19 | -0.4 | -0.08 | 0.23 | 1.12 | 1.74 | 1.71 | 1.28 | 1.69 | 2.35 | 1.64 | 2.67 | 1.69 | 2.03 | 1.6 | -0.1 | -0.1 | -0.03 | 0.24 | 0.35 | 0.72 |
|  | Smh19 | -0.84 | -0.4 | -0.3 | 0.09 | 0.17 | 1.08 | 0.72 | 1.05 | 1.15 | 1.04 | 1.31 | 0.7 | 0.76 | 0.52 | 0.17 | 0.11 | -0.51 | -0.58 | -0.69 | -0.51 |
|  | Vil19 | -1.47 | -1.37 | -0.82 | 0.3 | 0.04 | 0.59 | 0.62 | 0.56 | 1.58 | 0.79 | 1.52 | -0.44 | -0.21 | -0.05 | -0.8 | -1.03 | -1.16 | -2.34 | -2.33 | -2.32 |
|  | **Multi** | **-0.9** | **-0.61** | **-0.29** | **0.5** | **0.65** | **1.12** | **0.87** | **1.1** | **1.7** | **1.16** | **1.83** | **0.65** | **0.86** | **0.69** | **-0.25** | **-0.34** | **-0.57** | **-0.89** | **-0.89** | **-0.71** |
| **PH** | Blo19 | 271.91 | 278.9 | 271.85 | 258.77 | 267.59 | 270.69 | 282.85 | 276.59 | 285.96 | 291.51 | 291.3 | 252.31 | 257.79 | 267.31 | 273.64 | 270.75 | 276.78 | 283.09 | 283.88 | 281.87 |
|  | Smh19 | 300.55 | 308.4 | 303.04 | 294.21 | 300.35 | 306.95 | 317.11 | 311.33 | 321.69 | 322.91 | 324.07 | 301.41 | 308.05 | 310.22 | 306.89 | 306.28 | 311.66 | 306.87 | 314.19 | 304.19 |
|  | **Multi** | **286.23** | **293.65** | **287.45** | **276.49** | **283.97** | **288.82** | **299.98** | **293.96** | **303.83** | **307.21** | **307.69** | **276.86** | **282.92** | **288.76** | **290.26** | **288.51** | **294.22** | **294.98** | **299.04** | **293.03** |
| **GY** | Blo19 | 11.85 | 12.76 | 12.36 | 11.50 | 12.62 | 11.40 | 11.79 | 11.60 | 12.45 | 12.52 | 12.72 | 12.50 | 12.54 | 12.76 | 11.07 | 11.66 | 11.47 | 10.56 | 10.50 | 10.19 |
|  | Smh19 | 10.82 | 12.55 | 11.96 | 11.27 | 11.63 | 12.27 | 12.45 | 12.43 | 12.22 | 12.28 | 12.42 | 11.70 | 11.98 | 12.77 | 11.78 | 12.18 | 11.59 | 11.31 | 11.28 | 11.14 |
|  | Vil19 | 9.21 | 9.91 | 9.86 | 9.02 | 9.30 | 9.98 | 10.13 | 9.79 | 9.73 | 9.66 | 9.88 | 10.45 | 10.07 | 10.40 | 8.91 | 9.44 | 9.21 | 8.25 | 7.96 | 7.76 |
|  | **Multi** | **10.63** | **11.74** | **11.39** | **10.60** | **11.18** | **11.22** | **11.46** | **11.27** | **11.47** | **11.49** | **11.68** | **11.55** | **11.53** | **11.98** | **10.59** | **11.09** | **10.76** | **10.04** | **9.91** | **9.70** |
| **H2O** | Blo19 | 28.78 | 29.97 | 28.84 | 26.98 | 28.28 | 26.67 | 27.68 | 27.01 | 27.44 | 28.25 | 27.7 | 27.65 | 28.06 | 28.7 | 27.36 | 26.25 | 27.51 | 29.06 | 29.4 | 28.9 |
|  | Smh19 | 24.5 | 25.14 | 24.51 | 21.96 | 22.01 | 22.89 | 22.93 | 23.01 | 23.99 | 24.03 | 24.52 | 23.5 | 23.59 | 24.02 | 23.24 | 23.07 | 23.41 | 23.39 | 23.11 | 23.41 |
|  | Vil19 | 30.4 | 31.47 | 29.54 | 27.18 | 27.58 | 28.14 | 28.92 | 28.54 | 26.43 | 27.66 | 26.74 | 29.88 | 30.52 | 31.13 | 27.55 | 27.03 | 28.11 | 28.82 | 29.79 | 29.11 |
|  | **Multi** | **27.9** | **28.86** | **27.63** | **25.37** | **25.96** | **25.9** | **26.51** | **26.18** | **25.95** | **26.64** | **26.32** | **27.01** | **27.39** | **27.95** | **26.05** | **25.45** | **26.34** | **27.09** | **27.43** | **27.14** |
| **YI** | Blo19 | 11.99 | 12.59 | 12.49 | 12.01 | 12.80 | 12.08 | 12.22 | 12.22 | 12.91 | 12.79 | 13.12 | 12.89 | 12.82 | 12.90 | 11.59 | 12.45 | 11.93 | 10.54 | 10.40 | 10.23 |
|  | Smh19 | 10.72 | 12.29 | 11.86 | 11.80 | 12.15 | 12.57 | 12.75 | 12.70 | 12.25 | 12.29 | 12.32 | 11.85 | 12.11 | 12.79 | 12.00 | 12.43 | 11.76 | 11.49 | 11.53 | 11.33 |
|  | Vil19 | 8.72 | 9.16 | 9.59 | 9.34 | 9.52 | 10.06 | 10.02 | 9.77 | 10.24 | 9.86 | 10.32 | 10.09 | 9.55 | 9.73 | 9.14 | 9.80 | 9.30 | 8.16 | 7.62 | 7.59 |
|  | **Multi** | **10.48** | **11.35** | **11.31** | **11.05** | **11.49** | **11.57** | **11.66** | **11.56** | **11.80** | **11.65** | **11.92** | **11.61** | **11.49** | **11.81** | **10.91** | **11.56** | **11.00** | **10.06** | **9.85** | **9.72** |

The adjusted means were computed for the hybrid families in each trial (Blo19, Smh19 and Vil19) and in multi-trial (Multi). The estimations of fixed parameters of M_FG were used.

**Table S4** Differences between the reference hybrid and hybrid family adjusted mean values

| **Trait** | **Trial** | **A1D8** | **A1D5** | **A1D9** | **A2D2** | **A2D4** | **A3D1** | **A3D4** | **A3D6** | **A4D3** | **A4D4** | **A4D6** | **A5D1** | **A5D2** | **A5D7** | **A6D3** | **A6D5** | **A6D7** | **A7D3** | **A7D5** | **A7D6** | **Mean** |
| --- | --- | --- | --- | --- | --- | --- | --- | --- | --- | --- | --- | --- | --- | --- | --- | --- | --- | --- | --- | --- | --- | --- |
| **FLOM** | Blo19 | 0.25 | 2.37 | -0.32 | -0.81 | 1.21 | 0.14 | 1.73 | 0.74 | -0.28 | 0.59 | 0.48 | -1.5 | -0.07 | 0.15 | 1.13 | 0.23 | 1.43 | -0.38 | -0.34 | -0.27 | 0.32 |
|  | Smh19 | 0.78 | 2.65 | 0.39 | 0.68 | 1.03 | 0.5 | 1.96 | 0.87 | 0.81 | 2.17 | 1.14 | -0.74 | 1.11 | 0.76 | 1.17 | 0.45 | 1.54 | -0.97 | -0.82 | -0.76 | 0.74 |
|  | Vil19 | 1.05 | 2.64 | 0.8 | 0.58 | 1.36 | 1.46 | 3.17 | 1.57 | -0.13 | 0.46 | 0.08 | -1.29 | -0.27 | 0.67 | 0.73 | -0.12 | 1.05 | -0.24 | -0.01 | -0.39 | 0.66 |
|  | **Multi** | **0.69*** | **2.55*** | **0.29** | **0.15** | **1.2*** | **0.7*** | **2.29*** | **1.06*** | **0.13** | **1.07*** | **0.57*** | **-1.18*** | **0.26** | **0.53*** | **1.01*** | **0.19** | **1.34*** | **-0.53** | **-0.39** | **-0.47** | **0.57** |
| **FLOF** | Blo19 | 0.79 | 2.61 | -0.4 | -0.23 | 1.17 | 0.88 | 2.9 | 1.5 | 0.5 | 2.06 | 0.98 | -0.65 | 0.43 | 1.09 | 1.2 | 0.31 | 1.42 | -0.19 | -0.37 | -0.78 | 0.76 |
|  | Smh19 | 1.04 | 2.5 | 0.09 | 1.06 | 1.35 | 0.09 | 1.92 | 0.5 | 1.03 | 2.41 | 1.12 | -0.53 | 1.33 | 1.19 | 1.31 | 0.71 | 2.35 | -0.64 | -0.41 | -0.48 | 0.9 |
|  | Vil19 | 1.58 | 3.12 | 0.75 | 0.87 | 1.93 | 1.45 | 3.09 | 1.57 | 0.75 | 2.1 | 1,00 | -1.05 | -0.23 | 0.62 | 1.47 | 0.78 | 2.1 | -0.98 | -0.71 | -1.22 | 0.95 |
|  | **Multi** | **1.14*** | **2.74*** | **0.14** | **0.57*** | **1.49*** | **0.81*** | **2.64*** | **1.19*** | **0.76*** | **2.19*** | **1.03*** | **-0.74*** | **0.51*** | **0.97*** | **1.33*** | **0.6*** | **1.95*** | **-0.6*** | **-0.5** | **-0.83*** | **0.87** |
| **ASI** | Blo19 | -0.55 | -0.23 | 0.08 | -0.64 | -0.03 | -0.77 | -1.2 | -0.79 | -0.86 | -1.58 | -0.55 | -0.86 | -0.52 | -0.94 | -0.03 | -0.02 | 0.05 | -0.17 | -0.06 | 0.31 | -0.46 |
|  | Smh19 | -0.26 | 0.18 | 0.28 | -0.41 | -0.33 | 0.41 | 0.06 | 0.38 | -0.19 | -0.3 | -0.04 | -0.26 | -0.2 | -0.44 | -0.16 | -0.22 | -0.83 | -0.34 | -0.45 | -0.27 | -0.17 |
|  | Vil19 | -0.58 | -0.47 | 0.08 | -0.32 | -0.58 | 0.08 | 0.12 | 0.05 | -0.86 | -1.65 | -0.92 | -0.26 | -0.03 | 0.13 | -0.71 | -0.94 | -1.07 | 0.76 | 0.76 | 0.77 | -0.28 |
|  | **Multi** | **-0.46*** | **-0.17** | **0.15** | **-0.46*** | **-0.31*** | **-0.09** | **-0.34*** | **-0.12** | **-0.64*** | **-1.18*** | **-0.5*** | **-0.46*** | **-0.25*** | **-0.42*** | **-0.3*** | **-0.39*** | **-0.62*** | **0.08** | **0.08** | **0.27** | **-0.31** |
| **PH** | Blo19 | 3.94 | 10.93 | 3.89 | 4.89 | 13.71 | 3.21 | 15.38 | 9.11 | -6.96 | -1.41 | -1.62 | -1.54 | 3.94 | 13.46 | 5.48 | 2.59 | 8.62 | 0.86 | 1.65 | -0.36 | 4.49 |
|  | Smh19 | -3.25 | 4.59 | -0.76 | 6.02 | 12.15 | 0.57 | 10.73 | 4.95 | -11.59 | -10.37 | -9.21 | -1.11 | 5.52 | 7.69 | 10.23 | 9.61 | 15.00 | -7.09 | 0.23 | -9.77 | 1.71 |
|  | **Multi** | **0.35** | **7.76*** | **1.56** | **5.46** | **12.93*** | **1.89** | **13.06*** | **7.03*** | **-9.27*** | **-5.89** | **-5.41** | **-1.33** | **4.73** | **10.58*** | **7.85*** | **6.1** | **11.81*** | **-3.12** | **0.94** | **-5.07** | **3.10** |
| **GY** | Blo19 | -1.13 | -0.22 | -0.62 | -0.81 | 0.31 | -0.25 | 0.14 | -0.04 | -0.56 | -0.48 | -0.28 | -0.08 | -0.04 | 0.19 | -0.63 | -0.04 | -0.23 | -0.53 | -0.59 | -0.90 | -0.34 |
|  | Smh19 | -0.95 | 0.78 | 0.19 | -0.25 | 0.10 | -0.53 | -0.35 | -0.37 | -0.15 | -0.10 | 0.05 | -1.21 | -0.93 | -0.14 | -0.79 | -0.40 | -0.98 | -0.03 | -0.07 | -0.21 | -0.32 |
|  | Vil19 | -1.35 | -0.64 | -0.70 | 0.33 | 0.60 | -0.89 | -0.74 | -1.09 | -0.67 | -0.74 | -0.51 | -0.48 | -0.87 | -0.54 | -1.23 | -0.71 | -0.94 | -0.03 | -0.31 | -0.51 | -0.60 |
|  | **Multi** | **-1.14*** | **-0.03** | **-0.38*** | **-0.25** | **0.34*** | **-0.56*** | **-0.32*** | **-0.50*** | **-0.46*** | **-0.44*** | **-0.25** | **-0.59*** | **-0.61*** | **-0.16** | **-0.88*** | **-0.38*** | **-0.72*** | **-0.20** | **-0.32** | **-0.54*** | **-0.42** |
| **H2O** | Blo19 | 0.29 | 1.48 | 0.34 | -0.04 | 1.26 | 1.15 | 2.15 | 1.48 | 0.01 | 0.81 | 0.27 | -0.47 | -0.06 | 0.59 | 1.56 | 0.45 | 1.71 | -0.28 | 0.05 | -0.44 | 0.62 |
|  | Smh19 | -0.04 | 0.6 | -0.03 | 0.17 | 0.21 | 0.41 | 0.44 | 0.53 | -1.18 | -1.14 | -0.65 | 0.24 | 0.33 | 0.76 | -0.36 | -0.52 | -0.18 | 0.45 | 0.17 | 0.47 | 0.03 |
|  | Vil19 | 0.1 | 1.18 | -0.75 | -0.36 | 0.05 | 1.5 | 2.28 | 1.9 | 0.36 | 1.59 | 0.68 | -0.45 | 0.19 | 0.8 | 0.48 | -0.03 | 1.05 | 0.5 | 1.48 | 0.79 | 0.67 |
|  | **Multi** | **0.12** | **1.08*** | **-0.15** | **-0.08** | **0.51*** | **1.02*** | **1.63*** | **1.3*** | **-0.27** | **0.42*** | **0.1** | **-0.23** | **0.15** | **0.72*** | **0.56*** | **-0.03** | **0.86*** | **0.22** | **0.57*** | **0.27** | **0.44** |
| **YI** | Blo19 | -1.20 | -0.59 | -0.70 | -0.81 | -0.01 | -0.54 | -0.40 | -0.40 | -0.56 | -0.68 | -0.35 | 0.04 | -0.03 | 0.06 | -1.01 | -0.15 | -0.66 | -0.46 | -0.60 | -0.77 | -0.49 |
|  | Smh19 | -0.94 | 0.63 | 0.20 | -0.30 | 0.05 | -0.63 | -0.46 | -0.51 | 0.14 | 0.19 | 0.21 | -1.27 | -1.01 | -0.33 | -0.70 | -0.27 | -0.94 | -0.15 | -0.10 | -0.31 | -0.32 |
|  | Vil19 | -1.37 | -0.94 | -0.51 | 0.41 | 0.60 | -1.27 | -1.31 | -1.56 | -0.76 | -1.14 | -0.68 | -0.37 | -0.92 | -0.73 | -1.35 | -0.70 | -1.20 | -0.15 | -0.69 | -0.72 | -0.77 |
|  | **Multi** | **-1.17*** | **-0.30*** | **-0.34*** | **-0.23** | **0.21** | **-0.81*** | **-0.72*** | **-0.82*** | **-0.39*** | **-0.54*** | **-0.27*** | **-0.53*** | **-0.65*** | **-0.34*** | **-1.02*** | **-0.37*** | **-0.93*** | **-0.25** | **-0.46*** | **-0.60*** | **-0.53** |

Difference between the adjusted means of the hybrid families (Table S3) and the associated referent hybrid (Table S2) in each trial (Blo19, Smh19 and Vil19) and in multi-trial (Multi). For the multi-trial values, a star indicated that the difference is significant, after a Benjamini-Hochberg multiple testing correction to control the FDR level at a nominal level of 0.05 (see **File S3** for further details).

**Table S5** Percentages of individuals in each family with higher multi-trial adjusted means than their reference hybrid

| **Trait** | **A1** | | | **A2** | | **A3** | | | **A4** | | | **A5** | | | **A6** | | | **A7** | | |
| --- | --- | --- | --- | --- | --- | --- | --- | --- | --- | --- | --- | --- | --- | --- | --- | --- | --- | --- | --- | --- |
|  | **A1D8** | **A1D5** | **A1D9** | **A2D2** | **A2D4** | **A3D1** | **A3D4** | **A3D6** | **A4D3** | **A4D4** | **A4D6** | **A5D1** | **A5D2** | **A5D7** | **A6D3** | **A6D5** | **A6D7** | **A7D3** | **A7D5** | **A7D6** |
| ASI | 30% | 30% | 56% | 14% | 26% | 42% | 32% | 41% | 23% | 7% | 25% | 11% | 31% | 11% | 29% | 17% | 9% | 58% | 50% | 58% |
| FLOF | 77% | 98% | 58% | 76% | 89% | 78% | 100% | 79% | 63% | 90% | 72% | 27% | 71% | 77% | 93% | 71% | 98% | 34% | 50% | 33% |
| FLOM | 75% | 100% | 61% | 56% | 93% | 73% | 100% | 83% | 47% | 88% | 73% | 20% | 63% | 71% | 86% | 55% | 95% | 37% | 47% | 33% |
| PH | 52% | 82% | 59% | 75% | 91% | 63% | 96% | 79% | 21% | 30% | 33% | 49% | 63% | 86% | 77% | 77% | 91% | 39% | 54% | 31% |
| GY | 10% | 47% | 24% | 34% | 72% | 15% | 39% | 16% | 19% | 13% | 33% | 18% | 12% | 41% | 4% | 18% | 9% | 37% | 32% | 27% |
| H2O | 60% | 87% | 44% | 46% | 61% | 90% | 100% | 95% | 44% | 58% | 53% | 30% | 62% | 73% | 68% | 47% | 86% | 71% | 79% | 45% |
| YI | 10% | 25% | 27% | 39% | 63% | 8% | 25% | 9% | 28% | 13% | 27% | 20% | 8% | 29% | 5% | 29% | 0% | 26% | 29% | 30% |

The adjusted means of individuals were computed using model M_FG with genotypic effect declared as fixed.

| **Trait** | **AIC** | **BIC** | **Variance Type** | **A1D8** | **A1D5** | **A1D9** | **A2D2** | **A2D4** | **A3D1** | **A3D4** | **A3D6** | **A4D3** | **A4D4** | **A4D6** | **A5D1** | **A5D2** | **A5D7** | **A6D3** | **A6D5** | **A6D7** | **A7D3** | **A7D5** | **A7D6** | **Error** |
| --- | --- | --- | --- | --- | --- | --- | --- | --- | --- | --- | --- | --- | --- | --- | --- | --- | --- | --- | --- | --- | --- | --- | --- | --- |
| FLOM | 4877 | 5145 | $\boldsymbol{\sigma}_{\boldsymbol{G}}^{\boldsymbol{2}}$ | **1.16** (0.25) | **1.27** (0.27) | **1.80** (0.37) | **0.54** (0.15) | **0.46** (0.14) | **0.49** (0.12) | **0.64** (0.17) | **0.95** (0.24) | **0.51** (0.17) | **0.41** (0.13) | **0.42** (0.12) | **1.35** (0.27) | **1.08** (0.27) | **1.77** (0.38) | **0.53** (0.14) | **0.31** (0.10) | **0.39** (0.11) | **1.50** (0.43) | **2.11** (0.61) | **1.12** (0.38) | **-** |
|  |  |  | $\sigma_{GEBlo19}^{2}$ | **0**  (-) | **0**  (-) | **0.36** (0.21) | **0.90** (0.31) | **0.59** (0.26) | **0.47** (0.22) | **0.60** (0.27) | **0.64** (0.29) | **0**  (-) | **0.11** (0.16) | **0**  (-) | **0.27** (0.19) | **0.20** (0.21) | **0**  (-) | **0.36** (0.21) | **0.83** (0.27) | **0.56** (0.24) | **0.95** (0.42) | **0.30** (0.32) | **0**  (-) | **0.57** (0.04) |
|  |  |  | $\sigma_{GESmh19}^{2}$ | **0.17** (0.13) | **0.37** (0.16) | **0**  (-) | **0.16** (0.13) | **0.23** (0.14) | **0**  (-) | **0.30** (0.16) | **0.31** (0.19) | **0.87** (0.29) | **0.86** (0.24) | **0.99** (0.26) | **0.02** (0.12) | **0.43** (0.20) | **0.41** (0.19) | **0.13** (0.12) | **0.12** (0.11) | **0.08** (0.11) | **0.91** (0.36) | **0.97** (0.45) | **0.49** (0.30) | **0.26** (0.02) |
|  |  |  | $\sigma_{GEVil19}^{2}$ | **0**  (-) | **0**  (-) | **0**  (-) | **0.35** (0.24) | **0**  (-) | **0.58** (0.27) | **0.11** (0.21) | **0.52** (0.29) | **0**  (-) | **0**  (-) | **0**  (-) | **0.62** (0.28) | **0.29** (0.26) | **0.30** (0.25) | **0**  (-) | **0.27** (0.21) | **0**  (-) | **0.30** (0.31) | **0**  (-) | **0.51** (0.35) | **0.75** (0.04) |
| FLOF | 5562 | 5829 | $\boldsymbol{\sigma}_{\boldsymbol{G}}^{\boldsymbol{2}}$ | **1.62** (0.36) | **1.58** (0.36) | **2.00** (0.41) | **0.52** (0.15) | **0.67** (0.18) | **0.57** (0.15) | **0.67** (0.19) | **1.19** (0.28) | **2.31** (0.55) | **1.51** (0.33) | **1.94** (0.40) | **1.17** (0.25) | **1.18** (0.30) | **1.64** (0.36) | **0.71** (0.19) | **0.56** (0.14) | **0.43** (0.14) | **1.69** (0.52) | **2.86** (0.79) | **1.47** (0.5) | **-** |
|  |  |  | $\sigma_{GEBlo19}^{2}$ | **0.29** (0.25) | **0.59** (0.28) | **0.46** (0.24) | **0.18** (0.17) | **1.19** (0.37) | **0.71** (0.26) | **0.66** (0.29) | **0.61** (0.28) | **0.28** (0.23) | **1.13** (0.36) | **0.13** (0.17) | **0**  (-) | **0.09** (0.21) | **0.14** (0.20) | **0.28** (0.21) | **0.68** (0.25) | **0.48** (0.23) | **1.29** (0.53) | **0**  (-) | **0**  (-) | **0.55** (0.04) |
|  |  |  | $\sigma_{GESmh19}^{2}$ | **0.32** (0.22) | **0.62** (0.27) | **0**  (-) | **0.28** (0.16) | **0.10** (0.15) | **0**  (-) | **0.63** (0.25) | **0.26** (0.19) | **0**  (-) | **0.04** (0.16) | **0.04** (0.14) | **0.20** (0.15) | **0.82** (0.3) | **0.42** (0.21) | **0.32** (0.18) | **0**  (-) | **0.78** (0.26) | **1.12** (0.47) | **0.95** (0.44) | **0.52** (0.34) | **0.38** (0.03) |
|  |  |  | $\sigma_{GEVil19}^{2}$ | **1.14** (0.42) | **0.22** (0.27) | **0.24** (0.24) | **0.20** (0.22) | **0**  (-) | **0.16** (0.21) | **0**  (-) | **0.02** (0.21) | **0.09** (0.24) | **0.02** (0.21) | **0.46** (0.26) | **0.20** (0.21) | **0.41** (0.31) | **0.01**  (-) | **0.06** (0.21) | **0.15** (0.2) | **0**  (-) | **0.99** (0.51) | **0**  (-) | **1.32** (0.59) | **0.82** (0.05) |
| ASI | 576 | 844 | $\boldsymbol{\sigma}_{\boldsymbol{G}}^{\boldsymbol{2}}$ | **0.08** (0.03) | **0**  (-) | **0.06** (0.01) | **0.03** (0.02) | **0.05** (0.01) | **0.03** (0.02) | **0.07** (0.03) | **0.04** (0.04) | **0.03** (0.04) | **0.10** (0.04) | **0.07** (0.01) | **0.04** (0.02) | **0**  (-) | **0.02** (0.02) | **0.03** (0.02) | **0.01** (0.02) | **0**  (-) | **0**  (-) | **0.13** (0.04) | **0.07** (0.05) | **-** |
|  |  |  | $\sigma_{GEBlo19}^{2}$ | **0.15** (0.15) | **0.10** (0.13) | **0.16** (0.14) | **0.19** (0.14) | **0.44** (0.19) | **0.54** (0.21) | **0.54** (0.22) | **1.01** (0.31) | **0.78** (0.3) | **0.85** (0.27) | **0.88** (0.27) | **0.05** (0.11) | **0.06** (0.13) | **0.22** (0.16) | **0**  (-) | **0**  (-) | **0**  (-) | **0.42** (0.20) | **0.23** (0.20) | **0**  (-) | **0.59** (0.04) |
|  |  |  | $\sigma_{GESmh19}^{2}$ | **0.01** (0.03) | **0.07** (0.02) | **0**  (-) | **0**  (-) | **0**  (-) | **0.01** (0.02) | **0.01** (0.03) | **0.05** (0.04) | **0.04** (0.04) | **0.02** (0.04) | **0**  (-) | **0.02** (0.02) | **0.06** (0.03) | **0.01** (0.02) | **0.02** (0.02) | **0.03** (0.02) | **0.05** (0.01) | **0.04** (0.01) | **0**  (-) | **0.03** (0.05) | **0.01**  (0) |
|  |  |  | $\sigma_{GEVil19}^{2}$ | **0.84** (0.34) | **0.32** (0.24) | **0.17** (0.21) | **0.35** (0.25) | **0**  (-) | **0.58** (0.28) | **0.10** (0.2) | **0.32** (0.25) | **1.47** (0.54) | **0.44** (0.24) | **0.78** (0.31) | **0**  (-) | **0.26** (0.25) | **0**  (-) | **0**  (-) | **0.35** (0.24) | **0.01**  (-) | **0.47** (0.27) | **1.08** (0.46) | **1.25** (0.51) | **1.00** (0.06) |
| PH | 15463 | 15630 | $\boldsymbol{\sigma}_{\boldsymbol{G}}^{\boldsymbol{2}}$ | **57.94** (21.54) | **34.42** (18.84) | **69.35** (17.51) | **27.15** (13.76) | **1.37** (24.28) | **4.85** (11.21) | **15.59** (12.33) | **20.52** (14.97) | **25.00** (32.71) | **78.43** (24.38) | **56.58** (17.51) | **62.49** (17.92) | **60.64** (16.4) | **68.75** (21.82) | **45.67** (18.29) | **42.43** (15.2) | **40.39** (18.04) | **57.40** (27.5) | **50.22** (30.97) | **160.99** (69.34) | **-** |
|  |  |  | $\sigma_{GEBlo19}^{2}$ | **16.50** (31.21) | **50.34** (35.32) | **81.50** (42.06) | **31.31** (28.92) | **240.37** (72.64) | **0**  (-) | **0.85** (25.22) | **9.58** (26.71) | **296.60** (96.17) | **0**  (-) | **0**  (-) | **0**  (-)) | **27.85** (32.32) | **0**  (-) | **29.64** (31.68) | **23.90** (28.03) | **0.39** (26.03) | **49.99** (40.11) | **1.79** (36.78) | **102.11** (78.35) | **115.27** (7.43) |
|  |  |  | $\sigma_{GESmh19}^{2}$ | **30.58** (21.56) | **36.24** (21.16) | **0**  (-) | **10.27** (14.59) | **59.34** (29.57) | **39.18** (17.28) | **20.40** (15.07) | **35.37** (18.57) | **49.19** (36.35) | **34.83** (21.96) | **6.59** (14.83) | **4.69** (14.86) | **0**  (-) | **15.18** (18.75) | **15.70** (18.24) | **5.49** (14.57) | **44.17** (21.18) | **55.49** (29.93) | **63.49** (37.28) | **26.12** (54.86) | **27.76** (2.23) |
| GY | 21435 | 21702 | $\boldsymbol{\sigma}_{\boldsymbol{G}}^{\boldsymbol{2}}$ | **0.43** (0.12) | **0.06** (0.05) | **0.23** (0.07) | **0.27** (0.11) | **0.15** (0.08) | **0.08** (0.04) | **0.27** (0.09) | **0.14** (0.06) | **0.09** (0.06) | **0.04** (0.04) | **0.05** (0.04) | **0.25** (0.08) | **0.17** (0.08) | **0.20** (0.07) | **0.04** (0.04) | **0.03** (0.05) | **0.11** (0.06) | **0.37** (0.15) | **0.20** (0.14) | **0.87** (0.32) | **-** |
|  |  |  | $\sigma_{GEBlo19}^{2}$ | **0.19** (0.15) | **0.29** (0.15) | **0** (-) | **0.65** (0.24) | **0.04** (0.11) | **0** (-) | **0** (-) | **0** (-) | **0.05** (0.13) | **0** (-) | **0.02** (0.09) | **0.03** (0.11) | **0.05** (0.12) | **0** (-) | **0.31** (0.16) | **0.12** (0.11) | **0.27** (0.15) | **0.30** (0.2) | **0.33** (0.25) | **0.58** (0.38) | **0.46** (0.03) |
|  |  |  | $\sigma_{GESmh19}^{2}$ | **0** (-) | **0.05** (0.1) | **0** (-) | **0.35** (0.18) | **0.34** (0.17) | **0** (-) | **0** (-) | **0.06** (0.1) | **0** (-) | **0.12** (0.1) | **0** (-) | **0** (-) | **0.10** (0.12) | **0.14** (0.13) | **0** (-) | **0.11** (0.1) | **0** (-) | **0.35** (0.21) | **0.61** (0.28) | **0.55** (0.31) | **0.42** (0.02) |
|  |  |  | $\sigma_{GEVil19}^{2}$ | **0.17** (0.16) | **0** (-) | **0.13** (0.15) | **0.30** (0.18) | **0.46** (0.21) | **0** (-) | **0.43** (0.21) | **0** (-) | **0** (-) | **0** (-) | **0.15** (0.12) | **0.09** (0.13) | **0.23** (0.18) | **0.04** (0.13) | **0** (-) | **0.29** (0.16) | **0.57** (0.22) | **0.80** (0.33) | **0.07** (0.18) | **0.06** (0.25) | **0.54** (0.03) |
| H2O | 4160 | 4427 | $\boldsymbol{\sigma}_{\boldsymbol{G}}^{\boldsymbol{2}}$ | **0.68** (0.16) | **0.77** (0.18) | **0.45** (0.13) | **0.48** (0.14) | **0.49** (0.15) | **0.20** (0.07) | **0.35** (0.11) | **0.38** (0.11) | **0.09** (0.09) | **0.50** (0.14) | **0.40** (0.13) | **0.22** (0.08) | **0.79** (0.19) | **0.37** (0.11) | **0.67** (0.17) | **0.38** (0.10) | **0.35** (0.10) | **0.20** (0.15) | **0.43** (0.17) | **0.72** (0.25) | **-** |
|  |  |  | $\sigma_{GEBlo19}^{2}$ | **0.82** (0.30) | **0.28** (0.19) | **0.49** (0.25) | **0.72** (0.27) | **1.41** (0.41) | **0.27** (0.18) | **0.28** (0.20) | **0.13** (0.17) | **0.39** (0.24) | **0**  (-)) | **0.16** (0.17) | **0.05** (0.14) | **0.15** (0.19) | **0.46** (0.23) | **1.34** (0.40) | **0.48** (0.21) | **0.26** (0.18) | **1.60** (0.52) | **0.78** (0.40) | **1.03** (0.53) | **0.57** (0.05) |
|  |  |  | $\sigma_{GESmh19}^{2}$ | **0**  (-) | **0**  (-) | **0.04** (0.12) | **0.11** (0.13) | **0.07** (0.13) | **0**  (-) | **0**  (-) | **0**  (-) | **0.28** (0.15) | **0.82** (0.25) | **0.50** (0.18) | **0.06** (0.09) | **0.04** (0.13) | **0**  (-)) | **0**  (-) | **0**  (-) | **0**  (-) | **0.39** (0.19) | **0**  (-) | **0**  (-) | **0.33** (0.02) |
|  |  |  | $\sigma_{GEVil19}^{2}$ | **0.30** (0.16) | **0.83** (0.25) | **0.83** (0.26) | **0.65** (0.22) | **0.49** (0.21) | **0.26** (0.14) | **0.60** (0.21) | **0.40** (0.17) | **1.10** (0.34) | **0.31** (0.16) | **0.44** (0.18) | **0.83** (0.23) | **0.50** (0.21) | **0.82** (0.25) | **0.41** (0.18) | **0.51** (0.18) | **0.38** (0.17) | **1.28** (0.41) | **0.54** (0.25) | **1.17** (0.43) | **0.36** (0.03) |
| YI | 21713 | 21980 | $\boldsymbol{\sigma}_{\boldsymbol{G}}^{\boldsymbol{2}}$ | **0.37** (0.11) | **0.01** (0.05) | **0.15** (0.06) | **0.18** (0.09) | **0.14** (0.07) | **0.07** (0.05) | **0.30** (0.09) | **0.18** (0.07) | **0.08** (0.06) | **0.07** (0.05) | **0** (0.03) | **0.18** (0.06) | **0.09** (0.06) | **0.20** (0.08) | **0.06** (0.05) | **0.11** (0.06) | **0.14** (0.07) | **0.46** (0.19) | **0.37** (0.19) | **1.07** (0.36) | **-** |
|  |  |  | $\sigma_{GEBlo19}^{2}$ | **0.15** (0.15) | **0.34** (0.16) | **0** (-) | **0.38** (0.19) | **0** (-) | **0.10** (0.12) | **0** (-) | **0** (-) | **0.07** (0.14) | **0** (-) | **0.06** (0.1) | **0** (-) | **0** (-) | **0** (-) | **0.58** (0.22) | **0.28** (0.15) | **0.31** (0.17) | **0.20** (0.2) | **0.39** (0.29) | **0.71** (0.42) | **0.50** (0.03) |
|  |  |  | $\sigma_{GESmh19}^{2}$ | **0** (-) | **0.11** (0.11) | **0.02** (0.1) | **0.33** (0.17) | **0.33** (0.17) | **0.04** (0.1) | **0** (-) | **0.08** (0.11) | **0.02** (0.11) | **0.18** (0.12) | **0** (-) | **0** (-) | **0.14** (0.13) | **0.23** (0.15) | **0** (-) | **0.11** (0.11) | **0** (-) | **0.61** (0.28) | **0.43** (0.26) | **0.37** (0.26) | **0.44** (0.03) |
|  |  |  | $\sigma_{GEVil19}^{2}$ | **0.21** (0.18) | **0** (-) | **0.19** (0.16) | **0.34** (0.19) | **0.47** (0.23) | **0** (-) | **0.35** (0.2) | **0** (-) | **0** (-) | **0** (-) | **0.21** (0.14) | **0.10** (0.14) | **0.32** (0.2) | **0.05** (0.15) | **0** (-) | **0.20** (0.16) | **0.51** (0.23) | **0.78** (0.34) | **0.06** (0.2) | **0** (-) | **0.60** (0.04) |

**Table S6** Decomposition variance with the model M_FG_S_ (within-family genetic variances)

AIC and BIC criterion are indicated for each model. The estimations of variance components are indicated in bold and their standard errors are given in parenthesis. (The standard error is not computed if the estimated variance value is close to 0)

**Table S7** Likelihood ratio test to test the within-family variances homogeneity

| **Model 1** | **Model 2** | **Trait** | **Likelihood-ratio** | **DF** | **P-value** |
| --- | --- | --- | --- | --- | --- |
| M_FG | M_FG_S_ | FLOM | 238.15 | 76 | 5.83E-28 |
|  |  | FLOF | 229.89 | 76 | 1.52E-26 |
|  |  | ASI | 215.99 | 76 | 3.43E-24 |
|  |  | PH | 132.83 | 57 | 3,53E-24 |
|  |  | GY | 215.91 | 76 | 7,12E-14 |
|  |  | H2O | 192.51 | 76 | 2.66E-20 |
|  |  | YI | 203.64 | 76 | 3.94E-22 |
| M_FA | M_FA_S_ | FLOM | 200.82 | 76 | 1.15E-21 |
|  |  | FLOF | 242.63 | 76 | 9.87E-29 |
|  |  | ASI | 182.98 | 76 | 9.28E-19 |
|  |  | PH | 122.97 | 57 | 2.60E-12 |
|  |  | GY | 186.29 | 76 | 2.72E-19 |
|  |  | H2O | 164.39 | 76 | 8.11E-16 |
|  |  | YI | 184.24 | 76 | 5.82E-19 |
| M_FAP | M_FA_S_P_S_ | FLOM | 313.30 | 152 | 3.52E-27 |
|  |  | FLOF | 321.92 | 152 | 1.72E-28 |
|  |  | ASI | 223.99 | 152 | 1.62E-14 |
|  |  | PH | 180.08 | 114 | 5.55E-13 |
|  |  | GY | 253.51 | 152 | 1.72E-18 |
|  |  | H2O | 236.89 | 152 | 3.19E-16 |
|  |  | YI | 249.99 | 152 | 5.28E-18 |

The likelihood ratio test were performed with the function pchisqmix of the R package TcGSA which used a chi-squared mixtures distribution. P-value<0.01 were considered as significant. Likelihood-ratio: $2*(loglikelihood_{Model1}-loglikelihood_{Model2}$). DF: Degree of freedom (additional number of parameters in Model2 compared to Model1).

| **Trait** | **AIC** | **BIC** | **Variance Type** | **A1D8** | **A1D5** | **A1D9** | **A2D2** | **A2D4** | **A3D1** | **A3D4** | **A3D6** | **A4D3** | **A4D4** | **A4D6** | **A5D1** | **A5D2** | **A5D7** | **A6D3** | **A6D5** | **A6D7** | **A7D3** | **A7D5** | **A7D6** | **Error** |
| --- | --- | --- | --- | --- | --- | --- | --- | --- | --- | --- | --- | --- | --- | --- | --- | --- | --- | --- | --- | --- | --- | --- | --- | --- |
| FLOM | 4673 | 4941 | $\boldsymbol{\sigma}_{\boldsymbol{A}}^{\boldsymbol{2}}$ | **1.57** (0.37) | **1.14** (0.29) | **1.37** (0.32) | **0.64** (0.21) | **0.57** (0.19) | **0.68** (0.19) | **0.72** (0.23) | **1.03** (0.29) | **0.66** (0.26) | **1.14** (0.37) | **0.39** (0.14) | **1.37** (0.31) | **1.33** (0.35) | **2.29** (0.54) | **0.55** (0.16) | **0.34** (0.13) | **0.39** (0.13) | **2.21** (0.63) | **2.05** (0.62) | **1.66** (0.56) | **-** |
|  |  |  | $\sigma_{AEBlo19}^{2}$ | **0.03** (0.13) | **0** (-) | **0.21** (0.20) | **0.71** (0.37) | **0.43** (0.25) | **0.08** (0.16) | **0.51** (0.31) | **0.49** (0.34) | **0**  (-) | **0**  (-) | **0**  (-) | **0.17** (0.16) | **0.05** (0.18) | **0**  (-) | **0.02** (0.14) | **0.91** (0.42) | **0.43** (0.27) | **0.61** (0.40) | **0.09** (0.29) | **0**  (-) | **0.71** (0.04) |
|  |  |  | $\sigma_{AESmh19}^{2}$ | **0.03** (0.11) | **0.25** (0.18) | **0**  (-) | **0.09** (0.14) | **0.19** (0.15) | **0** (-) | **0.26** (0.18) | **0.24** (0.20) | **1.17** (0.45) | **1.94** (0.65) | **1.15** (0.37) | **0** (-) | **0.51** (0.25) | **0.28** (0.20) | **0.08** (0.10) | **0.13** (0.13) | **0.03** (0.09) | **1.45** (0.55) | **1.24** (0.61) | **0.40** (0.30) | **0.29** (0.02) |
|  |  |  | $\sigma_{AEVil19}^{2}$ | **0.08** (0.15) | **0** (0.11) | **0**  (-) | **0.27** (0.26) | **0**  (-) | **0.61** (0.30) | **0.20** (0.23) | **0.51** (0.32) | **0**  (-) | **0**  (-) | **0**  (-) | **0.80** (0.36) | **0.26** (0.25) | **0.27** (0.28) | **0**  (-) | **0.24** (0.20) | **0**  (-) | **0.10** (0.33) | **0** (-) | **0.47** (0.36) | **0.77** (0.04) |
| FLOF | 5386 | 5654 | $\boldsymbol{\sigma}_{\boldsymbol{A}}^{\boldsymbol{2}}$ | **2.12** (0.51) | **1.53** (0.40) | **1.65** (0.39) | **0.60** (0.19) | **0.64** (0.21) | **0.70** (0.20) | **0.89** (0.28) | **1.34** (0.35) | **4.85** (1.21) | **4.79** (1.06) | **2.65** (0.60) | **1.24** (0.30) | **1.45** (0.40) | **1.81** (0.45) | **0.75** (0.21) | **0.68** (0.20) | **0.37** (0.15) | **2.52** (0.77) | **2.78** (0.82) | **1.62** (0.57) | **-** |
|  |  |  | $\sigma_{AEBlo19}^{2}$ | **0** (-) | **0.46** (0.32) | **0.04** (0.16) | **0** (-) | **0.94** (0.40) | **0.40** (0.28) | **0.34** (0.32) | **0.36** (0.29) | **0.13** (0.20) | **1.21** (0.54) | **0.05** (0.15) | **0** (-) | **0** (-) | **0** (-) | **0.05** (0.17) | **0.37** (0.26) | **0.33** (0.26) | **0.87** (0.58) | **0** (-) | **0** (-) | **0.74** (0.04) |
|  |  |  | $\sigma_{AESmh19}^{2}$ | **0.43** (0.27) | **0.56** (0.31) | **0.01** (0.09) | **0.26** (0.15) | **0.09** (0.13) | **0** (-) | **0.50** (0.25) | **0.22** (0.21) | **0** (-) | **0** (-) | **0.06** (0.14) | **0.17** (0.13) | **1.01** (0.40) | **0.31** (0.20) | **0.14** (0.15) | **0.07** (0.12) | **0.57** (0.25) | **2.11** (0.80) | **1.14** (0.60) | **0.41** (0.32) | **0.40** (0.03) |
|  |  |  | $\sigma_{AEVil19}^{2}$ | **1.03** (0.52) | **0** (-) | **0** (-) | **0** (-) | **0** (-) | **0.02** (-) | **0** (-) | **0.05** (0.20) | **0** (-) | **0.01** (-) | **0.33** (0.26) | **0** (-) | **0.43** (0.34) | **0** (-) | **0** (-) | **0.04** (0.16) | **0** (-) | **0.85** (0.60) | **0** (-) | **1.03** (0.56) | **0.91** (0.04) |
| ASI | -355 | -87 | $\boldsymbol{\sigma}_{\boldsymbol{A}}^{\boldsymbol{2}}$ | **0.04** (0.02) | **0** (-) | **0.04** (0.01) | **0.02** (0.01) | **0.03** (0.02) | **0.03** (0.02) | **0.05** (0.02) | **0.03** (0.02) | **0.03** (0.04) | **0.05** (0.03) | **0.05** (0.01) | **0.02** (0.01) | **0** (0.02) | **0.01** (0.01) | **0.02** (0.02) | **0.01** (0.01) | **0** (-) | **0** (-) | **0.10** (0.03) | **0.04** (0.04) | **-** |
|  |  |  | $\sigma_{AEBlo19}^{2}$ | **0.07** (0.12) | **0** (-) | **0.08** (0.11) | **0.13** (0.13) | **0.33** (0.19) | **0.01**  (-) | **0.39** (0.23) | **0.98** (0.37) | **0.78** (0.38) | **0.95** (0.39) | **0.94** (0.37) | **0.04** (0.08) | **0.10** (0.12) | **0.19** (0.14) | **0** (-) | **0** (-) | **0** (-) | **0.38** (0.21) | **0.14** (0.19) | **0** (-) | **0.68** (0.03) |
|  |  |  | $\sigma_{AESmh19}^{2}$ | **0.02** (0.02) | **0.04** (0.01) | **0** (-) | **0** (0.01) | **0** (0.02) | **0** (0.02) | **0** (0.02) | **0.02** (0.02) | **0.02** (0.04) | **0.01** (0.03) | **0** (-) | **0.01** (0.01) | **0.05** (0.02) | **0** (0.01) | **0.01** (0.02) | **0.02** (0.01) | **0.03** (0.01) | **0.04** (0.01) | **0** (-) | **0.03** (0.04) | **0**  (0) |
|  |  |  | $\sigma_{AEVil19}^{2}$ | **0.61** (0.37) | **0** (-) | **0.07** (0.16) | **0.03** (0.14) | **0** (-) | **0.33** (0.25) | **0.10** (0.18) | **0.01**  (-) | **1.47** (0.68) | **0.07** (0.16) | **0.64** (0.37) | **0** (-) | **0** (-) | **0** (-) | **0** (-) | **0.12** (0.17) | **0** (-) | **0.39** (0.28) | **1.02** (0.54) | **1.06** (0.51) | **1.15** (0.05) |
| PH | 15384 | 15552 | $\boldsymbol{\sigma}_{\boldsymbol{A}}^{\boldsymbol{2}}$ | **58.94** (22.09) | **45.27** (26.3) | **49.97** (16.65) | **37.05** (16.75) | **0** (-) | **9.68** (9.67) | **18.93** (13.02) | **26.45** (17.55) | **78.03** (54.53) | **183.65** (53.8) | **93.83** (28.94) | **51.16** (17.75) | **53.77** (18.55) | **64.72** (20.01) | **51.74** (18.04) | **50.85** (17.37) | **45.17** (20.62) | **86.52** (34.84) | **55.40** (33.83) | **112.90** (71.21) | **-** |
|  |  |  | $\sigma_{AEBlo19}^{2}$ | **5.55** (26.26) | **28.76** (35.22) | **76.32** (41.62) | **0** (-) | **353.34** (117.43) | **0** (-) | **0** (-) | **0.55** (22.06) | **420.34** (156.52) | **0** (-) | **0** (-) | **0.49** (19.09) | **26.71** (28.82) | **0** (-) | **19.01** (27.02) | **5.22** (20.75) | **0** (-) | **0** (-) | **0** (-) | **76.46** (76.74) | **122.76** (6.26) |
|  |  |  | $\sigma_{AESmh19}^{2}$ | **10.48** (19.01) | **48.14** (30.06) | **0** (-) | **0.02** (-) | **65.74** (23.79) | **21.22** (14.4) | **11.57** (15.04) | **26.11** (20.74) | **8.79** (53.06) | **16.16** (22.11) | **0** (-) | **4.58** (13.5) | **0** (-) | **0** (-) | **0** (-) | **0** (-) | **26.74** (20.19) | **59.55** (34.88) | **64.27** (45.84) | **88.80** (71.77) | **35.47** (2.32) |
| GY | 21341 | 21608 | $\boldsymbol{\sigma}_{\boldsymbol{A}}^{\boldsymbol{2}}$ | **0.37** (0.12) | **0.06** (0.05) | **0.18** (0.07) | **0.21** (0.12) | **0.18** (0.1) | **0.09** (0.04) | **0.35** (0.12) | **0.13** (0.07) | **0.03** (0.05) | **0.03** (0.03) | **0.06** (0.04) | **0.26** (0.09) | **0.14** (0.08) | **0.21** (0.09) | **0.05** (0.04) | **0.01** (0.05) | **0.13** (0.08) | **0.49** (0.2) | **0.24** (0.18) | **1.39** (0.5) | **-** |
|  |  |  | $\sigma_{AEBlo19}^{2}$ | **0.16** (0.14) | **0.16** (0.14) | **0**  (-) | **0.64** (0.27) | **0.03** (0.1) | **0**  (-) | **0**  (-) | **0**  (-) | **0.14** (0.13) | **0**  (-) | **0** (0.05) | **0.10** (0.11) | **0.08** (0.12) | **0**  (-) | **0.22** (0.15) | **0.07** (0.08) | **0.22** (0.17) | **0.21** (0.21) | **0.31** (0.29) | **0.62** (0.45) | **0.48** (0.03) |
|  |  |  | $\sigma_{AESmh19}^{2}$ | **0.20** (0.15) | **0**  (-) | **0.07** (0.11) | **0.33** (0.22) | **0.50** (0.27) | **0** (0.08) | **0.32** (0.2) | **0.06** (0.1) | **0** (-) | **0**  (-) | **0.07** (0.1) | **0.12** (0.12) | **0.25** (0.18) | **0.09** (0.11) | **0**  (-) | **0.35** (0.18) | **0.63** (0.29) | **0.96** (0.42) | **0.04** (0.2) | **0.10** (0.28) | **0.55** (0.03) |
|  |  |  | $\sigma_{AEVil19}^{2}$ | **0**  (-) | **0.09** (0.09) | **0**  (-) | **0.31** (0.2) | **0.29** (0.18) | **0.01** (0.07) | **0** (-) | **0.10** (0.11) | **0.03** (0.08) | **0.08** (0.09) | **0**  (-) | **0.04** (0.09) | **0.11** (0.12) | **0.09** (0.11) | **0.01** (0.06) | **0.17** (0.11) | **0.01** (0.09) | **0.31** (0.23) | **0.79** (0.37) | **0.51** (0.35) | **0.43** (0.02) |
| H2O | 3753 | 4021 | $\boldsymbol{\sigma}_{\boldsymbol{A}}^{\boldsymbol{2}}$ | **0.56** (0.16) | **0.72** (0.20) | **0.43** (0.15) | **0.42** (0.15) | **0.37** (0.14) | **0.19** (0.08) | **0.41** (0.14) | **0.33** (0.11) | **0.10** (0.11) | **0.95** (0.26) | **0.47** (0.17) | **0.25** (0.10) | **0.71** (0.20) | **0.36** (0.12) | **0.50** (0.15) | **0.46** (0.14) | **0.23** (0.09) | **0.51** (0.26) | **0.59** (0.24) | **0.65** (0.25) | **-** |
|  |  |  | $\sigma_{AEBlo19}^{2}$ | **0.83** (0.38) | **0.35** (0.22) | **0.33** (0.23) | **0.70** (0.31) | **1.15** (0.40) | **0.19** (0.15) | **0.30** (0.21) | **0.15** (0.14) | **0.32** (0.25) | **0** (-) | **0.14** (0.18) | **0.14** (0.14) | **0.23** (0.19) | **0.34** (0.23) | **1.43** (0.49) | **0.48** (0.24) | **0.21** (0.16) | **1.51** (0.58) | **0.75** (0.44) | **0.92** (0.50) | **0.60** (0.04) |
|  |  |  | $\sigma_{AESmh19}^{2}$ | **0** (-) | **0** (-) | **0.11** (0.14) | **0.06** (0.12) | **0.05** (0.13) | **0** (-) | **0** (-) | **0** (-) | **0.25** (0.16) | **1.07** (0.38) | **0.56** (0.24) | **0.03** (0.10) | **0.05** (0.13) | **0** (-) | **0.02** (0.12) | **0** (-) | **0** (-) | **0.53** (0.30) | **0** (-) | **0** (-) | **0.32** (0.02) |
|  |  |  | $\sigma_{AEVil19}^{2}$ | **0.25** (0.16) | **0.78** (0.30) | **0.62** (0.27) | **0.73** (0.32) | **0.64** (0.29) | **0.23** (0.14) | **0.65** (0.27) | **0.36** (0.18) | **1.22** (0.46) | **0.02** (0.10) | **0.34** (0.20) | **0.97** (0.32) | **0.49** (0.24) | **0.74** (0.28) | **0.33** (0.19) | **0.43** (0.20) | **0.33** (0.18) | **1.67** (0.63) | **0.62** (0.34) | **1.55** (0.59) | **0.43** (0.03) |
| YI | 21648 | 21915 | $\boldsymbol{\sigma}_{\boldsymbol{A}}^{\boldsymbol{2}}$ | **0.31** (0.11) | **0.02** (0.04) | **0.12** (0.06) | **0.16** (0.11) | **0.16** (0.09) | **0.09** (0.05) | **0.39** (0.13) | **0.17** (0.08) | **0.03** (0.05) | **0.06** (0.05) | **0** (0.03) | **0.17** (0.08) | **0.05** (0.06) | **0.22** (0.1) | **0.05** (0.05) | **0.10** (0.07) | **0.18** (0.09) | **0.75** (0.27) | **0.48** (0.24) | **1.83** (0.6) | **-** |
|  |  |  | $\sigma_{AEBlo19}^{2}$ | **0.12** (0.13) | **0.13** (0.13) | **0**  (-) | **0.37** (0.21) | **0**  (-) | **0**  (-) | **0**  (-) | **0** (-) | **0.12** (0.14) | **0**  (-) | **0.02** (0.06) | **0.06** (0.1) | **0**  (-) | **0**  (-) | **0.47** (0.25) | **0.16** (0.14) | **0.18** (0.17) | **0** (0.19) | **0.25** (0.29) | **0.77** (0.52) | **0.55** (0.03) |
|  |  |  | $\sigma_{AESmh19}^{2}$ | **0.23** (0.16) | **0** (-) | **0.18** (0.14) | **0.32** (0.22) | **0.43** (0.26) | **0**  (-) | **0.30** (0.19) | **0.06** (0.11) | **0.04** (0.1) | **0**  (-) | **0.17** (0.13) | **0.16** (0.14) | **0.39** (0.22) | **0.11** (0.13) | **0**  (-) | **0.24** (0.17) | **0.58** (0.29) | **0.92** (0.41) | **0**  (-) | **0**  (-) | **0.60** (0.03) |
|  |  |  | $\sigma_{AEVil19}^{2}$ | **0**  (-) | **0.11** (0.1) | **0**  (-) | **0.31** (0.2) | **0.24** (0.17) | **0.03** (0.08) | **0**  (-) | **0.12** (0.11) | **0.09** (0.11) | **0.16** (0.12) | **0.03** (0.06) | **0.04** (0.09) | **0.15** (0.12) | **0.22** (0.16) | **0**  (-) | **0.16** (0.12) | **0**  (-) | **0.61** (0.32) | **0.51** (0.32) | **0.29** (0.25) | **0.46** (0.02) |

**Table S8** Variance decomposition with the model M_FA_s_ (within-family additive variances)

AIC and BIC criterion are indicated for each model. The estimations of variance components are indicated in bold and their standard errors are given in brackets. (The standard error is not computed if the estimated variance value is close to 0)

| **Trait** | **AIC** | **BIC** | **Variance**  **Type** | **A1D8** | **A1D5** | **A1D9** | **A2D2** | **A2D4** | **A3D1** | **A3D4** | **A3D6** | **A4D3** | **A4D4** | **A4D6** | **A5D1** | **A5D2** | **A5D7** | **A6D3** | **A6D5** | **A6D7** | **A7D3** | **A7D5** | **A7D6** | **Error** |
| --- | --- | --- | --- | --- | --- | --- | --- | --- | --- | --- | --- | --- | --- | --- | --- | --- | --- | --- | --- | --- | --- | --- | --- | --- |
| FLOM | 4681 | 5077 | $\boldsymbol{\sigma}_{\boldsymbol{A}}^{\boldsymbol{2}}$ | **1.33** (0.59) | **1.06** (0.27) | **1.35** (0.43) | **0.55** (0.18) | **0.13** (0.15) | **0.27** (0.2) | **0.45** (0.26) | **0.50** (0.29) | **0.15** (0.23) | **0** (-) | **0.42** (0.14) | **1.34** (0.4) | **1.29** (0.35) | **1.06** (0.51) | **0.52** (0.15) | **0.31** (0.15) | **0.36** (0.17) | **0.59** (0.63) | **2.01** (0.61) | **0** (-) | **0.51** (0.02) |
|  |  |  | $\boldsymbol{\sigma}_{\boldsymbol{G}}^{\boldsymbol{2}}$ | **0.07** (0.23) | **0** (-) | **0.01** (0.16) | **0** (-) | **0.26** (0.14) | **0.24** (0.15) | **0.13** (0.14) | **0.26** (0.18) | **0.35** (0.21) | **0.42** (0.13) | **0** (-) | **0.02** (0.13) | **0** (-) | **0.56** (0.31) | **0** (-) | **0.02** (0.07) | **0**  (-) | **0.80** (0.55) | **0** (-) | **1.17** (0.37) |  |
|  |  |  | $\overline{\boldsymbol{\sigma}_{\boldsymbol{AE}}^{\boldsymbol{2}}}$ | **0.06**  (-) | **0.01**  (-) | **0.04**  (-) | **0.06**  (-) | **0.25**  (-) | **0.23**  (-) | **0.24** (0.15) | **0.28**  (-) | **0.36**  (-) | **0.05**  (-) | **0.29**  (-) | **0.32**  (-) | **0.27** (0.15) | **0.07**  (-) | **0.04**  (-) | **0.13**  (-) | **0.03**  (-) | **0.31**  (-) | **0** (-) | **0.28**  (-) |  |
|  |  |  | $\overline{\boldsymbol{\sigma}_{\boldsymbol{GE}}^{\boldsymbol{2}}}$ | **0.05**  (-) | **0.12**  (-) | **0.09**  (-) | **0.44**  (-) | **0.03**  (-) | **0.16**  (-) | **0.16**  (-) | **0.25**  (-) | **0.01**  (-) | **0.28**  (-) | **0.06**  (-) | **0.06**  (-) | **0.05**  (-) | **0.18**  (-) | **0.14**  (-) | **0.29**  (-) | **0.20** (-) | **0.45**  (-) | **0.42**  (-) | **0** (-) |  |
| FLOF | 5389 | 5785 | $\boldsymbol{\sigma}_{\boldsymbol{A}}^{\boldsymbol{2}}$ | **1.24** (0.58) | **1.41** (0.39) | **1.51** (0.48) | **0.39** (0.21) | **0.29** (0.2) | **0.53** (0.24) | **0.24** (0.22) | **0.91** (0.41) | **0.62** (0.72) | **0.08** (0.26) | **1.66** (0.67) | **1.21** (0.29) | **1.04** (0.57) | **1.20** (0.52) | **0.73** (0.21) | **0.66** (0.19) | **0.27** (0.18) | **0.50** (0.71) | **2.81** (0.81) | **1.03** (0.76) | **0.56** (0.02) |
|  |  |  | $\boldsymbol{\sigma}_{\boldsymbol{G}}^{\boldsymbol{2}}$ | **0.39** (0.29) | **0** (-) | **0.02** (0.17) | **0.11** (0.12) | **0.24** (0.15) | **0.05** (0.13) | **0.32** (0.18) | **0.15** (0.23) | **1.68** (0.68) | **1.43** (0.39) | **0.40** (0.27) | **0.01**  (-) | **0.22** (0.3) | **0.31** (0.27) | **0** (-) | **0** (-) | **0.01** (0.1) | **1.10** (0.67) | **0** (-) | **0.45** (0.55) |  |
|  |  |  | $\overline{\boldsymbol{\sigma}_{\boldsymbol{AE}}^{\boldsymbol{2}}}$ | **0.18** (-) | **0.04** (-) | **0** (-) | **0.09** (-) | **0.40** (-) | **0** (-) | **0.20** (-) | **0.05** (-) | **0.11** (-) | **0** (-) | **0.11** (0.11) | **0.06** (-) | **0.39** (0.21) | **0.09** (-) | **0** (-) | **0.04** (-) | **0.26** (-) | **0.47** (-) | **0** (-) | **0.50** (-) |  |
|  |  |  | $\overline{\boldsymbol{\sigma}_{\boldsymbol{GE}}^{\boldsymbol{2}}}$ | **0.43** (-) | **0.44** (0.16) | **0.25** (-) | **0.14** (-) | **0.03** (-) | **0.35** (-) | **0.27** (-) | **0.31** (-) | **0.04** (-) | **0.41** (0.16) | **0.13** (-) | **0.11** (-) | **0.11** (-) | **0.11** (0.12) | **0.22** (0.11) | **0.25** (-) | **0.20** (-) | **0.67** (0.33) | **0.34**  (-) | **0** (-) |  |
| ASI | -247 | 149 | $\boldsymbol{\sigma}_{\boldsymbol{A}}^{\boldsymbol{2}}$ | **0.05** (0.03) | **0** (-) | **0.04** (0.01) | **0.01** (0.01) | **0.03** (0.02) | **0.03** (0.02) | **0.05** (0.02) | **0.03** (0.02) | **0.02** (0.04) | **0.06** (0.03) | **0.05** (0.01) | **0.02** (0.01) | **0**  (-) | **0.01** (0.01) | **0.02** (0.01) | **0.01** (0.01) | **0** (-) | **0** (-) | **0.09** (0.03) | **0.04** (0.04) | **0.52** (0.02) |
|  |  |  | $\boldsymbol{\sigma}_{\boldsymbol{G}}^{\boldsymbol{2}}$ | **0** (-) | **0** (-) | **0** (-) | **0** (-) | **0** (-) | **0** (-) | **0** (-) | **0** (-) | **0** (-) | **0** (-) | **0** (-) | **0** (-) | **0** (-) | **0** (-) | **0** (-) | **0** (-) | **0** (-) | **0** (-) | **0** (-) | **0** (-) |  |
|  |  |  | $\overline{\boldsymbol{\sigma}_{\boldsymbol{AE}}^{\boldsymbol{2}}}$ | **0.05** (0.11) | **0.01**  (-) | **0.07**  (-) | **0.05**  (-) | **0.09**  (-) | **0.04**  (-) | **0.09** (0.1) | **0.25**  (-) | **0.53** (0.31) | **0.01**  (-) | **0.21**  (-) | **0.03**  (-) | **0.06**  (-) | **0.08**  (-) | **0** (-) | **0.04** (0.07) | **0.01**  (-) | **0.24** (0.17) | **0** (-) | **0.39**  (-) |  |
|  |  |  | $\overline{\boldsymbol{\sigma}_{\boldsymbol{GE}}^{\boldsymbol{2}}}$ | **0.29**  (-) | **0.15**  (-) | **0.06**  (-) | **0.15**  (-) | **0.06**  (-) | **0.35**  (-) | **0.16**  (-) | **0.22**  (-) | **0.30** (-) | **0.46**  (-) | **0.38**  (-) | **0** (-) | **0.09**  (-) | **0** (-) | **0** (-) | **0.10** (-) | **0** (-) | **0.10** (-) | **0.46**  (-) | **0.01**  (-) |  |
| PH | 15408 | 15661 | $\boldsymbol{\sigma}_{\boldsymbol{A}}^{\boldsymbol{2}}$ | **58.28** (22.41) | **9.48** (23.28) | **52.29** (20.18) | **28.35** (22.77) | **15.51** (13.5) | **10.42** (9.52) | **19.08** (12.85) | **8.08** (12.92) | **0** (-) | **0.05**  (-) | **5.45** (15.21) | **53.58** (18.2) | **55.11** (28.13) | **63.75** (21.69) | **35.63** (21.86) | **31.27** (19.09) | **31.77** (22.27) | **0** (-) | **52.24** (30.09) | **95.86** (79.96) | **69.44** (3.54) |
|  |  |  | $\boldsymbol{\sigma}_{\boldsymbol{G}}^{\boldsymbol{2}}$ | **0** (-) | **24.20** (22.71) | **1.17** (10.47) | **6.02** (11.89) | **0** (-) | **0** (-) | **0** (-) | **12.44** (17.62) | **31.65** (32.58) | **78.29** (24.23) | **51.23** (21.34) | **0** (-) | **2.39** (19.35) | **0** (-) | **1.88** (17.99) | **12.98** (16.19) | **7.28** (17.21) | **57.60** (27.6) | **0** (-) | **5.75** (67.6) |  |
|  |  |  | $\overline{\boldsymbol{\sigma}_{\boldsymbol{AE}}^{\boldsymbol{2}}}$ | **12.35** (22.7) | **20.24** (24.73) | **41.40** (-) | **3.88**  (-) | **0.02**  (-) | **12.86** (-) | **5.63**  (-) | **3.69**  (-) | **10.86** (42.29) | **4.35** (11.88) | **0** (-) | **6.65** (12.34) | **16.64**  (-) | **0** (-) | **14.81**  (-) | **0** (-) | **10.01** (14.26) | **0** (-) | **0** (-) | **38.94**  (-) |  |
|  |  |  | $\overline{\boldsymbol{\sigma}_{\boldsymbol{GE}}^{\boldsymbol{2}}}$ | **5.11** (20.7) | **27.47** (25.87) | **0** (-) | **21.07**  (-) | **136.63** (35.02) | **0** (-) | **4.62** (13.92) | **20.04** (21.01) | **157.52** (65.05) | **13.66**  (-) | **3.40** (-) | **0** (-) | **0.68**  (-) | **4.58**  (-) | **13.22**  (-) | **17.48** (15.38) | **11.77**  (-) | **54.54** (25.11) | **32.16**  (-) | **44.50** (56.17) |  |
| GY | 21426 | 21821 | $\boldsymbol{\sigma}_{\boldsymbol{A}}^{\boldsymbol{2}}$ | **0.38**  (-) | **0**  (-) | **0.17**  (-) | **0.21** (0.08) | **0.11** (0.07) | **0.09** (0.03) | **0.19**  (-) | **0.12** (0.1) | **0**  (-) | **0.01** (0.04) | **0.06** (0.03) | **0.27**  (-) | **0.11** (0.1) | **0.17** (0.09) | **0.04**  (-) | **0.01** (0.04) | **0.10**  (-) | **0.30** (0.31) | **0.06** (0.17) | **0**  (-) | **0.45** (0.02) |
|  |  |  | $\boldsymbol{\sigma}_{\boldsymbol{G}}^{\boldsymbol{2}}$ | **0**  (0.11) | **0.04** (0.05) | **0.02** (0.07) | **0**  (0.13) | **0.03**  (0.1) | **0**  (0.05) | **0.12** (0.09) | **0.02** (0.09) | **0.09** (0.06) | **0.03** (0.04) | **0**  (0.05) | **0**  (0.08) | **0.03** (0.09) | **0.03** (0.1) | **0**  (0.04) | **0**  (-) | **0**  (0.06) | **0.11** (0.23) | **0.14** (0.21) | **0.89** (0.32) |  |
|  |  |  | $\overline{\boldsymbol{\sigma}_{\boldsymbol{AE}}^{\boldsymbol{2}}}$ | **0.14**  (-) | **0.04**  (-) | **0.02**  (-) | **0.28**  (-) | **0.03**  (0.1) | **0.01**  (-) | **0.05**  (-) | **0.07**  (-) | **0.05**  (-) | **0**  (-) | **0.01**  (-) | **0.10**  (-) | **0.17**  (-) | **0.05**  (-) | **0.02**  (-) | **0.21**  (-) | **0.01**  (-) | **0.22** (0.21) | **0**  (-) | **0.08** (0.19) |  |
|  |  |  | $\overline{\boldsymbol{\sigma}_{\boldsymbol{GE}}^{\boldsymbol{2}}}$ | **0**  (-) | **0.11**  (-) | **0.02**  (-) | **0.18** (0.12) | **0.30**  (-) | **0**  (-) | **0.10**  (-) | **0**  (-) | **0**  (-) | **0.05**  (-) | **0.04**  (-) | **0**  (-) | **0**  (-) | **0.06**  (-) | **0.10**  (-) | **0.03** (0.07) | **0.31**  (-) | **0.29** (0.19) | **0.36** (0.14) | **0.32**  (-) |  |
| H2O | 3825 | 4220 | $\boldsymbol{\sigma}_{\boldsymbol{A}}^{\boldsymbol{2}}$ | **0.56** (0.15) | **0.61** (0.25) | **0.44** (0.15) | **0.37** (0.14) | **0.32** (0.13) | **0.19** (0.08) | **0.07** (0.11) | **0.24** (0.13) | **0.09** (0.1) | **0.32** (0.22) | **0.47** (0.17) | **0.09** (0.1) | **0.67** (0.28) | **0.32** (0.11) | **0.50** (0.15) | **0.45** (0.14) | **0.23** (0.11) | **0** (-) | **0.39** (0.35) | **0.55** (0.36) | **0.40** (0.02) |
|  |  |  | $\boldsymbol{\sigma}_{\boldsymbol{G}}^{\boldsymbol{2}}$ | **0** (-) | **0.06** (0.11) | **0** (-) | **0** (-) | **0** (-) | **0** (-) | **0.25** (0.12) | **0.08** (0.09) | **0** (-) | **0.23** (0.16) | **0** (-) | **0.10** (0.08) | **0.03** (0.13) | **0** (-) | **0** (-) | **0** (-) | **0** (0.06) | **0.21** (0.14) | **0.06** (0.25) | **0.08** (0.25) |  |
|  |  |  | $\overline{\boldsymbol{\sigma}_{\boldsymbol{AE}}^{\boldsymbol{2}}}$ | **0.11**  (-) | **0.35**  (-) | **0.31** (0.15) | **0.33** (0.16) | **0.46** (0.17) | **0.13**  (-) | **0.39**  (-) | **0.19**  (-) | **0.47** (0.22) | **0.11**  (-) | **0.36** (0.13) | **0.42**  (-) | **0.29** (0.11) | **0.30** (-) | **0.45** (0.22) | **0.29**  (-) | **0.19**  (-) | **0.58** (0.35) | **0.01**  (-) | **0.32**  (-) |  |
|  |  |  | $\overline{\boldsymbol{\sigma}_{\boldsymbol{GE}}^{\boldsymbol{2}}}$ | **0.27**  (-) | **0.06**  (-) | **0.09**  (-) | **0.19**  (-) | **0.17**  (-) | **0.07**  (-) | **0** (-) | **0.01**  (-) | **0.17**  (-) | **0.27**  (-) | **0.03**  (-) | **0.03**  (-) | **0** (-) | **0.18**  (-) | **0.15**  (-) | **0.07**  (-) | **0.04**  (-) | **0.54** (0.28) | **0.44**  (-) | **0.43**  (-) |  |
| YI | 21728 | 22123 | $\boldsymbol{\sigma}_{\boldsymbol{A}}^{\boldsymbol{2}}$ | **0.32**  (-) | **0.01**  (-) | **0.13**  (-) | **0.15** (0.06) | **0.12** (0.06) | **0.08** (0.04) | **0.09**  (-) | **0.14** (0.08) | **0**  (-) | **0.01** (0.04) | **0**  (0.03) | **0.14**  (-) | **0**  (-) | **0.16** (0.08) | **0.03**  (-) | **0.08** (0.06) | **0.12**  (-) | **0**  (-) | **0.08** (0.22) | **0**  (-) | **0.48** (0.02) |
|  |  |  | $\boldsymbol{\sigma}_{\boldsymbol{G}}^{\boldsymbol{2}}$ | **0**  (0.1) | **0**  (0.05) | **0**  (0.06) | **0**  (0.11) | **0**  (0.09) | **0**  (0.06) | **0.23** (0.09) | **0.04** (0.1) | **0.06** (0.06) | **0.06** (0.06) | **0**  (0.04) | **0.03** (0.06) | **0.08** (0.06) | **0.03** (0.11) | **0.04** (0.05) | **0**  (0.08) | **0**  (0.07) | **0.48** (0.19) | **0.28** (0.27) | **1.09** (0.36) |  |
|  |  |  | $\overline{\boldsymbol{\sigma}_{\boldsymbol{AE}}^{\boldsymbol{2}}}$ | **0.14**  (-) | **0.05**  (-) | **0.06**  (-) | **0.19**  (-) | **0**  (-) | **0.01**  (-) | **0.07**  (-) | **0.07**  (-) | **0.10**  (-) | **0.01**  (-) | **0.04** (0.05) | **0.11**  (-) | **0.20**  (-) | **0.08**  (-) | **0.03**  (-) | **0.19**  (-) | **0.02**  (-) | **0.26** (0.22) | **0** (-) | **0.03**  (-) |  |
|  |  |  | $\overline{\boldsymbol{\sigma}_{\boldsymbol{GE}}^{\boldsymbol{2}}}$ | **0**  (-) | **0.12**  (-) | **0.02**  (-) | **0.19** (0.11) | **0.31**  (-) | **0.05**  (-) | **0.05**  (-) | **0**  (-) | **0**  (-) | **0.07**  (-) | **0.08**  (-) | **0** (-) | **0**  (-) | **0.07**  (-) | **0.17**  (-) | **0.09** (0.08) | **0.32**  (-) | **0.28** (0.21) | **0.32** (0.15) | **0.34**  (-) |  |

**Table S9.** Variance decomposition with the model M_FA_s_P_s_ (within-family additive and permanent effect variances)

AIC and BIC criterion are indicated for each model. The estimations of variance components are indicated in bold and their standard errors are given in brackets. The standard error is not computed if the estimated variance value is close to 0. AxE and PxE interaction terms were averaged over environments. If the standard errors were available for each environment, the standard errors associated to the mean values were computed with the following formula: ${SE}_{\bar{\sigma^{2}}}= \sqrt{SE_{\sigma_{Blo19}^{2}}^{2}+ SE_{\sigma_{Smh19}^{2}}^{2} + SE_{\sigma_{Vil19}^{2}}^{2}}$

**Table S10** Additive and genetic variance ratios between M_FG_S_. M_FA_S_ and M_FA_S_P_S_

| **Variance ratio** | **Trait** | **A1D8** | **A1D5** | **A1D9** | **A2D2** | **A2D4** | **A3D1** | **A3D4** | **A3D6** | **A4D3** | **A4D4** | **A4D6** | **A5D1** | **A5D2** | **A5D7** | **A6D3** | **A6D5** | **A6D7** | **A7D3** | **A7D5** | **A7D6** | **Mean** |
| --- | --- | --- | --- | --- | --- | --- | --- | --- | --- | --- | --- | --- | --- | --- | --- | --- | --- | --- | --- | --- | --- | --- |
| $\frac{{\hat{\sigma}^{2}}_{A_{M\_FA_{S}}}}{{\hat{\sigma}^{2}}_{G_{M\_FG_{S}}}}$ | **FLOM** | 1.35 | 0.90 | 0.76 | 1.19 | 1.22 | 1.38 | 1.13 | 1.09 | 1.31 | 2.74 | 0.92 | 1.01 | 1.23 | 1.30 | 1.03 | 1.10 | 1.01 | 1.48 | 0.97 | 1.49 | **1.23** |
|  | **FLOF** | 1.31 | 0.97 | 0.82 | 1.15 | 0.95 | 1.22 | 1.34 | 1.12 | 2.10 | 3.18 | 1.37 | 1.06 | 1.23 | 1.10 | 1.06 | 1.22 | 0.86 | 1.49 | 0.97 | 1.10 | **1.28** |
|  | **ASI** | 0.54 | 1.43 | 0.70 | 0.55 | 0.54 | 0.91 | 0.65 | 0.80 | 0.97 | 0.53 | 0.64 | 0.43 | 0.61 | 0.65 | 0.73 | 0.56 | 0.21 | 5962 | 0.72 | 0.64 | **298.75** |
|  | **PH** | 1.02 | 1.32 | 0.72 | 1.36 | 0.00 | 2.00 | 1.21 | 1.29 | 3.12 | 2.34 | 1.66 | 0.82 | 0.89 | 0.94 | 1.13 | 1.20 | 1.12 | 1.51 | 1.10 | 0.70 | **1.27** |
|  | **GY** | 0.86 | 0.98 | 0.78 | 0.80 | 1.20 | 1.09 | 1.30 | 0.93 | 0.35 | 0.74 | 1.24 | 1.04 | 0.84 | 1.07 | 1.18 | 0.54 | 1.21 | 1.34 | 1.19 | 1.61 | **1.01** |
|  | **H2O** | 0.82 | 0.94 | 0.96 | 0.88 | 0.76 | 0.97 | 1.15 | 0.85 | 1.22 | 1.89 | 1.18 | 1.17 | 0.89 | 0.97 | 0.74 | 1.23 | 0.66 | 2.59 | 1.36 | 0.90 | **1.11** |
|  | **YI** | 0.83 | 1.69 | 0.83 | 0.89 | 1.18 | 1.27 | 1.27 | 0.92 | 0.35 | 0.78 | 0.72 | 0.90 | 0.55 | 1.07 | 0.89 | 0.93 | 1.33 | 1.63 | 1.30 | 1.72 | **1.05** |
| $\frac{{\hat{\sigma}^{2}}_{A_{M\_FA_{S}P_{S}}}}{{\hat{\sigma}^{2}}_{G_{M\_FG_{S}}}}$ | **FLOM** | 1.14 | 0.84 | 0.75 | 1.02 | 0.29 | 0.54 | 0.71 | 0.53 | 0.31 | 0.00 | 0.98 | 0.99 | 1.19 | 0.60 | 0.97 | 1.01 | 0.93 | 0.39 | 0.95 | 0.00 | **0.71** |
|  | **FLOF** | 0.76 | 0.89 | 0.75 | 0.76 | 0.43 | 0.93 | 0.36 | 0.77 | 0.27 | 0.05 | 0.86 | 1.03 | 0.88 | 0.73 | 1.02 | 1.18 | 0.64 | 0.29 | 0.98 | 0.70 | **0.71** |
|  | **ASI** | 0.64 | 0.25 | 0.70 | 0.52 | 0.53 | 0.80 | 0.60 | 0.69 | 0.79 | 0.54 | 0.68 | 0.39 | 0.60 | 0.69 | 0.73 | 0.57 | 0.06 | 469.20 | 0.71 | 0.61 | **24.02** |
|  | **PH** | 1.01 | 0.28 | 0.75 | 1.04 | 11.29 | 2.15 | 1.22 | 0.39 | 0.00 | 0.00 | 0.10 | 0.86 | 0.91 | 0.93 | 0.78 | 0.74 | 0.79 | 0.00 | 1.04 | 0.60 | **1.24** |
|  | **GY** | 0.87 | 0.00 | 0.76 | 0.77 | 0.71 | 1.17 | 0.70 | 0.87 | 0.00 | 0.17 | 1.25 | 1.09 | 0.66 | 0.84 | 1.02 | 0.36 | 0.97 | 0.83 | 0.27 | 0.00 | **0.67** |
|  | **H2O** | 0.82 | 0.79 | 0.97 | 0.78 | 0.65 | 0.97 | 0.19 | 0.62 | 1.08 | 0.63 | 1.18 | 0.42 | 0.84 | 0.85 | 0.75 | 1.19 | 0.65 | 0.00 | 0.91 | 0.76 | **0.75** |
|  | **YI** | 0.86 | 0.81 | 0.87 | 0.84 | 0.89 | 1.21 | 0.31 | 0.76 | 0.00 | 0.09 | 0.29 | 0.79 | 0.00 | 0.81 | 0.45 | 0.72 | 0.87 | 0.00 | 0.21 | 0.00 | **0.54** |
| $\frac{{\hat{\sigma}^{2}}_{A_{M\_FA_{S}P_{S}}}+{\hat{\sigma}^{2}}_{P_{M\_FA_{S}P_{S}}}}{{\hat{\sigma}^{2}}_{G_{M\_FG_{S}}}}$ | **FLOM** | 1.20 | 0.84 | 0.75 | 1.02 | 0.86 | 1.03 | 0.92 | 0.80 | 1.00 | 1.02 | 0.98 | 1.01 | 1.20 | 0.92 | 0.97 | 1.08 | 0.94 | 0.93 | 0.95 | 1.05 | **0.97** |
|  | **FLOF** | 1.00 | 0.90 | 0.76 | 0.98 | 0.79 | 1.02 | 0.84 | 0.90 | 1.00 | 1.00 | 1.06 | 1.03 | 1.07 | 0.92 | 1.02 | 1.18 | 0.66 | 0.94 | 0.98 | 1.01 | **0.95** |
|  | **ASI** | 0.64 | 0.25 | 0.70 | 0.52 | 0.53 | 0.80 | 0.60 | 0.69 | 0.79 | 0.54 | 0.68 | 0.39 | 0.60 | 0.69 | 0.73 | 0.57 | 0.06 | 479.7 | 0.71 | 0.61 | **24.54** |
|  | **PH** | 1.01 | 0.98 | 0.77 | 1.27 | 11.29 | 2.15 | 1.22 | 1.00 | 1.27 | 1.00 | 1.00 | 0.86 | 0.95 | 0.93 | 0.82 | 1.04 | 0.97 | 1.00 | 1.04 | 0.63 | **1.56** |
|  | **GY** | 0.87 | 0.72 | 0.84 | 0.77 | 0.88 | 1.17 | 1.13 | 1.01 | 0.95 | 1.00 | 1.25 | 1.09 | 0.87 | 0.97 | 1.08 | 0.36 | 0.97 | 1.12 | 0.97 | 1.03 | **0.95** |
|  | **H2O** | 0.82 | 0.87 | 0.97 | 0.78 | 0.65 | 0.97 | 0.89 | 0.83 | 1.08 | 1.08 | 1.18 | 0.88 | 0.89 | 0.85 | 0.75 | 1.20 | 0.66 | 1.07 | 1.06 | 0.87 | **0.92** |
|  | **YI** | 0.86 | 0.81 | 0.87 | 0.84 | 0.92 | 1.21 | 1.04 | 0.98 | 0.77 | 0.95 | 0.52 | 0.96 | 0.93 | 0.97 | 1.13 | 0.72 | 0.88 | 1.05 | 0.97 | 1.02 | **0.92** |
| $\frac{{\hat{\sigma}^{2}}_{A_{M\_FA_{S}P_{S}}}}{{\hat{\sigma}^{2}}_{A_{M\_FA_{S}P_{S}}}+{\hat{\sigma}^{2}}_{P_{M\_FA_{S}P_{S}}}}$ | **FLOM** | 0.95 | 1.00 | 0.99 | 1.00 | 0.33 | 0.52 | 0.78 | 0.66 | 0.31 | 0.00 | 1.00 | 0.99 | 1.00 | 0.65 | 1.00 | 0.94 | 0.99 | 0.42 | 1.00 | 0.00 | **0.73** |
|  | **FLOF** | 0.76 | 1.00 | 0.99 | 0.77 | 0.54 | 0.92 | 0.43 | 0.86 | 0.27 | 0.05 | 0.81 | 0.99 | 0.82 | 0.80 | 1.00 | 1.00 | 0.98 | 0.31 | 1.00 | 0.70 | **0.75** |
|  | **ASI** | 1.00 | 1.00 | 1.00 | 1.00 | 1.00 | 1.00 | 1.00 | 1.00 | 1.00 | 1.00 | 1.00 | 1.00 | 1.00 | 1.00 | 1.00 | 1.00 | 1.00 | 0.98 | 1.00 | 1.00 | **1.00** |
|  | **PH** | 1.00 | 0.28 | 0.98 | 0.82 | 1.00 | 1.00 | 1.00 | 0.39 | 0.00 | 0.00 | 0.10 | 1.00 | 0.96 | 1.00 | 0.95 | 0.71 | 0.81 | 0.00 | 1.00 | 0.94 | **0.70** |
|  | **GY** | 1.00 | 0.00 | 0.91 | 1.00 | 0.81 | 1.00 | 0.62 | 0.86 | 0.00 | 0.17 | 1.00 | 1.00 | 0.77 | 0.86 | 0.94 | 1.00 | 1.00 | 0.74 | 0.28 | 0.00 | **0.70** |
|  | **H2O** | 1.00 | 0.91 | 1.00 | 1.00 | 1.00 | 1.00 | 0.22 | 0.75 | 1.00 | 0.58 | 1.00 | 0.48 | 0.95 | 1.00 | 1.00 | 1.00 | 0.98 | 0.00 | 0.86 | 0.87 | **0.83** |
|  | **YI** | 1.00 | 1.00 | 1.00 | 1.00 | 0.98 | 1.00 | 0.29 | 0.77 | 0.00 | 0.10 | 0.56 | 0.82 | 0.00 | 0.83 | 0.40 | 1.00 | 1.00 | 0.00 | 0.22 | 0.00 | **0.60** |

${\hat{\sigma}^{2}}_{G_{M\_FG_{S}}}$ is the within-family genetic variance estimated with $M\_FG_{S}$, ${\hat{\sigma}^{2}}_{A_{M\_FA_{S}}}$ is the within-family additive variance estimated with $M\_FA_{S}$, ${\hat{\sigma}^{2}}_{A_{M\_FA_{S}P_{S}}}$ is the within-family additive variance estimated with $M\_FA_{S}P_{S}$ and ${\hat{\sigma}^{2}}_{P_{M\_FA_{S}P_{S}}}$is the within-family permanent effect variance estimated with $M\_FA_{S}P_{S}$

|  |  | **A1** | | | **A2** | | **A3** | | | **A4** | | | **A5** | | | **A6** | | | **A7** | | |
| --- | --- | --- | --- | --- | --- | --- | --- | --- | --- | --- | --- | --- | --- | --- | --- | --- | --- | --- | --- | --- | --- |
| **Trait** | **UC Type** | **A1D8** | **A1D5** | **A1D9** | **A2D2** | **A2D4** | **A3D1** | **A3D4** | **A3D6** | **A4D3** | **A4D4** | **A4D6** | **A5D1** | **A5D2** | **A5D7** | **A6D3** | **A6D5** | **A6D7** | **A7D3** | **A7D5** | **A7D6** |
| **GY** | **UC1** | 11.89 | 12.16 | 12.29 | 11.55 | 11.93 | 11.84 | 12.60 | 12.04 | 12.08 | 11.91 | 12.20 | 12.63 | 12.32 | 12.88 | 11.02 | 11.29 | 11.42 | 11.34 | 10.83 | 11.60 |
|  | **UC2** | 11.89 | 11.75 | 12.25 | 11.55 | 11.86 | 11.84 | 12.36 | 11.99 | 11.47 | 11.67 | 12.20 | 12.63 | 12.22 | 12.81 | 11.00 | 11.29 | 11.42 | 11.16 | 10.40 | 9.65 |
| **YI** | **UC1** | 11.08 | 11.92 | 11.65 | 11.72 | 12.11 | 12.12 | 12.31 | 12.02 | 12.46 | 12.61 | 12.78 | 12.59 | 12.56 | 12.76 | 10.78 | 11.31 | 10.87 | 10.90 | 10.74 | 10.66 |
|  | **UC2** | 11.64 | 11.57 | 12.05 | 11.86 | 12.23 | 12.16 | 12.83 | 12.43 | 12.32 | 12.19 | 12.01 | 12.48 | 12.09 | 12.72 | 11.44 | 12.13 | 11.71 | 11.48 | 11.04 | 11.78 |

**Table S11** Usefulness criterion values for the different families

For the family $k$, $UC_{1k}= \hat{\mu_{k}}+ih\hat{\sigma_{Gk}}$, $UC_{2k}= \hat{\mu_{k}}+ih\hat{\sigma_{Ak}}$ and ${UC}_{bis2k}= \hat{\mu_{k}}+ih{\hat{\sigma_{Ak}}}^{*}$. $\hat{\mu_{k}}$ is the mean performance of the family, $i$ the intensity of selection ($i$=2.07), $h$ is the selection accuracy ($h$ = 1) and $\hat{\sigma_{Gk}}= \hat{\sigma_{Ak}}+\hat{\sigma_{Pk}}$ with $\hat{\sigma_{Ak}}$ and $\hat{\sigma_{Pk}}$ the additive and permanent effect variances of the family estimated with M_FA_S_P_S_.

**Table S12** Theoretical and observed diversity in each family

| **Family** | $\boldsymbol{H}\boldsymbol{e}_{\boldsymbol{O}}$ | $\boldsymbol{H}\boldsymbol{e}_{\boldsymbol{E}}$ | $\frac{\boldsymbol{H}\boldsymbol{e}_{\boldsymbol{O}}\boldsymbol{- H}\boldsymbol{e}_{\boldsymbol{E}}}{\boldsymbol{H}\boldsymbol{e}_{\boldsymbol{E}}}\boldsymbol{(\%)}$ |
| --- | --- | --- | --- |
| A1D8 | 0.156 | 0.154 | 1% |
| A1D5 | 0.153 | 0.161 | -5% |
| A1D9 | 0.133 | 0.143 | -7% |
| A2D2 | 0.153 | 0.150 | 2% |
| A2D4 | 0.136 | 0.145 | -6% |
| A3D1 | 0.145 | 0.152 | -5% |
| A3D4 | 0.148 | 0.154 | -4% |
| A3D6 | 0.154 | 0.159 | -3% |
| A4D3 | 0.137 | 0.152 | -10% |
| A4D4 | 0.136 | 0.148 | -8% |
| A4D6 | 0.139 | 0.149 | -7% |
| A5D1 | 0.152 | 0.156 | -3% |
| A5D2 | 0.165 | 0.162 | 2% |
| A5D7 | 0.147 | 0.157 | -6% |
| A6D3 | 0.141 | 0.146 | -3% |
| A6D5 | 0.156 | 0.158 | -1% |
| A6D7 | 0.136 | 0.149 | -9% |
| A7D3 | 0.160 | 0.158 | 1% |
| A7D5 | 0.164 | 0.162 | 1% |
| A7D6 | 0.157 | 0.156 | 1% |

$\boldsymbol{H}\boldsymbol{e}_{\boldsymbol{O}}\boldsymbol{=}\frac{\boldsymbol{1}}{\boldsymbol{m}}\sum_{\boldsymbol{i=1}}^{\boldsymbol{m}} \boldsymbol{2}\boldsymbol{p}_{\boldsymbol{i}_{\boldsymbol{O}}}\boldsymbol{(1-}\boldsymbol{p}_{\boldsymbol{i}_{\boldsymbol{O}}}\boldsymbol{)}$ is the observed Nei index where $\boldsymbol{m}$ is the number of markers and $\boldsymbol{p}_{\boldsymbol{i}_{\boldsymbol{O}}}$ is the observed frequency of the referent allele for the marker $\boldsymbol{i}$ in a given family. $\boldsymbol{H}\boldsymbol{e}_{\boldsymbol{E}}\boldsymbol{=}\frac{\boldsymbol{1}}{\boldsymbol{m}}\sum_{\boldsymbol{i=1}}^{\boldsymbol{m}} {\boldsymbol{2}\boldsymbol{p}}_{\boldsymbol{i}_{\boldsymbol{E}}}\boldsymbol{(1-}\boldsymbol{p}_{\boldsymbol{i}_{\boldsymbol{E}}}\boldsymbol{)}$ is the expected Nei index where $\boldsymbol{p}_{\boldsymbol{i}_{\boldsymbol{E}}}$ is the expected frequency of the referent allele for the marker $\boldsymbol{i}$ in absence of selection and genetic drift. $\boldsymbol{p}_{\boldsymbol{i}_{\boldsymbol{E}}}$was computed taking into account the parental genotypes and the cross type used to create families (BC1).


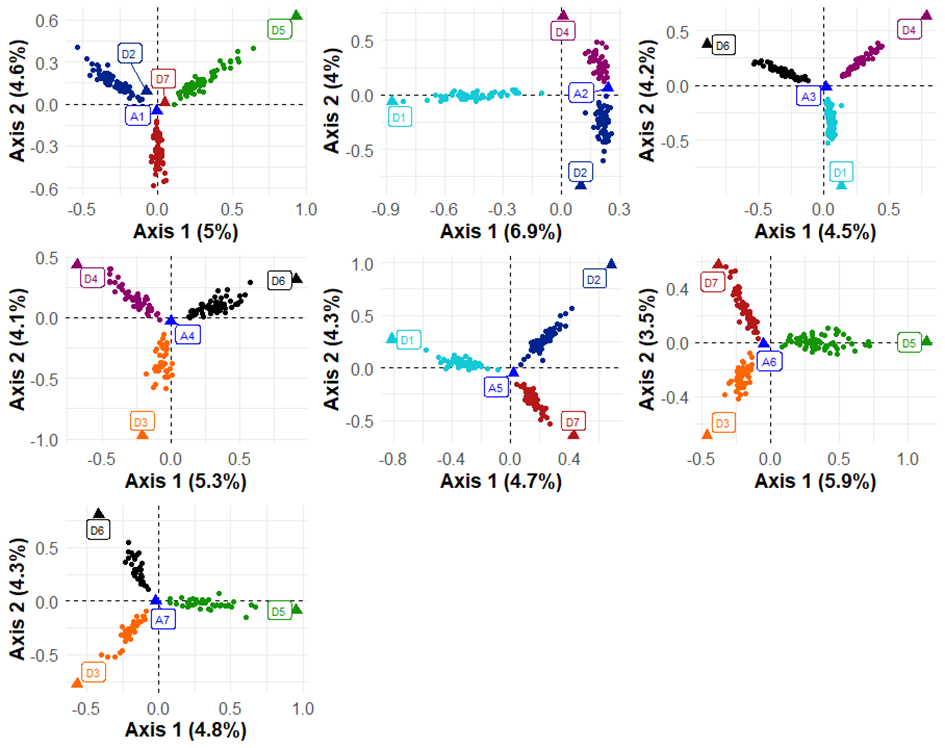


**Fig S1. Multi correspondence analysis (MCA) based on BC1S2 individual genotypic information**. MCA analyses were performed using a 25K genotyping matrix (coded with IUPAC standard and “failed” if the genotyping information was not available). Each MCA analysis included one of the recipient line (blue triangle) and BC1S2 individuals derived from this recipient line (dots colored by declared donor line). Declared donor lines are treated as supplemental individuals (triangles). 16 individuals were removed before analysis because of high illegitimate rates.


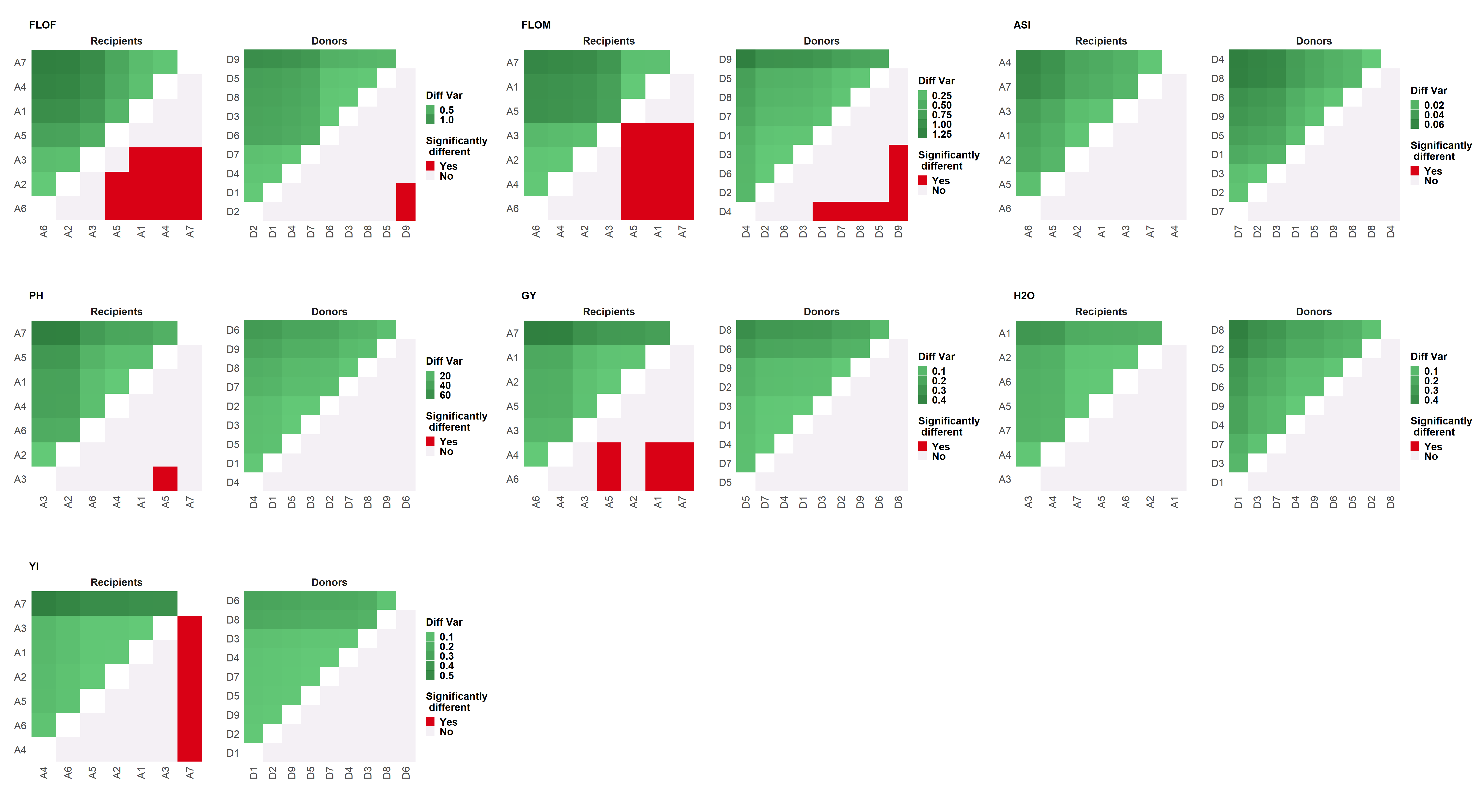


**Fig S2. Recipient and donor pairwise comparisons of genetic variance values (model M_FG_S_).** For each trait, the mean genetic variance of the families derived from the same recipient or donor was computed. The recipients and the donors were ranked according to this value. **Upper triangle:** mean differences of genetic variances between two recipients or two donors (Diff Var). **Lower triangle**: Significance of the variance difference between each pair of recipients or donors. For each pair, a contrast was defined as presented in **File S3** and its nullity was tested. The difference is declared significant, after a Benjamini-Hochberg multiple testing correction to control the FDR level at a nominal level of 0.05 (see **File S3** for further details).


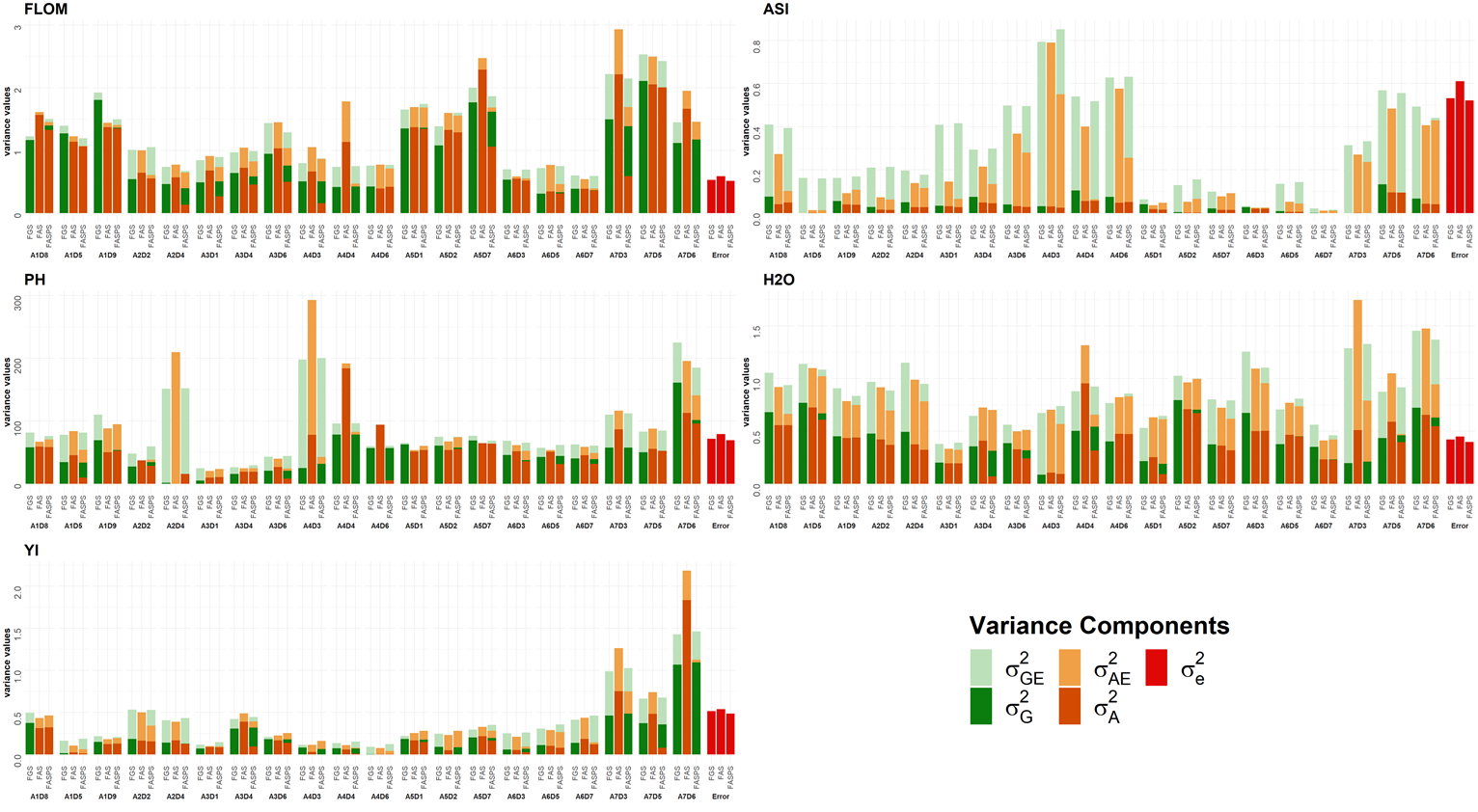


**Fig S3 Within-family genetic and additive variance estimation with the models M_FG_S_. M_FAs and M_FA_S_P_S_ for FLOM. ASI. PH. H2O and YI.** For M_FAs and M_FA_S_P_S_, within-family additive variances are indicated in orange. The within-family genetic (model M_FG_S_) and permanent effect (model M_FA_S_P_S_) variances are in dark green. The interaction (AxE: light orange and GxE: light green) and error (red) terms are trial specific and represented by their respective average values

**
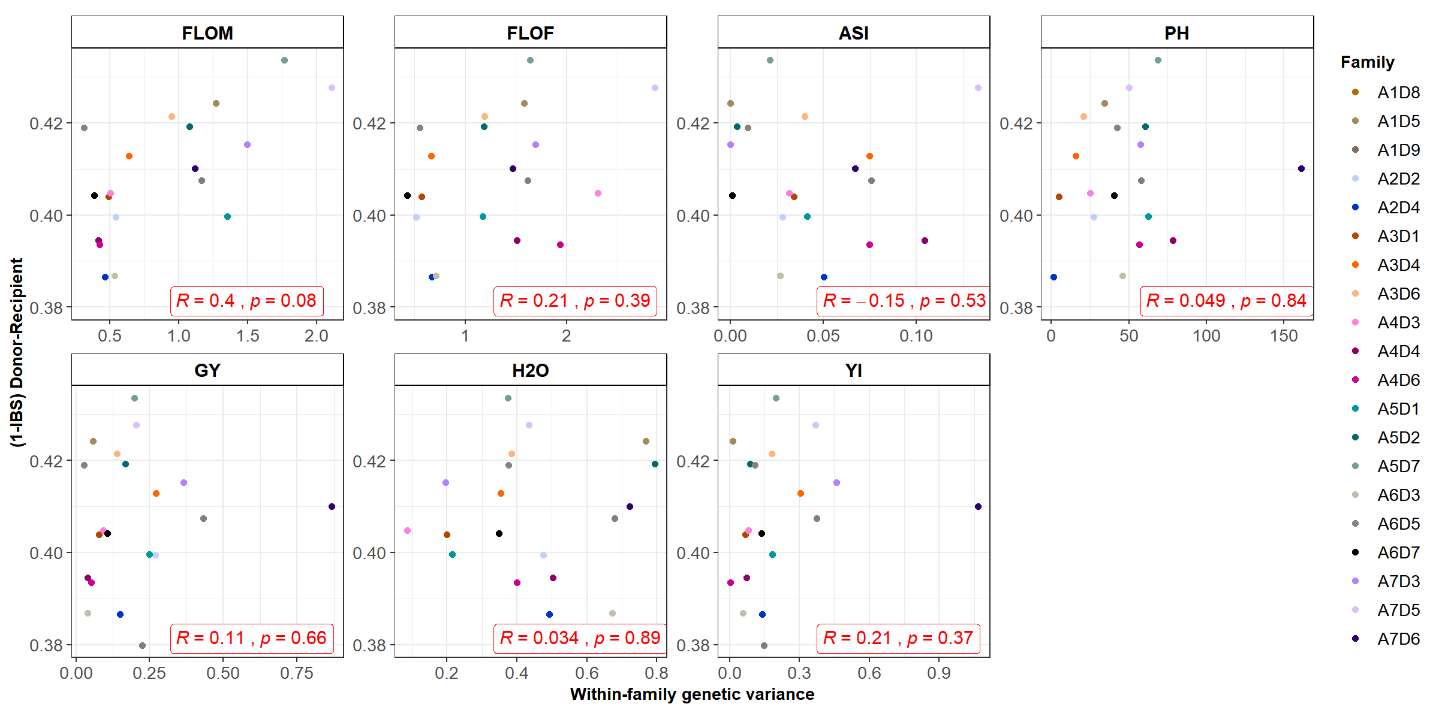
Fig S4 Correlation between the genetic distances between parents and the within-family genetic variances.** For each parent pair, the genetic distance was computed as 1-IBS (Identity-By-State). For each trait, R is the Pearson correlation coefficient value between the genetic distance between the parents and the within-family genetic variance and p is the p-value obtained for the associated student statistical test.

**References – Supplemental Material**

Butler DG, Cullis BR, Gilmour AR, Gogel BJ (2009) ASREML-R Reference Manual. Release 3.0. Technical Report, Queensland Department of Primary Industries, Australia.

Ganal MW, Durstewitz G, Polley A, et al (2011) A Large Maize (Zea mays L.) SNP Genotyping Array: Development and Germplasm Genotyping, and Genetic Mapping to Compare with the B73 Reference Genome. PLoS ONE 6:e28334. https://doi.org/10.1371/journal.pone.0028334

Gonen S, Wimmer V, Gaynor RC, et al (2018) A heuristic method for fast and accurate phasing and imputation of single-nucleotide polymorphism data in bi-parental plant populations. Theor Appl Genet 131:2345–2357. https://doi.org/10.1007/s00122-018-3156-9

Gouesnard B, Negro S, Laffray A, et al (2017) Genotyping-by-sequencing highlights original diversity patterns within a European collection of 1191 maize flint lines, as compared to the maize USDA genebank. Theor Appl Genet 130:2165–2189. https://doi.org/10.1007/s00122-017-2949-6

Rincent R, Laloë D, Nicolas S, et al (2012) Maximizing the Reliability of Genomic Selection by Optimizing the Calibration Set of Reference Individuals: Comparison of Methods in Two Diverse Groups of Maize Inbreds (Zea mays L.). Genetics 192:715–728. https://doi.org/10.1534/genetics.112.141473

Vitezica ZG, Legarra A, Toro MA, Varona L (2017) Orthogonal Estimates of Variances for Additive, Dominance, and Epistatic Effects in Populations. Genetics 206:1297–1307. https://doi.org/10.1534/genetics.116.199406
